# Early detection and a treatment bundle strategy for postpartum haemorrhage: a mixed-methods process evaluation

**DOI:** 10.1016/S2214-109X(24)00454-6

**Published:** 2025-01-29

**Authors:** Meghan A Bohren, Suellen Miller, Kristie-Marie Mammoliti, Hadiza Galadanci, Sue Fawcus, Neil Moran, G Justus Hofmeyr, Zahida Qureshi, Fadhlun Alwy Al-Beity, Gillian Forbes, Shahinoor Akter, Alfred Osoti, George Gwako, Thiago Melo Santos, Cherrie Evans, Aminu Ado Wakili, Maisaratu Bakari, Idris Usman Takai, Mohammad Umar, Mandisa Singata-Madliki, Elani Muller, Sibongile Mandondo, Jenipher Okore, Akwinata Banda, Masumbuko Sambusa, Kulandaipalayam N Sindhu, Leanne Beeson, Christina Louise Easter, Adam Devall, A Metin Gülmezoglu, Fernando Althabe, Olufemi T Oladapo, Ioannis Gallos, Arri Coomarasamy, Fabiana Lorencatto

**Affiliations:** a Gender and Women's Health Unit, Nossal Institute for Global Health, School of Population and Global Health, University of Melbourne, Melbourne, VIC, Australia; b Department of Obstetrics and Reproductive Sciences, School of Medicine, University of California San Francisco, CA, USA; c College of Medicine and Health, University of Birmingham, Birmingham, UK; d African Center of Excellence for Population Health and Policy, College of Health Sciences, Bayero University, Kano, Nigeria; e Department of Obstetrics and Gynaecology, University of Cape Town, Cape Town, South Africa; f KwaZulu-Natal Department of Health, Pietermaritzburg, South Africa; g Department of Obstetrics and Gynaecology, Nelson Mandela School of Medicine, University of KwaZulu-Natal, Durban, South Africa; h Effective Care Research Unit, University of the Witwatersrand, Johannesburg, South Africa; i Walter Sisulu University, East London, South Africa; j Department of Obstetrics and Gynecology, University of Botswana, Gaborone, Botswana; k Department of Obstetrics and Gynecology, Faculty of Health Sciences, University of Nairobi, Nairobi, Kenya; l Department of Obstetrics and Gynecology, Muhimbili University of Health and Allied Sciences, Dar es Salaam, Tanzania; m Centre for Behaviour Change, University College London, London, UK; n John Richards Centre for Rural Ageing Research, La Trobe Rural Health School, La Trobe University, Wodonga VIC, Australia; o Maternal and Newborn Health Unit, Technical Leadership and Innovation, Jhpiego, Baltimore, MD, USA; p Johns Hopkins University, Baltimore, MD, USA; q Department of Obstetrics and Gynaecology, Faculty of Clinical Sciences, College of Health Sciences, Bayero University, Kano, Nigeria; r Department of Obstetrics and Gynaecology, General Hospital Katsina, Hospital Services Management Board, Katsina, Nigeria; s Effective Care Research Unit, East London, South Africa; t Institute for Applied Health Research, University of Birmingham, Birmingham, UK; u Concept Foundation, Geneva, Switzerland; v UNDP–UNFPA–UNICEF–WHO–World Bank Special Programme of Research, Development and Research Training in Human Reproduction (HRP), Department of Sexual and Reproductive Health and Research, World Health Organization, Geneva, Switzerland

## Abstract

**Background:**

Postpartum haemorrhage is a leading cause of maternal mortality. A multicountry, cluster-randomised trial (E-MOTIVE) demonstrated a 60% reduction in adverse postpartum haemorrhage outcomes. The E-MOTIVE intervention included early postpartum haemorrhage detection using calibrated blood-collection drapes, followed by a postpartum haemorrhage treatment bundle (ie, uterine massage, oxytocics, tranexamic acid, intravenous fluids, examination and escalation [MOTIVE]), supported by implementation strategies. We report a mixed-methods process evaluation assessing the implementation of the E-MOTIVE intervention in Kenya, Nigeria, South Africa, and Tanzania.

**Methods:**

In this mixed-methods process evaluation, data sources were observations of health workers providing clinical care to pregnant women and pregnant people during vaginal birth and postpartum haemorrhage at intervention sites, and surveys and qualitative interviews with health workers at intervention and control sites. Intervention sites received the calibrated drapes, MOTIVE bundle, and implementation strategies and control sites used uncalibrated drapes. Primary implementation outcomes included fidelity, adoption, adaptation, acceptability, feasibility, and contamination to the calibrated drape, MOTIVE bundle, and implementation strategies.

**Findings:**

Between June 1, 2022, and Jan 31, 2023, 2578 births were observed, 295 pregnant women and people had postpartum haemorrhage, 47 qualitative interviews were done, and 889 surveys were completed. Fidelity to calibrated drape use was high (birth observations 2578 [100%] of 2578; survey 451 [98·3%] of 459). Among health workers, calibrated drape acceptability was high; however, they reported barriers to pregnant women's and people's acceptability. Fidelity to postpartum haemorrhage treatment bundle delivery was high (birth observations 286 [96·9%] of 295), with moderate to high fidelity in median time from postpartum haemorrhage diagnosis to final treatment initiation (≤15 min initiation time in 191 [66·8%] of 295 birth observations, 16–20 min in 42 [14·7%] birth observations), and high acceptability and feasibility. Research midwives participated in clinical assessments after birth and bundle delivery in some sites (mixed fidelity).

**Interpretation:**

This process evaluation shows generally high levels of fidelity, feasibility, and acceptability of the calibrated drape and treatment bundle across evaluation methods and countries. The E-MOTIVE intervention should be included in national policies, with consideration for health workforce, supplies, and medication issues, which might need addressing for successful implementation.

**Funding:**

The Bill and Melinda Gates Foundation and the UNDP–UNFPA–UNICEF–WHO–World Bank Special Programme of Research, Development and Research Training in Human Reproduction, a co-sponsored programme of WHO.

**Translation:**

For the Swahili translation of the abstract see Supplementary Materials section.

## Introduction

Postpartum haemorrhage, referring to blood loss ≥500 mL within 24 h after birth,^1^ accounts for 27% of maternal deaths worldwide.^2^, ^3^ When postpartum haemorrhage occurs in people giving birth in hospitals, there are three challenges to timely detection and appropriate management.^4^ First, postpartum haemorrhage is often detected late or missed entirely. Visual estimation is typically used to assess postpartum blood loss, but is inaccurate, difficult to implement, and varies substantially between health workers.^5^, ^6^ Second, effective postpartum haemorrhage management interventions exist,^1^, ^7^, ^8^, ^9^, ^10^, ^11^, ^12^ but barriers include inconsistent and delayed use, such as sequential administration of interventions or waiting to see if one intervention is effective before administering another intervention. Third, WHO's postpartum haemorrhage recommendations^1^, ^7^, ^8^, ^9^, ^10^, ^11^, ^12^ are poorly implemented, especially in low-income and middle-income countries, due to limited knowledge and skills, health workforce shortages, and medication and supply stock-outs.^13^, ^14^

**Research in context**

**Evidence before this study**

An international technical consultation to develop the concept of treatment bundles for postpartum haemorrhage was conducted in 2017, based on the WHO guideline recommendations. Two treatment bundles were proposed: first response to postpartum haemorrhage consisting of uterotonics, isotonic crystalloids, tranexamic acid, and uterine massage, and response to refractory postpartum haemorrhage compressive measures, non-pneumatic antishock garment, and intrauterine balloon tamponade. Both treatment bundles were to be supported by advocacy, training, teamwork, communication, and use of best clinical practices. We searched PubMed from database inception to Oct 7, 2024, using the terms ((“postpartum hemorrhage” OR “postpartum haemorrhage” OR “obstetric hemorrhage” OR “obstetric haemorrhage”) AND “bundle”). Most publications were case reports or observational, cross-sectional, or quality improvement studies. The E-MOTIVE trial and associated research programme was the only randomised trial evaluating care bundles for first-response or refractory treatment of postpartum haemorrhage. The E-MOTIVE trial was a multi-country, parallel cluster-randomised trial. While designing the E-MOTIVE intervention and implementation strategies, we conducted a Cochrane qualitative evidence synthesis to explore factors influencing postpartum haemorrhage prevention, detection, and management, and mixed-methods formative research to improve understanding of health workers’ knowledge and practices of postpartum haemorrhage detection and management, and perceptions of future implementation of a new postpartum haemorrhage treatment bundle. We presented the influencing factors and draft implementation strategies at stakeholder consultation and design workshops in each country in 2021 to discuss feasibility, acceptability, and local adaptations, then piloted and evaluated the intervention and implementation strategies in three hospitals per country. The E-MOTIVE intervention included early postpartum haemorrhage detection using a calibrated blood-collection drape, followed by a postpartum haemorrhage treatment bundle (uterine massage, oxytocic drugs, tranexamic acid, intravenous fluids, examination and escalation [MOTIVE]) administered within 15 min of postpartum haemorrhage diagnosis. The E-MOTIVE intervention was supported by an implementation strategy consisting of: postpartum haemorrhage trolleys or carry-cases (with all necessary medications and supplies); simulation-based, on-site training; postpartum haemorrhage champions (midwives and doctors driving change in study hospitals); and audit and feedback of actionable data to health workers.

**Added value of this study**

Alongside the E-MOTIVE trial, we conducted a parallel, mixed-methods process evaluation to explore intervention implementation. This process evaluation provides additional context to support interpretation of the high-profile E-MOTIVE trial results, and identifies specific considerations to inform scalability, sustainability, and roll-out. We assess the implementation outcomes of fidelity, adoption, adaptation, acceptability, and feasibility of the calibrated drape, treatment bundle, and implementation strategies. We integrate data from three sources: observations of health workers providing clinical care to pregnant women and people throughout vaginal birth and managing postpartum haemorrhage at intervention sites, and qualitative interviews and quantitative surveys with health workers at intervention and control sites. This data integration provides a robust picture of both objective and self-reported behaviours of health workers engaged in the E-MOTIVE trial. The results of this process evaluation improve understanding of the mechanisms of action in the E-MOTIVE trial (ie, the processes by which the E-MOTIVE clinical intervention and implementation strategies influenced trial outcomes and practice changes), which might prove useful to roll-out and scale-up the intervention and implementation strategies beyond the E-MOTIVE trial.

**Implications of all the available evidence**

The E-MOTIVE trial identified a 60% relative reduction in the primary composite outcome of severe postpartum haemorrhage (blood loss ≥1000 mL), laparotomy for bleeding, or maternal death from bleeding. The process evaluation shows high fidelity of trial implementation, and the calibrated drape, treatment bundle, and implementation strategies were acceptable and feasible. These results confirmed programme logic about how and why E-MOTIVE was likely to work: early postpartum haemorrhage detection and bundled treatment can improve outcomes when simulation-based, on-site training is facilitated; supplies and equipment are accessible; staff are supported; health worker roles and responsibilities are clear; and there is protected time and agency to deliver effective care.

To address these challenges, we designed the E-MOTIVE intervention (appendix 2 pp 3–13, 18), evaluating it in a multicountry, parallel cluster-randomised trial in 78 hospitals in Kenya, Nigeria, South Africa, and Tanzania.^4^ Trial results showed a 60% relative reduction in the primary composite outcome (ie, severe postpartum haemorrhage defined as blood loss ≥1000 mL, laparotomy for bleeding, or maternal death from bleeding), and a 58% increase in postpartum haemorrhage detection.^4^ The E-MOTIVE intervention included a calibrated blood-collection drape for early postpartum haemorrhage detection (exclusively for those who gave birth vaginally), and first-response treatment bundle^15^ comprising uterine massage, oxytocic drugs, tranexamic acid, intravenous fluids, examination and escalation (MOTIVE).^4^ The MOTIVE treatment bundle was administered concurrently or in rapid succession, from within 15 min of diagnosis to final treatment initiation, to all pregnant women and people diagnosed with postpartum haemorrhage. For trial measurement, calibrated drapes were removed after 1 h (2 h if bleeding continued) and weighed on a digital scale by research staff. In control hospitals, uncalibrated drapes were used and similarly weighed for trial measurement.

Potential implementation challenges were identified during pre-trial formative research.^5^, ^13^, ^14^, ^16^, ^17^ We used behavioural and implementation science theories and frameworks^18^, ^19^ to identify implementation strategies that addressed barriers and reinforced enablers, which are described elsewhere.^17^ Implementation strategies consisted of postpartum haemorrhage trolleys or carry-cases with necessary medications and supplies (except for oxytocin, which requires refrigeration); simulation-based, on-site training; postpartum haemorrhage champions (midwives and doctors driving change); and audit and feedback of actionable data. For trial implementation, intervention sites were provided with calibrated drapes, postpartum haemorrhage trolleys or carry-cases (drugs and supplies were not provided), on-site training, support for postpartum haemorrhage champions, and monthly audit newsletters. Control sites provided usual care: visual estimation of blood loss (uncalibrated drapes provided to quantify blood loss for trial purposes), and postpartum haemorrhage management per local guidelines.

We designed a parallel process evaluation to assess the extent to which the E-MOTIVE intervention and implementation strategies were conducted as intended, and to inform interpretation of trial results, scalability, and sustainability. We focused on the implementation outcomes of fidelity, adoption, adaptation, acceptability, feasibility, and contamination.^20^

## Methods

### Study design

This study is a mixed-methods process evaluation using a cross-sectional observational design. We followed UK Medical Research Council guidance advocating a mixed-methods approach to balance data breadth and depth.^21^ Data sources were observations of health workers providing clinical care to pregnant women and people throughout vaginal birth and postpartum haemorrhage management (intervention hospitals), and qualitative interviews and cross-sectional surveys (intervention and control hospitals; table 1). There were 39 intervention sites (seven in Kenya, 19 in Nigeria, seven in South Africa, and six in Tanzania), and 39 control sites (seven in Kenya, 19 in Nigeria, seven in South Africa, and six in Tanzania). Data were collected mid-trial, between months 3–7 of the trial intervention, staggered between June 1, 2022, and Jan 31, 2023. We report using the StarI checklist^22^ (appendix 2 pp 14–17). Appendix 2 (p 2) contains an ethics statement; in brief, approvals were obtained from ethics committees in each study country, from the University of Birmingham, the University of Melbourne, and WHO. The trial was prospectively registered with ClinicalTrials.gov, NCT04341662.

**Table 1:** Operational definitions of implementation outcomes of interest

	**Operational definition**	**Data source**
Fidelity, adoption, and adaptation	Extent to which the calibrated drape, MOTIVE treatment bundle, and implementation strategies were used as intended by health workers in participating sites (fidelity and adoption), and any modifications made to the E-MOTIVE intervention to adapt to the study context and adhere to the study protocol (adaptation).	Observation, qualitative interviews, and survey
Acceptability	Extent to which using the calibrated drape, MOTIVE bundle, and implementation strategies were acceptable to health workers, which includes affective attitudes and perceived benefits towards the intervention, which either facilitate or hinder its use.	Qualitative interviews and survey
Feasibility	Building on acceptability, documenting additional barriers and enablers affecting the use of the calibrated drape, MOTIVE bundle, and implementation strategies as intended, particularly those related to physical and social opportunity (eg, available resources, time, staffing, teamwork, and communication).	Qualitative interviews and survey
Contamination and treatment differentiation	External or competing activities that interact with E-MOTIVE (eg, other research studies or quality improvement initiatives); extent of communication between intervention and control sites and spillover of intervention to control sites; extent to which postpartum haemorhhage detection and management differed between intervention and control sites; all collectively leading to potential loss of differentiation between intervention versus control sites.	Qualitative interviews and survey

Implementation strategies were audit and feedback; postpartum haemorrhage champions; postpartum haemorrhage trolley or carry case; and simulation-based, on-site training. MOTIVE=uterine massage, oxytocic drugs, tranexamic acid, intravenous fluids, examination and escalation.

### Participants

Study hospitals were secondary-level hospitals that were geographically and administratively distinct from each other, had between 1000 and 5000 vaginal births per year, and were able to provide comprehensive obstetrical care with the ability to perform surgery for postpartum haemorrhage. Women were eligible for inclusion in the broader E-MOTIVE trial if they had a vaginal birth in study hospitals.

Midwives, nurses, and doctors working on labour wards were purposively recruited for qualitative interviews using maximum variation sampling to ensure diversity. The research team facilitated contact with potential participants at their workplace, provided study information, invited participation, and obtained written informed consent.

Country research teams also recruited midwives, nurses, and doctors working on labour wards who could complete a survey in English, and written informed consent was obtained electronically via the survey platform.

Implementation midwives were employed by the research team to support trial management and training, and external trainers delivered initial training and 1-month and 3-month follow-up support. Research midwives were employed by the research team at all intervention and control sites for trial outcome assessment (ie, weighing drapes and data collection). Staff midwives refer to midwives employed by the hospitals to provide clinical care.

### Procedures

Observations of health workers providing clinical care to pregnant women and people throughout vaginal birth and postpartum haemorrhage management were conducted at all 39 intervention sites (control site observations to be reported elsewhere). Observations were pragmatic: data were collected on as many pregnant women and people admitted for vaginal birth during observation period as possible, without a prespecified sample size, and with hospital-level consent. Data collectors continuously observed care provided to pregnant women and people during birth focusing on calibrated drape use, clinical assessments after birth, and diagnosis and treatment of postpartum haemorrhage, if it occurred. The period of interest was from time of birth until drape removal. Study hospitals gave approvals for observations to occur; health workers and pregnant women and people were informed about the purpose and procedures of the observation, but individual consent was not obtained.

A structured observation guide was piloted and refined during pre-trial adaptive cycles^16^, ^17^ and included: pregnant women's and people's sociodemographic and obstetric characteristics, calibrated drape use, postpartum clinical assessments (ie, blood pressure, pulse, uterine tone, vaginal blood flow, and cumulative vaginal blood loss reading against calibration lines), postpartum haemorrhage diagnosis, and postpartum haemorrhage treatments administered, all with start and stop timestamps (appendix 2 pp 101–29). We recorded who diagnosed postpartum haemorrhage and implemented postpartum haemorrhage treatments, to assess the feasibility of E-MOTIVE implementation by midwives or nurses, or if doctors were required, and the feasibility of implementation by existing health workers or necessary involvement of research midwives. There were 33 observers who were E-MOTIVE implementation or research midwives; none were employed by study hospitals. Some observers conducted observations at more than one intervention site. Observers were trained not to intervene in clinical care, unless an emergency arose and clinical staff requested help. Data were recorded using paper-based forms and entered into RedCap by research midwives. Data consistency checks were done daily by K-MM, reviewing RedCap entries for clinical coherence and missing data.

Qualitative interviews were conducted in two intervention hospitals and one control hospital per country (12 hospitals in total), in trial month 6 to allow for intervention embedding. We aimed to recruit eight participants per intervention hospital and four per control hospital. The qualitative interview sample was based on the principles of informational power, which suggests that sample sufficiency, quality of collected data, and variability in experience are more important than the number of participants.^23^

Intervention site participants were asked about postpartum haemorrhage detection and management, specifically calibrated drapes and the MOTIVE treatment bundle (to assess fidelity and adoption), adaptations made to the drape or bundle, perceptions of the E-MOTIVE intervention (to assess acceptability), and factors affecting uptake of the E-MOTIVE intervention (to assess feasibility). Acceptability and feasibility questions were structured using the Capability, Opportunity, and Motivation model of Behaviour Change (COM-B) model.^18^ Similar questions about fidelity, adaptation, and acceptability were asked about the postpartum haemorrhage trolley or carry-case, training, introduction of postpartum haemorrhage champions, and conduct of audit and feedback sessions. To explore potential contamination between intervention and control hospitals, intervention hospital participants were asked about engagement with colleagues in control hospitals, other research projects, and policy changes. Control hospital participants were asked about postpartum haemorrhage detection and management practices, looking for potential contamination. Topic guides were piloted and revised ahead of data collection (appendix 2 pp 42–49).

Qualitative interviews were conducted face to face by country research teams, lasted 18–75 min, were audio-recorded and transcribed verbatim using Otter.ai if in English, or manually transcribed and translated for other languages. Otter.ai transcripts were checked by either MAB, FL, SA, or GF. Team-based inductive thematic analysis was done using NVivo (version 14) by social scientists and midwives, then interpreted by country research teams made up of doctors (HG, SF, NM, GJH, ZQ, FAA-B, AO, and GG), midwives (SMi, K-MM, CE, MS-M, EM, and JO), and social scientists (MAB, GF, SA, and FL). Themes were deductively mapped to implementation outcomes and acceptability and feasibility themes were mapped to COM-B.^18^, ^24^ Analysis was sequential: first by country, then across countries and trial arms.

We conducted a survey in all intervention and control hospitals around trial month 6. The target sample size was ten participants per site, purposively sampled by the country research teams with no refusals. An individualised email link was sent using SmartSurvey, with an electronic information sheet and consent form. Survey questions (appendix 2 pp 50–100) aligned to qualitative interview guides, with multiple choice and Likert scale responses. Intervention site surveys included participant's sociodemographics; use and availability of the calibrated drape, MOTIVE treatment bundle, and implementation strategies (to assess fidelity); and beliefs about the calibrated drape, MOTIVE bundle, and implementation strategies (to assess acceptability and feasibility). Control hospital surveys included participant's sociodemographics and postpartum haemorrhage detection and management practices and beliefs (to assess contamination).

We integrated data across sources to interpret contexts, processes, and impacts of the E-MOTIVE intervention. We started with fidelity outcomes assessed in observations and used qualitative and survey data to enhance understanding of how feasibility and acceptability of the calibrated drape, MOTIVE treatment bundle, and implementation strategies might have influenced fidelity. For observations and surveys, we classified implementation outcomes as high (≥80%), moderate (≥50% to <80%), poor (<50%), or mixed (country-level differences in implementation outcomes).^25^

### Outcomes

Primary implementation outcomes included fidelity, adoption, adaptation, acceptability, and feasibility of the calibrated drape and MOTIVE bundle and contamination between intervention and control hospitals. Results and implementation outcomes are presented across three domains: postpartum haemorrhage detection using calibrated drape, postpartum haemorrhage management using MOTIVE treatment bundle, and implementation strategies.

### Statistical analysis

Data analysis was conducted using R (version 4.4.1), presented by country and across countries. Frequencies and percentages were reported for categorical outcomes, and medians and IQR for continuous outcomes. To evaluate statistical differences between countries, χ^2^, Fisher, and ANOVA tests were performed. p values less than 5% were considered significant. Fidelity to postpartum haemorrhage early detection was calculated using two measures: proportion of pregnant women and people with a calibrated drape applied, and whether clinical assessments were completed every 15 min for the first hour after birth.

### Role of the funding source

The funders of the study had no role in study design, data collection, data analysis, data interpretation, or writing of the report.

## Results

Between Between June 1, 2022, and Jan 31, 2023, 2578 births were observed, 295 pregnant women and people had postpartum haemorrhage, 47 qualitative interviews were done (32 at intervention hospitals and 15 at control hospitals), and 889 surveys were completed (461 at intervention hospitals and 428 at control hospitals; appendix 2 pp 23–25) in Kenya, Nigeria, South Africa, and Tanzania.

Fidelity of calibrated drape use was high across countries (Table 2, Table 3, Table 4; appendix 2 pp 26–27, 30–31). Observations showed drape application for all pregnant women and people, and surveyed health workers reported always/often using the drape. Staff midwives typically placed the drape (1925 [74·7%] of 2578 times). Research midwives also placed the drape, especially in Nigeria (449 [48·5%] of 925) and Tanzania (128 [19·7%] of 651). Observations and qualitative interviews showed correct adoption of the drape for most pregnant women and people, appropriate materials put in the funnel, and correct duration of use.

**Table 2:** Calibrated drape, bundle, and implementation strategies as measured by intervention survey data

		**Kenya (n=128)**	**Nigeria (n=181)**	**South Africa (n=62)**	**Tanzania (n=90)**	**Total (n=461)**	**p value**	**Implementation outcome**
Drape used for postpartum haemorrhage detection		128 (100%)	179 (98·9%)	62 (100%)	90 (100%)	459 (99·6%)	0·78	High fidelity
Frequency of drape use*								
	Always/often	127 (99·2%)	174 (97·2%)	61 (98·4%)	89 (98·9%)	451 (98·3%)	0·26	High fidelity and adoption
	Sometimes	1 (0·8%)	5 (2·8%)	1 (1·6%)	0	7 (1·5%)	..	..
	Rarely/never	0	0	0	1 (1·1%)	1 (0·2%)	..	..
Reason for not using drape								
	Inconsistent supply	5 (3·9%)	20 (11·0%)	1 (1·6%)	4 (4·4%)	30 (6·5%)	0·014	High feasibility
	Unavailability	2 (1·6%)	12 (6·6%)	3 (4·8%)	1 (1·1%)	18 (3·9%)	0·059	..
	Drape not easily located	2 (1·6%)	9 (5·0%)	3 (4·8%)	2 (2·2%)	16 (3·5%)	0·33	..
	Prefer not to use the drape	0	4 (2·2%)	0	2 (2·2%)	6 (1·3%)	0·22	..
Time when drape was applied								
	Before birth	9 (7·0%)	24 (13·3%)	7 (11·3%)	0	40 (8·7%)	0·0087	High fidelity
	After baby's delivery but before placenta	107 (83·6%)	145 (80·1%)	52 (83·9%)	86 (95·6%)	390 (84·6%)	..	..
	After placenta is delivered	12 (9·4%)	12 (6·6%)	3 (4·8%)	4 (4·4%)	31 (6·7%)	..	..
Approximate duration of drape use								
	About 30 min	12 (9·4%)	18 (9·9%)	2 (3·2%)	5 (5·6%)	37 (8·0%)	0·27	High fidelity
	About 60 min or more	116 (90·6%)	163 (90·1%)	60 (96·8%)	85 (94·4%)	424 (92·0%)	..	..
Belief that calibrated drape is…								
	Very effective	126 (98·4%)	171 (94·5%)	58 (93·5%)	86 (95·6%)	441 (95·7%)	0·012	High acceptability
	Somewhat effective	2 (1·6%)	10 (5·5%)	4 (6·5%)	1 (1·1%)	17 (3·7%)	..	..
	Not effective	0	0	0	3 (3·3%)	3 (0·7%)	..	..
Methods used to manage postpartum haemorrhage								
	Uterine massage	121 (94·5%)	169 (93·4%)	62 (100%)	88 (97·8%)	440 (95·4%)	0·093	High fidelity
	Monitor blood pressure & pulse rate	116 (90·6%)	163 (90·1%)	56 (90·3%)	80 (88·9%)	415 (90·0%)	0·98	..
	Perform examination of cause of bleeding	118 (92·2%)	170 (93·9%)	57 (91·9%)	83 (92·2%)	428 (92·8%)	0·91	..
	Administer intravenous fluids	113 (88·3%)	160 (88·4%)	56 (90·3%)	81 (90·0%)	410 (88·9%)	0·95	..
	Administer tranexamic acid	116 (90·6%)	156 (86·2%)	56 (90·3%)	79 (87·8%)	407 (88·3%)	0·63	..
	Administer uterotonics	115 (89·8%)	166 (91·7%)	53 (85·5%)	82 (91·1%)	416 (90·2%)	0·54	..
Use all MOTIVE bundle components when managing postpartum haemorrhage								
	Always/usually	123 (96·1%)	167 (92·3%)	61 (98·4%)	86 (95·6%)	437 (94·8%)	0·17	High fidelity
	Sometimes	5 (3·9%)	13 (7·2%)	1 (1·6%)	2 (2·2%)	21 (4·6%)	..	..
	Rarely/never	0	1 (0·6%)	0	2 (2·2%)	3 (0·7%)	..	..
Delivery of MOTIVE bundle components								
	Give components at once or in quick succession	118 (92·2%)	150 (82·9%)	52 (83·9%)	81 (90·0%)	401 (87·0%)	0·071	High fidelity
	Give a component, wait to see if it works	10 (7·8%)	31 (17·1%)	10 (16·1%)	9 (10·0%)	60 (13·0%)	..	..
Availability of uterotonics								
	Often/always	126 (98·4%)	175 (96·7%)	62 (100%)	88 (97·8%)	451 (97·8%)	0·60	High feasibility
	Sometimes	2 (1·6%)	6 (3·3%)	0	2 (2·2%)	10 (2·2%)	..	..
	Never/rarely	0	0	0	0	0	..	..
Availability of tranexamic acid								
	Often/always	111 (86·7%)	162 (89·5%)	62 (100%)	79 (87·8%)	414 (89·8%)	0·033	High feasibility
	Sometimes	16 (12·5%)	16 (8·8%)	0	10 (11·1%)	42 (9·1%)	..	..
	Never/rarely	1 (0·8%)	3 (1·7%)	0	1 (1·1%)	5 (1·1%)	..	..
Availability of intravenous fluids								
	Often/always	123 (96·1%)	176 (97·2%)	62 (100%)	89 (98·9%)	450 (97·6%)	0·41	High feasibility
	Sometimes	5 (3·9%)	5 (2·8%)	0	1 (1·1%)	11 (2·4%)	..	..
	Never/rarely	0	0	0	0	0		
Have postpartum haemorrhage trolley, carry-case or box								
	Yes—in all wards	59 (46·1%)	104 (57·5%)	42 (67·7%)	53 (58·9%)	258 (56·0%)	0·064	Mixed feasibility
	Yes—in some wards	67 (52·3%)	75 (41·4%)	19 (30·6%)	37 (41·1%)	198 (43·0%)	..	..
	None	2 (1·6%)	2 (1·1%)	1 (1·6%)	0	5 (1·1%)	..	..
Use postpartum haemorrhage trolley, carry-case or box†								
	Always/often	119 (94·4%)	165 (92·2%)	50 (82·0%)	89 (98·9%)	423 (92·8%)	0·0006	High fidelity
	Sometimes	7 (5·6%)	11 (6·1%)	5 (8·2%)	1 (1·1%)	24 (5·3%)	..	..
	Rarely/never	0	3 (1·7%)	6 (9·8%)	0	9 (2·0%)	..	..
Received feedback or information on postpartum haemorrhage		121 (94·5%)	158 (87·3%)	56 (90·3%)	89 (98·9%)	424 (92·0%)	0·0057	High fidelity
Received E-MOTIVE training		125 (97·7%)	175 (96·7%)	60 (96·8%)	88 (97·8%)	448 (97·2%)	0·94	High fidelity
Attended practice drill sessions‡		112 (90·3%)	134 (79·3%)	51 (87·9%)	76 (88·4%)	373 (85·4%)	0·039	Mixed fidelity
	0	12 (9·7%)	35 (20·7%)	7 (12·1%)	10 (11·6%)	64 (14·6%)	<0·0001	..
	1 to 2	29 (23·4%)	79 (46·7%)	31 (53·4%)	29 (33·7%)	168 (38·4%)	..	..
	3 to 4	21 (16·9%)	34 (20·1%)	9 (15·5%)	21 (24·4%)	85 (19·5%)	..	..
	All 5	62 (50·0%)	21 (12·4%)	11 (19·0%)	26 (30·2%)	120 (27·5%)	..	..
Being an E-MOTIVE champion was part of participant's role§		73 (58·4%)	67 (38·3%)	17 (28·3%)	65 (73·9%)	222 (49·6%)	<0·0001	..
Aware of the E-MOTIVE champions at the hospital§		121 (96·8%)	153 (87·4%)	47 (78·3%)	82 (93·2%)	403 (90·0%)	0·0005	Moderate fidelity

Data are n (%), unless otherwise specified. p values were calculated with Fisher's exact and χ^2^ tests. MOTIVE=uterine massage, oxytocic drugs, tranexamic acid, intravenous fluids, examination and escalation.

* Nigeria n=179 and total n=459.

† Kenya n=126, Nigeria n=179, South Africa n=61, and total n=456.

‡ Kenya n=124, Nigeria n=169, South Africa n=58, Tanzania n=86, and total n=437.

§ Kenya n=125, Nigeria n=175, South Africa n=60, Tanzania n=88, and total n=448.

**Table 3:** Calibrated drape use among women who had vaginal birth as measured by observation data

		**Kenya**	**Nigeria**	**South Africa**	**Tanzania**	**Total**	**p value**	**Implementation outcome**
Applied calibrated drape		..	..	..	..	..	..	High fidelity and adoption
	Staff midwife, student midwife, or nurse	673/734 (91·7%)	465/925 (50·3%)	267/268 (99·6%)	520/651 (79·9%)	1925/2578 (74·7%)	<0·0001	..
	Research midwife	56/734 (7·6%)	449/925 (48·5%)	0/268	128/651 (19·7%)	633/2578 (24·6%)	..	..
	Doctor, medical student, or intern	5/734 (0·7%)	10/925 (1·1%)	1/268 (0·4%)	3/651 (0·5%)	19/2578 (0·7%)	..	..
	Community health extension worker	0/734	1/925 (0·1%)	0/268	0/651	1/2578 (<0·1%)	..	..
Drape tied and secured to woman's waist		728/734 (99·2%)	795/925 (85·9%)	237/268 (88·4%)	651/651 (100%)	2411/2578 (93·5%)	<0·0001	High fidelity
Only blood and blood-soaked gauze or pads put in funnel		734/734 (100%)	923/925 (99·8%)	268/268 (100%)	651/651 (100%)	2576/2578 (99·9%)	0·47	High fidelity
Time of birth to drape funnel opening (min)		2 (1–2)	1 (1–1)	3 (2–5)	2 (2–3)	2 (1–3)	<0·0001	High fidelity
Median time drape was applied (min)		68 (62–77)	64 (60–74)	63 (60–72)	59 (58–61)	63 (59–72)	<0·0001	High fidelity
Clinical assessments from birth to 1-hour postpartum*		..	..	..	..	..	..	Mixed fidelity
	0	1/734 (0·1%)	133/925 (14·4%)	1/268 (0·4%)	176/651 (27·0%)	311/2578 (12·1%)	<0·0001	..
	1	733/734 (99·9%)	792/925 (85·6%)	267/268 (99·6%)	475/651 (73·0%)	2267/2578 (87·9%)	<0·0001	..
	2	669/734 (91·1%)	578/925 (62·5%)	238/268 (88·8%)	310/651 (47·6%)	1795/2578 (69·6%)	<0·0001	..
	3	571/734 (77·8%)	480/925 (51·9%)	215/268 (80·2%)	137/651 (21·0%)	1403/2578 (54·4%)	<0·0001	..
	4	297/734 (40·5%)	359/925 (38·8%)	145/268 (54·1%)	86/651 (13·2%)	887/2578 (34·4%)	<0·0001	..
1st clinical assessment: completed by		..	..	..	..	..	..	
	Staff midwife, student midwife, or nurse	585/733 (79·8%)	95/792 (12·0%)	167/266 (62·8%)	308/475 (64·8%)	1155/2266 (51·0%)	<0·0001	Mixed fidelity and adoption
	Research midwife	139/733 (19·0%)	697/792 (88·0%)	0/266	165/475 (34·7%)	1001/2266 (44·2%)	..	..
	Doctor, medical student, or intern	9/733 (1·2%)	0/792	14/266 (5·3%)	2/475 (0·4%)	25/2266 (1·1%)	..	..
	Enrolled nurse assistant	0/733	0/792	85/266 (32·0%)	0/475	85/2266 (3·8%)	..	..
1st clinical assessment: calibrated drape measurement lines checked		727/733 (99·2%)	779/792 (98·4%)	267/267 (100%)	443/475 (93·3%)	2216/2267 (97·8%)	<0·0001	High fidelity
1st clinical assessment: where was drape funnel lying when reading calibration lines		..	..	..	..	..	..	Moderate fidelity
	Hanging over edge of bed	404/727 (55·6%)	603/779 (77·4%)	254/267 (95·1%)	428/443 (96·6%)	1689/2216 (76·2%)	<0·0001	..
	Flat on bed	323/727 (44·4%)	176/779 (22·6%)	13/267 (4·9%)	15/443 (3·4%)	527/2216 (23·8%)	..	..
1st clinical assessment: if flat on bed, how were calibrated drape lines read		..	..	..	..	..	..	High fidelity
	Moved to edge of bed	314/323 (97·2%)	27/176 (15·3%)	4/13 (30·8%)	15/15 (100%)	360/527 (68·3%)	<0·0001	..
	Visualised flat on bed	8/323 (2·5%)	25/176 (14·2%)	8/13 (61·5%)	0/15	41/527 (7·8%)	..	..
	Lifted to eye level	1/323 (0·3%)	124/176 (70·5%)	1/13 (7·7%)	0/15	126/527 (23·9%)	..	..
1st clinical assessment: calibrated drape measurement lines documented		732/733 (99·9%)	755/792 (95·3%)	265/267 (99·3%)	410/475 (86·3%)	2162/2267 (95·4%)	<0·0001	High fidelity

Data are n (%) or median (IQR), unless otherwise specified. These observational data were only collected in intervention sites. p values were calculated with Fisher's exact, χ^2^, or ANOVA tests.

* Analysis of clinical assessments 2–4 is available in appendix 2 (pp 28–29).

**Table 4:** Summary of key themes from qualitative interviews

		**Exemplar quotes**
**Early Detection of PPH**		
Fidelity, adoption, and adaptation		
	Consistent adoption of calibrated drape for early detection of PPH	“We always use a drape, with each and every woman.” (Nurse, #26, South Africa)
	Calibrated drape correctly used as intended	“We use a drape to every woman with normal delivery, soon after delivery we put a drape before delivering the placenta and we manage for 1 hour to record drape weight at every 15 minutes we read. But after 1 hour if the mother has no other problems and there is no progress of bleeding, we remove it but in case of PPH we might continue for the 2 hours and above until when the mother is stable.” (Midwife, #23, Tanzania)
	Vital signs consistently taken	“We always take vital signs before the woman's delivered and after she deliver.” (Midwife, #12, Nigeria)
Acceptability		
	Earlier and more accurate detection of PPH using the calibrated drape (COM-B: reflective motivation and psychological capability)	“Now because we were using the calibrated drapes, it's more accurate. And…we're more likely to detect PPH earlier than when you were just doing visual…visual inspection used to probably underestimate the loss of blood.” (Doctor, #13, Kenya)
	Ease of use and cleanliness (COM-B: reflective motivation and psychological capability)	“Because it's less messy actually, unlike before, taking deliveries on the clean bed and everything. It tends to get messy. But with drape it collects everything inside and…it makes it neat actually.” (Midwife, #02, Nigeria)
	Vital signs as valued prompts to action (COM-B: psychological capability)	“…why would my patient's blood pressure suddenly drop? Why would the pulse be high? So, you need to go and investigate physically and see…are they responding to the treatment that we're giving them.” (Midwife, #28, South Africa)
	Acceptability of the drape by women giving birth (COM-B: social opportunity)	“We tell them the purpose of it. It's because we want to measure your blood loss…sometimes for some it's quite uncomfortable, but…I've never witnessed a patient that actually refused it. Because we properly explained to them before applying it.” (Midwife, #02, Nigeria); “Putting them in the drape is quite a challenge. Sometimes they refuse so you have to coax them or force them.” (Doctor, #14, Kenya)
Feasibility		
	Availability of supplies (COM-B: physical opportunity)	“Yes, we have [drapes]. So, we never went out of stock.” (Midwife, #26, South Africa)
	Barriers to taking vital signs (COM-B: physical and social opportunity, and reflective motivation)	“It's a little bit challenging when babies need to be breastfed or when we have a perineal tears that need to be sutured. So, there's constantly two or three nurses busy, one will do the observations, one will attend to the perineal tears. So, I think most of our challenges when a patient is on the drape, and she needs to be sutured. So that's quite an uncomfortable position for her to be in.” (Midwife, #28, South Africa)
**Management of PPH**		
Fidelity, adoption, and adaptation		
	Variation in when the MOTIVE bundle is triggered to manage a PPH	“When blood loss reached 300 mL, we check the patient vitals, then we trigger the E-MOTIVE immediately.” (Midwife, #01, Nigeria); “When blood loss reached 500 mL, that's when PPH is diagnosed. So, still we trigger the bundle.” (Midwife, #07, Nigeria)
	Adherence to MOTIVE bundle for management of PPH	“As we take interventions, the other members start taking the vitals…some…they implement the E-MOTIVE bundle by administering tranexamic acid, IV fluids, with…saline and 10 international unit, oxytocin…flow very fast, and the other one take part in uterine massage.” (Midwife, #10, Kenya)
	Deviations from, and additions to, the MOTIVE bundle	“I would not give her tranexamic acid…everybody come up administer the uterotonics…by the time I empty the bladder, massage the uterus, the bleeding may stop. So, some are more often than others.” (Midwife, #8, Nigeria)
Acceptability		
	Improved outcomes for women (COM-B: reflective motivation)	“It has reduced the maternal mortality and morbidity. So, most of the times we have been able to capture [blood loss] before it becomes detrimental to the mother's health.” (Doctor, #12, Kenya)
	Empowerment of nurses and midwives (COM-B: reflective motivation and social opportunity)	“Our nursing staff is empowered to start treatment and not wait for it. Because it before it's too late to actually start treatment.” (Doctor, #25, South Africa)
	Acceptance as part of clinical role and responsibilities (COM-B: social opportunity)	“MOTIVE bundle is actually supposed to be research but any research that is impacting the outcome of patients…yeah, it's part of my clinical role.” (Doctor, #08, Nigeria)
	Impact on workload (COM-B: reflective motivation and physical opportunity)	“There was like a lot of resistance because it was like an added workload on the staff. And I'm sure everybody knows not the hospital is like chaotic.” (Midwife, #30, South Africa)
	Initial reluctance and adapting to something new (COM-B: reflective motivation)	“At first when we were not familiar it's kind of…you can easily forget it, but for now when we are used to it so it's no more difficult.” (Midwife, #05, Nigeria)
	Self-efficacy and ease of delivering the bundle (COM-B: reflective motivation and psychological capability)	“I'm confident because every so far, the period I've worked in labour ward as that MOTIVE bundle been key guide have seen it working. It has never failed. It has never failed. So, you have that confidence in it.” (Midwife, #16, Kenya)
	Easy to remember and becoming automatic (COM-B: automatic motivation and psychological capability)	“Because in the beginning, you used to forget but it is now stuck in our brains. Okay. I don't think we ever forget.” (Midwife, #27, Nigeria)
	Disciplinary action for not adhering to bundle (COM-B: social opportunity)	“There are disciplinary actions it might happen, though here is very few, maybe if it happens the mother has PPH and management was not provided effectively, we normally [have] meetings. We have ward meeting which involves MOI so we correct each other, that in this scenario mother would have ended up to 400 mL but you let her reaches 1000 mL and give her complications or why didn't you deliver this and that in that case.” (Midwife, #23, Tanzania)
	Negative emotions (COM-B: automatic motivation)	“This bundle makes me feel confident, because I know that when [I] use this...the mother will not have any problems. I will be monitoring there, I check. I am not afraid at all; I am doing it without any fear.” (Midwife, #19, Tanzania)
Feasibility		
	Good understanding of bundled approach to PPH management (COM-B: psychological capability)	“The MOTIVE bundle is a set of medicine and procedures put in place to help a mother who has delivered and having excessive bleeding. This bundle consists of the following including E-MOTIVE trolley, it's where the whole bundle is carried, there is the drape that measure blood, there are the principles for early determination. To administer oxytocin, medicine that helps the uterus to contract and prevent bleeding, there is tranexamic acid that helps in blood clotting, there is misoprostol for uterus contraction, there are the fluids to help restore the patients vitals, then there is the monitoring charts that you fill to help in the monitoring of the patient to determine the next step to take, we also have the VSA machine that helps determine the mother's progression blood pumping, blood and pulse pressure and it also set us with time frame to attend the mother and past the time frame I should take a different step. All set of principles and the medication and the whole trolley setup, the bundle is complete and it gives us the time frame.” (Doctor, #17, Tanzania)
	Staff shortages and workforce challenges (COM-B: social opportunity)	“We have very short staff here.” (Midwife, #27, Nigeria); “That is a challenge, staffs are not adequate in labour ward sometimes you might be alone in the shift.” (Midwife, #23, Tanzania)
	Need for multiple staff to deliver the bundle (COM-B: social opportunity and reflective motivation)	“I have implemented the bundle alone. And it was a bit tricky because have to be so fast to implement.” (Midwife, #11, Kenya)
	Improved team working and communication (COM-B: social opportunity)	“Communication has changed greatly…for now, shouting has been synchronised, once you shout the others understand what is happening why is happening, so [they] respond quickly as compared to the past before someone was aware not trained.” (Midwife, #17, Tanzania)
	Peer support and encouragement (COM-B: social opportunity)	“Your colleagues, like, motivate you and they cheer you on, like, you know, you did a good job, the patient is stable, well done, you know, things like that actually boosts our teams.” (Midwife, #30, South Africa)
	Involvement of research midwife (COM-B: social opportunity)	“We have a shortage of staffing. So, they work together with us as we can implement the bundle as fast as possible and trying to call others and also the other team member.” (Midwife, #11, Kenya)
	Availability of drugs (COM-B: physical opportunity)	“Oxytocin we have but tranexamic acid…most likely to be out of stock…sometimes we didn't have even normal saline.” (midwife, #15, Kenya); “Especially the oxytocin sometimes, because the quality of it is not as like the branded one that we used to have from the MOTIVE bundle.” (Midwife, #03, Nigeria)
	Bed shortages (COM-B: physical opportunity)	“It's so busy, our turnover is so high that sometimes, you know, there's no beds. Yeah, that 1 hour, the patients on the bench, whereas we needed the bed for someone else.” (Midwife, #31, South Africa)

Full results tables for the qualitative data, with exemplar quotes from each country for each theme, are available in appendix 2 (pp 28–39). COM-B=model of behaviour change. IV=intravenous. MOI=mechanism of injury. MOTIVE=uterine massage, oxytocic drugs, tranexamic acid, intravenous fluids, examination and escalation. PPH=postpartum haemorrhage. #=ID number. VSA=vital signs alert.

Health worker acceptability of the drape was high across countries: 441 (95·7%) of 461 survey respondents rated the drape as very effective, and 445 (96·5%) liked using it (table 2, figure). Qualitative interviews showed the drape enabled earlier and more accurate postpartum haemorrhage detection and was easy to use; a staff midwife in South Africa stated that “it is a very good tool” and enables diagnosis “early enough to act quickly” (table 4). The drape also improved cleanliness; a staff midwife in Nigeria explained that it was “less messy as it collects everything inside”. However, interview participants reported that the drape was not always acceptable to pregnant women and people, who might find it “uncomfortable”, or that some “removed the drape herself before the [required] hour was finished” (reported by a staff midwife in Tanzania). Women were more accepting of the drape when staff clearly “explained why the drape was put on” (reported by a staff midwife in South Africa).

**Figure:**
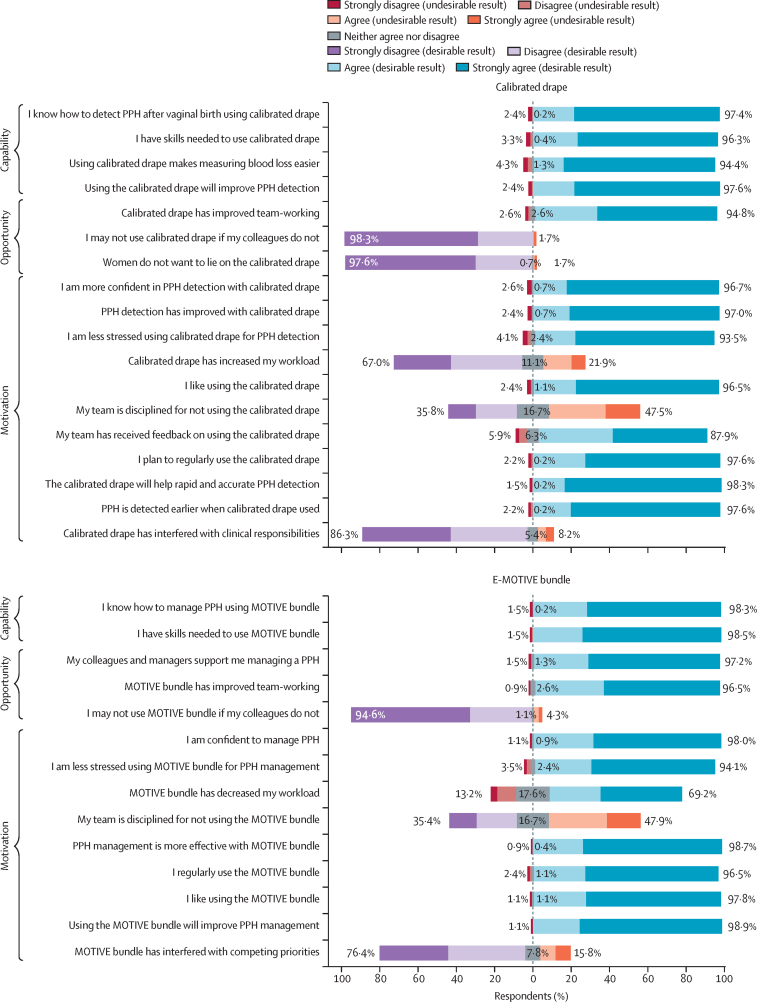
Acceptability and feasibility of calibrated drape and MOTIVE bundle as measured by intervention survey Frequency data from the cross-sectional survey (n=461) conducted in intervention sites, about health workers’ acceptability and feasibility opinions of the calibrated blood-collection drape and MOTIVE bundle. Survey questions are organised according to the Capability, Opportunity, and Motivation COM-B, which helps to assess the extent to which health workers had the capability, opportunity, and motivation to implement the E-MOTIVE intervention components. Colours are used depending on the context of the question. Blue and purple indicate desirable results and red and orange indicate undesirable results. COM-B=model of behaviour change. MOTIVE=uterine massage, oxytocic drugs, tranexamic acid, intravenous fluids, examination and escalation. PPH=postpartum haemorrhage.

Surveys and qualitative interviews showed high feasibility of the drape (figure, table 4), with some challenges as introducing the drape increased workload, teams were disciplined by clinical supervisors for not using the drape, and it interfered with other clinical responsibilities. There were no barriers reported about drape availability; interview participants reported “never having a shortage of drapes” (reported by a staff midwife in Tanzania).

Observations showed high fidelity to health workers conducting the first postpartum clinical assessments (ie, 15 min after birth) in South Africa (267 [99·6%] of 268) and Kenya (733 [99·9%] of 734) and moderate fidelity in Nigeria (792 [85·6%] of 925) and Tanzania (475 [73·0%] of 651; table 3). Clinical assessments were typically done by hospital clinical staff in South Africa, compared with a mix of staff midwives (Nigeria 95 [12·0%] of 925; Kenya 585 [79·8%] of 734; and Tanzania 308 [64·8%] of 651) and research midwives in other countries (Nigeria 697 [88·0%] of 925; Kenya 139 [19·0%] of 734; and Tanzania 165 [34·7%] of 651). Observations suggest fidelity drift in clinical assessments, as all pregnant women and people were intended to have clinical assessments every 15 min after birth for the first hour after birth. Observations showed that 2267 (87·9%) of 2578 pregnant women and people had the first clinical assessment, 1795 (69·6%) had the second assessment, 1403 (54·4%) had the third assessment, and 887 (34·4%) had the fourth assessment, with substantial differences between countries (appendix 2 pp 28–29). This could be explained by barriers reported in the qualitative interviews such as faulty equipment or needing to share equipment (“BP [blood pressure] machine has got no batteries or not been charged”), high workloads, and being “too busy dealing with complications”, breastfeeding support, and suturing tears (table 4).

Clinical assessments were generally acceptable to health workers. Interview participants viewed them as valued prompts to action: clinical assessments were considered “cardinal indicators, so if any change happens, it makes you think maybe the patient has postpartum haemorrhage…you need to intervene”.

MOTIVE treatment bundle fidelity was high for the 295 observed pregnant women and people diagnosed with postpartum haemorrhage (Table 4, Table 5; appendix 2 pp 32–35), with all MOTIVE bundle components administered for most pregnant women and people (286 [96·9%]). Surveys showed high self-reported fidelity to administering all bundle components (437 [94·8%] of 461). Qualitative interviews suggested some variation in timing of postpartum haemorrhage diagnosis and triggering of MOTIVE bundle: 500 mL blood loss or 300 mL along with other worrying signs (table 4).

**Table 5:** MOTIVE treatment bundle among women with diagnosed PPH as measured by the intervention observation data

		**Kenya (n=75)**	**Nigeria (n=141)**	**South Africa (n=57)**	**Tanzania (n=22)**	**Total (n=295)**	**p value**	**Implementation outcome**
Who diagnosed PPH								
	Staff midwife, student midwife, or nurse	68 (90·7%)	61 (43·3%)	54 (94·7%)	11 (50·0%)	194 (65·8%)	<0·0001	High fidelity and adoption
	Research or implementation midwife	2 (2·7%)	68 (48·2%)	2 (3·5%)	11 (50·0%)	83 (28·1%)	..	..
	Doctor, medical student, or intern	5 (6·7%)	12 (8·5%)	1 (1·8%)	0	18 (6·1%)	..	..
Reason for triggering MOTIVE								
	≥300 mL blood loss and abnormal clinical signs	49 (65·3%)	66 (46·8%)	10 (17·5%)	15 (68·2%)	140 (47·5%)	<0·0001	High fidelity
	≥500 mL blood loss	14 (18·7%)	55 (39·0%)	46 (80·7%)	2 (9·1%)	117 (39·7%)	..	..
	Clinical judgment	12 (16·0%)	20 (14·2%)	1 (1·8%)	5 (22·7%)	38 (12·9%)	..	..
PPH trolley or carry-case brought bedside		69 (92·0%)	40 (28·4%)	57 (100%)	20 (90·9%)	186 (63·1%)	<0·0001	Mixed fidelity
PPH trolley or carry-case brought bedside by*								
	Staff midwife, student midwife, or nurse	33 (47·8%)	20 (50·0%)	34 (59·6%)	16 (80·0%)	103 (55·4%)	<0·0001	High fidelity and adoption
	Research or implementation midwife	36 (52·2%)	19 (47·5%)	2 (3·5%)	4 (20·0%)	61 (32·8%)	..	..
	Doctor, medical student, or intern	0	0	6 (10·5%)	0	6 (3·2%)	..	..
	Enrolled nurse assistant or health assistant	0	1 (2·5%)	15 (26·3%)	0	16 (8·6%)	..	..
Uterine massage performed		75 (100%)	140 (99·3%)	56 (98·2%)	22 (100%)	293 (99·3%)	0·53	High fidelity
Uterine massage performed by†								
	Staff midwife, student midwife, or nurse	69 (92·0%)	83 (59·3%)	54 (96·4%)	16 (72·7%)	222 (75·8%)	<0·0001	Mixed fidelity and adoption
	Research or implementation midwife	4 (5·3%)	46 (32·9%)	1 (1·8%)	6 (27·3%)	57 (19·5%)	..	..
	Doctor, medical student, or intern	2 (2·7%)	9 (6·4%)	1 (1·8%)	0	12 (4·1%)	..	..
	Other‡	0	2 (1·4%)	0	0	2 (0·7%)	..	..
Oxytocin administered for first-line PPH treatment§		75 (100%)	141 (100%)	57 (100%)	22 (100%)	295 (100%)	NA	High fidelity
Oxytocin administration started by								
	Staff midwife, student midwife, or nurse	47 (62·7%)	48 (34·0%)	53 (93·0%)	16 (72·7%)	164 (55·6%)	<0·0001	High fidelity and adoption
	Research or implementation midwife	25 (33·3%)	87 (61·7%)	2 (3·5%)	6 (27·3%)	120 (40·7%)	..	..
	Doctor, medical student, or intern	3 (4·0%)	6 (4·3%)	2 (3·5%)	0	11 (3·7%)	..	..
Tranexamic acid administered for PPH treatment		74 (98·7%)	138 (97·9%)	57 (100%)	22 (100%)	291 (98·6%)	0·86	High fidelity
Tranexamic acid administration started by¶								
	Staff midwife, student midwife, or nurse	41 (55·4%)	36 (26·1%)	53 (93·0%)	16 (72·7%)	146 (50·2%)	<0·0001	High fidelity and adoption
	Research or implementation midwife	26 (35·1%)	90 (65·2%)	2 (3·5%)	5 (22·7%)	123 (42·3%)	..	..
	Doctor, medical student, or intern	7 (9·5%)	12 (8·7%)	2 (3·5%)	1 (4·5%)	22 (7·6%)	..	..
Examination of genital tract and placenta		75 (100%)	138 (97·9%)	57 (100%)	22 (100%)	292 (99·0%)	0·55	High fidelity
Examination of genital tract and placenta done by‖								
	Staff midwife, student midwife, or nurse	57 (76·0%)	85 (61·6%)	44 (77·2%)	9 (40·9%)	195 (66·8%)	<0·0001	High fidelity and adoption
	Research or implementation midwife	2 (2·7%)	27 (19·6%)	1 (1·8%)	8 (36·4%)	38 (13·0%)	..	..
	Doctor, medical student, or intern	16 (21·3%)	26 (18·8%)	11 (19·3%)	5 (22·7%)	58 (19·9%)	..	..
	Midwife and doctor**	0	0	1 (1·8%)	0	1 (0·3%)	..	..
Placenta examined prior to discarding		73 (97·3%)	32 (22·7%)	56 (98·2%)	16 (72·7%)	177 (60·0%)	<0·0001	Mixed fidelity
Management of PPH escalated		11 (14·7%)	9 (6·4%)	6 (10·5%)	3 (13·6%)	29 (9·8%)	0·23	..
Who escalated management of PPH††								
	Staff midwife, student midwife, or nurse	8 (72·7%)	5 (55·6%)	3 (50·0%)	0	16 (55·2%)	0·16	High fidelity and adoption
	Research or implementation midwife	0	2 (22·2%)	1 (16·7%)	0	3 (10·3%)	..	..
	Doctor, medical student, or intern	3 (27·3%)	2 (22·2%)	2 (33·3%)	3 (100%)	10 (34·5%)	..	..
Median time from clinical PPH diagnosis to last treatment bundle component initiated		15 (9–19)	10 (5–15)	20 (15–25)	10 (5–15)	13 (6–18)	<0·0001	Mixed fidelity
All treatment bundle components administered		74 (98·7%)	134 (95·0%)	56 (98·2%)	22 (100%)	286 (96·9%)	0·48	High fidelity
Time from PPH diagnosis until initiation of last treatment for women with all treatment bundle components administered‡‡								
	≤15 min	40 (54·1%)	112 (83·6%)	22 (39·3%)	17 (77·3%)	191 (66·8%)	<0·0001	Mixed fidelity
	16–20 min	22 (29·7%)	9 (6·7%)	11 (19·6%)	0	42 (14·7%)	..	..
	21–30 min	10 (13·5%)	11 (8·2%)	15 (26·8%)	2 (9·1%)	38 (13·3%)	..	..
	≥31 min	2 (2·7%)	2 (1·5%)	8 (14·3%)	3 (13·6%)	15 (5·2%)	..	..

Data are n (%) or median (IQR), unless otherwise specified. p values were calculated with Fisher's exact, χ^2^, or ANOVA tests. MOTIVE=uterine massage, oxytocic drugs, tranexamic acid, intravenous fluids, examination and escalation. NA=not applicable. PPH=postpartum haemorrhage.

* Kenya n=69, Nigeria n=40, Tanzania n=20, and total n=186.

† Nigeria n=140, South Africa n=56, and total n=293.

‡ Women self-conducted uterine massage.

§ No statistical test was done due to 100% in all countries.

¶ Kenya n=74, Nigeria n=138, and total n=291.

‖ Nigeria n=138 and total n=292.

** Participant provided the further detail of midwife and doctor after selecting “Other” as their response.

†† Kenya n=11, Nigeria n=9, South Africa n=6, Tanzania n=3, and total n=29.

‡‡ Kenya n=74, Nigeria n=134, South Africa n=56, and total n=286.

There was mixed-fidelity in median timing of postpartum haemorrhage diagnosis to final treatment initiation across countries (Kenya 15 min [IQR 9–19]; Nigeria 10 min [5–15]; South Africa 20 min [15–25]; Tanzania 10 min [5–15]; table 5). Surveys showed high self-reported fidelity to MOTIVE bundle delivery in quick succession (401 [87·0%] of 461). Most interview participants recognised the importance of delivering the bundle in quick succession. However, some reported using a “wait and see” approach, especially for tranexamic acid (table 4).

There was mixed fidelity about who diagnosed postpartum haemorrhage and administered MOTIVE treatment components, with substantial country variation in involvement of research midwives (table 5). Research midwives diagnosed postpartum haemorrhage most commonly in Nigeria (68 [48·2%] of 141) and Tanzania (11 [50·0%] of 22). South Africa showed high fidelity to hospital clinical staff administering MOTIVE components. Research midwives administered MOTIVE components in Nigeria and Tanzania (for all components, ranging from 19·6% to 65·2%), suggesting low to moderate fidelity. In Kenya, there was moderate fidelity of oxytocin administration (research midwife 25 [33·3%] of 75) and tranexamic acid (research midwife 26 [35·1%] of 74), but high fidelity for staff midwives administering uterine massage (research midwife four [5·3%] of 75) and genital tract examination (research midwife two [2·7%] of 75).

Acceptability of the MOTIVE treatment bundle was high across countries and data sources (figure, table 4). Survey participants liked using the bundle, felt confident managing postpartum haemorrhage, and were less stressed; the bundle also decreased workload. Interview participants reported benefits of the MOTIVE bundle (table 4), describing improved outcomes and that “[MOTIVE] had never failed…you have that confidence in it” (midwife in Kenya). Staff midwives took greater responsibility for detecting postpartum haemorrhage and expanded responsibilities for postpartum haemorrhage management, making them feel “more professional”, and “more empowered…rarely needing a doctor”. There were mixed views on workload effects: some stated minimal effect, and others reported that increased responsibilities inevitably increased workload. This potential increase in workload sometimes led to initial reluctance to adopt MOTIVE, while they “adapt to something a bit different and difficult”. Reluctance was typically transient, as familiarity with MOTIVE led to embedding in practice. One staff midwife described MOTIVE as “simplified…in a manner you won't easily forget…after you finish this, you go to that…a routine”. Most participants expressed high self-efficacy and ease regarding bundle use, with participants reporting feeling “confident”, particularly as it involved administering treatments already in practice, albeit not in a bundled approach. Stress and fear that survey and interview participants had previously experienced during postpartum haemorrhage management were reported to be subsided because of MOTIVE and resulting confidence.

Surveys showed some concerns about discipline measures for not using the bundle (221 [47·9%] of 461) and interference with other priorities (73 [15·8%] of 461). Some interview participants reported that they were “punished if [they did] not use [the MOTIVE bundle] and there [was] a case of postpartum haemorrhage” and that some colleagues were “very strict” (table 4). Participants might not necessarily be referring to formal disciplinary actions; rather, peers encouraged adherence to MOTIVE.

Overall, the MOTIVE bundle was feasible to implement. Surveys showed uterotonics, tranexamic acid, and intravenous fluids were almost always available, health workers had appropriate skills, and the bundle improved teamwork (figure, table 2). However, some interview participants described barriers to MOTIVE implementation, including insufficient availability of drugs, supplies, and staffing, and bed shortages (table 4, appendix 2 pp 32–35). Some reported tranexamic acid was “most likely to be out of stock”. Some interview participants highlighted differences between quality-assured drugs supplied within the trial (particularly oxytocin), compared with drugs previously available, and expressed concerns around longer-term sustainability. Many interview participants described how multiple health workers were needed to deliver the MOTIVE bundle and that delivering it on their own in quick succession “is a bit tricky” because “you need a team”. Delivering the bundle was challenging when there was insufficient staffing, with participants across all countries describing labour ward staff shortages, and sometimes “being alone on a shift”. Agency staff (ie, locums) were sometimes hired to address staff shortages, but this presented challenges as locums might not have been trained in MOTIVE. Teamwork and communication were recognised as crucial enablers to implement MOTIVE, with improvements made from collaboratively implementing MOTIVE: “shouting becoming synchronised—once you shout, the others understand what is happening…respond quickly compared to before”. Interview participants also explained the benefits of peer support, encouragement, motivation, and reminders.

Surveys and interviews showed a high self-reported fidelity of health workers receiving initial E-MOTIVE training. Interview participants reported that “everyone has been trained”. Surveys showed that participants felt adequately trained and that training was helpful and improved their understanding of using the drape and bundle (table 2, appendix 2 p 20). Interview participants reported that training improved their confidence, knowledge, and skills, and that they particularly valued the simulation training as it was “very helpful…the more you did the roles, the more you become alert…you remember things better” (appendix 2 pp 36–38). Training reportedly led to improved teamwork and communication, as they now “speak one language”. However, fidelity to surveyed health workers attending all five subsequent practice drill sessions was low, with participants stating that they “just haven't had the time” in the interviews.

Among pregnant women and people with observed postpartum haemorrhage, there was moderate fidelity to bringing the postpartum haemorrhage trolley or carry-case to the bedside (table 5). Interview participants reported that the “trolley was always nearby”, but not necessarily used (appendix 2 pp 36–38). Survey participants reported always/often using the trolley or carry-case, which was recorded as high fidelity (table 2). Interview participants reported mixed fidelity, describing that they were consistently stocked, checking “every morning that all things are there”, but “I have not used it personally”. Acceptability of the postpartum haemorrhage trolley or carry-case was high: survey participants liked it, believed it improved postpartum haemorrhage management, and improved how quickly postpartum haemorrhage response can start (table 2, appendix 2 p 20). Interview participants emphasised the benefits of having everything in one place for postpartum haemorrhage management, “so, it's not us running helter-skelter trying to locate supplies”. Some interview participants reported feasibility challenges, including inconsistent trolley availability when multiple pregnant women and people had postpartum haemorrhage simultaneously (“we have only one trolley…you can't just roll it to one person and ignore the other”), or if the labour ward was across multiple rooms. Sometimes staff midwives reported needing to “ask mothers to purchase [supplies]” for their own care when the trolley stock was low or out.

Most survey participants received feedback on postpartum haemorrhage detection and management (table 2, appendix 2 p 20). However, interview participants described mixed fidelity, with some reporting “there is no feedback” or they “haven't seen the [audit] newsletter”, while others reported discussing audit newsletters in ward meetings or on WhatsApp. Audit and feedback were acceptable; survey participants positively described receiving feedback about postpartum haemorrhage management; feedback helped them to use the MOTIVE bundle, improved teamwork, and identified needed improvements. Interview participants highlighted that feedback was highly motivational to identify improved practices. However, some survey participants were concerned about negative feedback, and interview participants described trying harder when they knew they were being assessed.

There was high acceptability and feasibility of postpartum haemorrhage champions among the 403 (90·0%) of 448 of survey participants who reported postpartum haemorrhage champions being present at their facility, which was recorded as high fidelity. Champions were viewed as helpful and available, improved bundle use, and their support and advice was well received (table 2, appendix 2 p 20). There were mixed opinions about whether champions reduced concerns about bundle use. Interview participants similarly reported that champions were helpful, particularly around prompting and “reminding us to implement the bundle” and acting “as role models for us, to show us that you can achieve this” (appendix 2 pp 36–38). Some reported tension between champions and staff, when champions corrected clinical behaviours of hospital staff.

Across interviews and surveys, there were minor indications of contamination between intervention and control sites (appendix 2 pp 21, 39–41). Some interview participants reported sharing information about E-MOTIVE with others, arguing that postpartum haemorrhage outcomes would improve if everyone used E-MOTIVE. Some reported potential competing external activities, such as the release of new postpartum haemorrhage guidelines, working on quality improvement initiatives, or participation in other maternal health research. Some interview participants in control sites noted that uncalibrated drape weighing for trial outcome measurement sometimes prompted health workers to retrospectively diagnose postpartum haemorrhage (appendix 2 p 39), reflecting a change in practice: before E-MOTIVE, participants relied on visual estimation of blood loss.

## Discussion

The mixed-methods design of this process evaluation allowed for exploration and quantification of nuances across independently observed and self-reported practices, providing analytic breadth and depth. The E-MOTIVE process evaluation showed high fidelity to calibrated drape use and the MOTIVE treatment bundle. Acceptability of the drape was high among health workers; but might be lower for pregnant women and people undergoing vaginal birth. Clinical assessments conducted by health workers at 15 min after birth had moderate to high fidelity, with fidelity drift for assessments intended at 30, 45, and 60 min. Fidelity drift suggests more work is needed to embed clinical assessments in existing work routines, which are crucial for early postpartum haemorrhage detection, diagnosis, and treatment initiation. Training less specialised health workers to conduct clinical assessments might address the health workforce and workload challenges affecting the introduction of these assessments (eg, enrolled nurse assistants’ involvement in South Africa). Almost all pregnant women and people with postpartum haemorrhage had all MOTIVE bundle components delivered (ie, high fidelity), with moderate to high fidelity to the intended timeframe. MOTIVE treatment bundle acceptability was high: health workers felt less stressed, more confident, and liked using it. Based on the trial results, WHO recommends routine quantification of postpartum blood loss (eg, using calibrated drapes) and treatment bundle of uterine massage, oxytocics, tranexamic acid, intravenous fluids, genital tract examination and escalation.^7^

High fidelity, acceptability, and feasibility in this process evaluation support and increase confidence in the interpretation and validity of the promising E-MOTIVE trial results.^4^ However, some findings highlight considerations for implementation, scale-up, and sustainability. First, implementing the calibrated drape and MOTIVE bundle was feasible in the trial, but barriers might exist in practice when supplies and medications are not available. Second, calibrated drape fidelity was high in the trial and was necessary for trial primary outcome assessment.^4^ The involvement of research midwives in applying the calibrated drape for a quarter of observed births suggests potential real-world implementation challenges, but this might be mitigated by health workers’ high acceptability of the drape. Third, research midwives were involved in MOTIVE bundle delivery, particularly in Nigeria and Tanzania. Health workforce challenges might have necessitated research midwives to support during emergencies, due to ethical or moral obligations. Some processes, including calibrated drape placement, were mandated for both clinical reasons (volumetric blood loss assessment) and trial purposes (gravimetric primary outcome blood loss assessment), potentially encouraging research midwives’ involvement. Due to promising trial results, a post-trial implementation pivot is underway for control sites to receive the intervention with reduced resourcing from the trial itself (eg, less support from the trial management team). This pivot will provide more information about real-world scale-up and sustainability in other settings, which also must navigate contextual issues around health workforce, medicines, and supplies.

This study had some weaknesses. Data collection took place from trial months 3–7, to allow intervention embedding; which might have missed early implementation challenges. We did not assess pregnant women's and people's acceptability of the drape, which would provide valuable future insights. There was some confusion about the postpartum haemorrhage champion's role, and audit and feedback did not always reach all relevant health workers. Audit and feedback might need more systematic dissemination, for example by embedding in existing multidisciplinary clinical meetings.

Early postpartum haemorrhage detection coupled with bundled treatment and implementation strategies will save lives. Trial evidence demonstrated the effectiveness of the E-MOTIVE intervention.^4^ This process evaluation shows high fidelity of trial implementation, and the calibrated drape, treatment bundle, and implementation strategies were acceptable and feasible to implement in secondary-level hospitals in four African countries. Use of a theory-informed approach to intervention development^17^, ^18^, ^19^ and robust formative research^5^, ^13^, ^14^ improved our understanding of factors that were likely to influence behaviour change and enabled development of implementation strategies to address barriers, which probably contributed to the positive trial and evaluation results. These results confirmed programme logic about how and why E-MOTIVE was likely to work: early postpartum haemorrhage detection and bundled treatment can improve outcomes when simulation-based, on-site training is facilitated; supplies and equipment are accessible; staff are supported; health worker roles and responsibilities are clear; and there is protected time and agency to deliver effective care.

### Contributors

### Equitable partnership declaration

### Data sharing

The study protocol, study instruments, de-identified survey and observation data, and statistical code underlying the results reported in this Article will be made available after de-identification, upon request to the corresponding author for the 5 years immediately following. A data sharing agreement requires a commitment to using the data only for specified research purposes, to researchers who provide a methodologically sound proposal, to securing the data appropriately, and to destroying the data after a nominated period.

## Declaration of interests

SMi's University (University of California San Francisco) holds the licence for the Trademark name “LifeWrap”. The LifeWrap is the name of one first-aid device used in refractory postpartum haemorrhage, the non-pneumatic anti-shock garment (NASG). The manufacturer of the LifeWrap NASG pays the University of California a royalty for the use of the name. GJH has conceived a reusable device for postpartum blood loss monitoring, the Maternawell Tray, which is marketed by Maternova, a global women's health solutions company who hold the intellectual property. GJH benefits from sales of the device. The E-MOTIVE research project was supported by an investment grant (INV-001393) from the Bill and Melinda Gates Foundation to the University of Birmingham, and via subcontracts to coauthors’ research institutions.

## Supplementary Materials

Swahili translation of the abstract

Supplementary appendix 2

Equitable Partnership Declaration
