# Supplementary material for: Early detection and a treatment bundle strategy for postpartum haemorrhage: a mixed-methods process evaluation
Source: Lancet Glob Health. 2025 Jan 29;13(2):e329–44. doi: 10.1016/S2214-109X(24)00454-6 (PMC11782988; doi:10.1016/S2214-109X(24)00454-6)
Supplement: Swahili translation of the abstract [file mmc1.pdf]

THE LANCET
Global Health

Supplementary appendix 1

This translation in Swahili was submitted by the authors and we reproduce it as supplied. It has not been peer reviewed. *The Lancet*'s editorial processes have only been applied to the original in English, which should serve as reference for this manuscript.

Tafsiri hii katika Swahili iliwasilishwa na waandishi na tunatengeneza tena kama hutolewa. Haijapitiwa. Mchakato wa hariri wa Lancet Global Health umetumika tu kwa asili kwa Kiingereza, ambayo inapaswa kutumika kama kumbukumbu kwa muswada hii.

Supplement to: Bohren MA, Miller S, Mammoliti K-M, et al. Early detection and a treatment bundle strategy for postpartum haemorrhage: a mixed-methods process evaluation. *Lancet Glob Health* 2025; **13:** e329–44.

Utangulizi

Uvujaji wad amu uliopitiliza baada ya kujifungua ni chanzo kikuu cha vifo vitokanavyo na uzazi. Utafiti wa kutathmini afua ya EMOTIVE uliofanywa katika nchi mbalimbali ulionyesha kuwa afua hii ilipunguza madhara yatokanayo na tatizo la uvujaji damu uliopitiliza kwa asilimia sitini (60%). Afua ya E-MOTIVE ilijumuisha ugunduzi wa mapema pale mama anapovuja damu kupitiliza kwa kutumia drepa maalum inayopima wingi wa damu,ikifuatiwa na mhudumu wa afya kutoa kifurushi cha matibabu (masaji ya mfuko wa uzazi, dawa za kukazanisha misuli ya kizazi, maji kwenye mishipa, pamoja na kuchunguza chanzo na kutoa tiba stahiki: ikitolewa na kutekelezwa kwa mbinu na mikakati maalum. Chapisho hili linaripoti tathmini ya utekelezaji wa afua hii uliofanyika nchini Kenya, Nigeria, South Africa, na Tanzania

Mbinu

Mbinu zilizotumika katika utafiti huu ni pamoja na kuangalia jinsi wahudumu wa afya (kwenye vituo vilivyotekeleza afua) wanavyotoa huduma kwa wajawazito wanaojifungua kawaida au wanawake waliopata tatizo la uvujaji wa damu uliopitiliza , pamoja na mahojiano na wahudumu wa afya kutoka vituo vya utekelezaji wa agua na vile vinavyotoa huduma za kawaida. Vituo vilivyotekeleza afua vilipata drapes zilizokua na vipimo (vikiainisha kiwango cha damu uliyovuja) , afua ya EMOTIVE, pamoja na mikakati ya utekelezaji wake. Vituo vilivyoendelea kutoa huduma za kawaida vilipata drepa ambazo hazikuwa na vipimo. Matokeo ya msingi ya utekelezaji ni pamoja na kukubalika, kuchukuliwa, uwezekano wa kutekelezeka kwa uaminifu na usahihi, pamoja na uwezekano wa kuwepo uchafuzi au mchanganyiko wa matumizi ya drepa, afua au mbinu za utekelezaji wake.

Matokeo

Kati ya Juni 1, 2022, na Jan 31, 2023, tulikamilisha uchunguzi wa vizazi 2578, wanawake 295 waliopata tatizo la kuvuja damu kupitiliza, mahojiano na wahudumu wa afya 47, pamoja na wahudumu 889 kujaza dodoso kwa njia ya mtandao. Matumizi ya drepa maalum inayopima wingi wa damu ulifanyika kwa uaminifu na usahihi wa juu (2578 [100%] ya wanawake 2578 waliojifungua kawaida waliwekewa drepa; dodoso 451 [98.3%] kati ya wahudumu 459). Drepa maalum inayopima wingi wa damu zilikubalika kwa kiwango cha juu miongoni mwa wahudumu wa afya, hata hivyo, wahudumu wa afya waliripoti kuwepo kwa vikwazo katika ukubalikaji wa drepa kwa wanawake na wajawazito. Utoaji wa kifurushi cha afua ulifanyika kwa usahihi na uaminifu mkubwa (wanawake 286 kati ya 295 [96·9%]), muda uliotumika kuanzisha matibabu kwa wanawake 295 baada ya kugunduliwa tatizo la uvujaji damu uliopitiliza hadi matibabu ya mwisho ulikua ni chini ya au dakika 15 (kwa wanawake 191 [66 · 8%], na dakika 16-20 kwa wanawake 42 [14 · 7%]). Wahudumu wa utafiti walishiriki katika tathmini za wanawake baada ya kujifungua pamoja na utoaji huduma za afua katika baadhi ya vituo (utekelezaji kwa uaminifu uliochanganyika)

Interpretation [Tafsiri]

Tathmini hii ya mchakato ya utekelezaji inaonyesha viwango vya juu vya kukubalika, uwezekano na kutumika kwa uaminifu katika matumizi ya drape na afua ya E-MOTIVE. Matokeo haya yamepatikana katika nchi zote zilizoshiriki utafiti na kwa kutumia tafiti za njia tofauti. Afua ya E-MOTIVE inapaswa kujumuishwa katika sera za kitaifa, na kwa kuzingatia uwepo wa wahudumu wa afya na upatikanaji wa vifaa tiba na dawa stahiki ndani ya vituo vya afya kwa utekelezaji wa mafanikio.
