# Supplementary material for: Early detection and a treatment bundle strategy for postpartum haemorrhage: a mixed-methods process evaluation
Source: Lancet Glob Health. 2025 Jan 29;13(2):e329–44. doi: 10.1016/S2214-109X(24)00454-6 (PMC11782988; doi:10.1016/S2214-109X(24)00454-6)
Supplement: Supplementary appendix 2 [file mmc2.pdf]

THE LANCET
Global Health

Supplementary appendix 2

This appendix formed part of the original submission and has been peer reviewed. We post it as supplied by the authors.

Supplement to: Bohren MA, Miller S, Mammoliti K-M, et al. Early detection and a treatment bundle strategy for postpartum haemorrhage: a mixed-methods process evaluation. *Lancet Glob Health* 2025; **13:** e329–44.

Table of Contents

Appendix 1. Ethics and permission statement 2

Appendix 2. Details of the E-MOTIVE intervention and implementation strategies 3

Appendix 3. Standards for Reporting Implementation Studies: the StaRI checklist for completion 14

Appendix Figure 1. The E-MOTIVE intervention 18

Appendix Figure 2. E-MOTIVE Research Programme 19

Appendix Figure 3: Intervention survey data: acceptability and feasibility of implementation strategies 20

Appendix Figure 4: Control survey data: PPH detection and management 21

Appendix Table 1. Sociodemographic information. 23

Appendix Table 2: Intervention survey data: sociodemographic characteristics 24

Appendix Table 3: Control survey data: sociodemographic characteristics 25

Appendix Table 4: Intervention survey data: calibrated drape and bundle use 26

Appendix Table 5: Intervention observation data: clinical assessments in the first hour after birth among women with vaginal birth 28

Appendix Table 6: Qualitative themes related to each implementation outcomes for early detection of PPH 30

Appendix Table 7: Qualitative themes related to each implementation outcomes for PPH management 32

Appendix Table 8: Qualitative themes related to the implementation strategies 36

Appendix Table 9: Qualitative themes related to contamination 39

Appendix Table 10: Control survey data: calibrated drape and bundle use (contamination) 40

Appendix Study Instrument 1: Qualitative Interview Guide (*Control Sites*) 42

Appendix Study Instrument 2: Qualitative Interview Guide (*Intervention Sites*) 45

Appendix Study Instrument 3: Survey (*Control Sites)* 50

Appendix Study Instrument 4: Survey (*Intervention Sites*) 65

Appendix Study Instrument 5: Observation guide (*Intervention Sites*) 101

Appendix 1. Ethics and permission statement

This study has received ethics approval and permissions from the following entities:

- **United Kingdom**: University of Birmingham STEM committee (Reference number: ERN_19-1557).
- **Kenya**:
  - University of Nairobi: KNH-UON ERC (P25/01/2020),
  - Pharmacy and Poisons Board PPB/ECCT/20/06/06/2020(116),
  - National Commission for Science, Technology and Innovation Nacosti P/21/8437.
- **Nigeria**: National Health Research Ethics Committee of Nigeria (NHREC) (Reference number: NHREC/01/01/2007)-07/04/2020).
- **South Africa**:
  - Eastern Cape Department of Health (EC_202007_015),
  - University of Cape Town Human Research Ethics Committee (HREC; reference number: 091/2020),
  - Health Province of KwaZulu-Natal (NHRD reference number: KZ_202008_036),
  - University of the Witwatersrand Human Research Ethics Committee-Medical (reference number: M200241).
- **Tanzania**:
  - Muhimbili University of Health and Allied Sciences (MUHAS) (reference number: DA.282./298/01.C/),
  - National Institute for Medical Research (NIMR) (Reference number: NIMR/HQ/R.8a/Vol IX/3501).
- **Australia**: University of Melbourne Medicine and Dentistry Human Ethics Sub-Committee (1956004).
- **World Health Organization**:
  - Review Panel on Research Project Review Panel s (RP2),
  - WHO Ethical Review Research Ethics Review Committee (WHO ERC; reference number: ERC.0003486).

As the E-MOTIVE intervention aimed to change health worker behaviours about PPH detection and management, hospital leadership at each site provided hospital-level permission for the observations, and health workers provided written informed consent before the trial-specific training. Individual-level consent from women for the observations was not sought, as women were not the target of the intervention, were not interacted with for the purposes of observation data collection, and no identifiable information on observed women were collected.

Appendix 2. Details of the E-MOTIVE intervention and implementation strategies

Reproduced with permission from: Gallos I, Devall A, Martin J, et al. Randomized Trial of Early Detection and Treatment of Postpartum Hemorrhage. New England Journal of Medicine 2023; 389(1): 11-21. https://www.nejm.org/doi/full/10.1056/NEJMoa2303966

The E-MOTIVE intervention had two main components: the early detection strategy and bundled treatment for postpartum haemorrhage (PPH). The implementation strategy had four components: the PPH trolleys or carry cases; training and clinical protocols; champions; and audit and feedback. The PPH treatment bundle was designed through a technical consultation led by the World Health Organization and based on its recommendations. The Early PPH detection strategy (the first 'E' in E-MOTIVE) and the Examination and Escalation approach (the last 'E' in E-MOTIVE) were developed by the E-MOTIVE investigator group, led by the University of Birmingham. The implementation strategy was adapted for each country via in-country workshops.

Early detection & trigger criteria

The purpose of the early detection strategy was to aid the detection of PPH as early as possible and to ensure that no PPH was missed. A calibrated blood collection drape was used for collecting all postpartum blood and act as a prompt for health workers to treat PPH when excessive blood was lost. Health workers were provided training on the use of a blood loss monitoring chart to document the blood loss volume against the calibration lines on the drape, the vaginal blood flow, and uterine tone every 15 minutes for at least the first hour after childbirth. Blood pressure and pulse were checked and documented at least once in the 1st hour postpartum.

In-country adaptations

Health workers requested the calibrated drapes to have a yellow warning line at 300 mL and a red action line at 500 mL. They preferred to document the blood loss every 15 minutes for the first hour along with the other observations to help them diagnose PPH. In Nigeria, Kenya and Tanzania, a blood loss reaching the yellow line at 300 mL combined with at least one abnormal clinical sign would trigger the MOTIVE bundle, whereas in South Africa, the 300 mL yellow line would act as a warning to be prepared to treat if bleeding continued, and ≥ 500 mL would trigger the MOTIVE bundle..

Key implementation points

- When birth was imminent, the upper portion of the drape was placed underneath the buttock of the woman and the transparent funnel was folded up to ensure no liquor entered the drape during the birth.
- Cotton straps at the upper end of the drape were used to secure the drape around the woman's waist.
- After the birth, staff administered oxytocin for the third stage management (as per usual practice), and then opened the funnel to hang over the edge of the bed before the placenta was delivered. This allowed all blood to be collected in the drape.
- The drape had graduated calibration lines measured in millilitres (Figure S1). 300 mL was highlighted in yellow (warning sign) to alert providers that action may be required and 500 mL in red (action line) to prompt providers to trigger the MOTIVE bundle for treatment of PPH.
- All blood loss following birth was collected into the drape for a minimum of 1 hour, or up to 2 hours if bleeding continued beyond the first hour. The same drape was used if a woman was transferred to the postnatal ward within these 2 hours.

- Any blood-soaked pads and swabs were placed inside the drape. Only dry pads or swabs that weighed <15g were used in the E-MOTIVE research sites.
- In cases of multiple births, the funnel was rolled out once the last baby was born and after oxytocin was administered for third stage management.

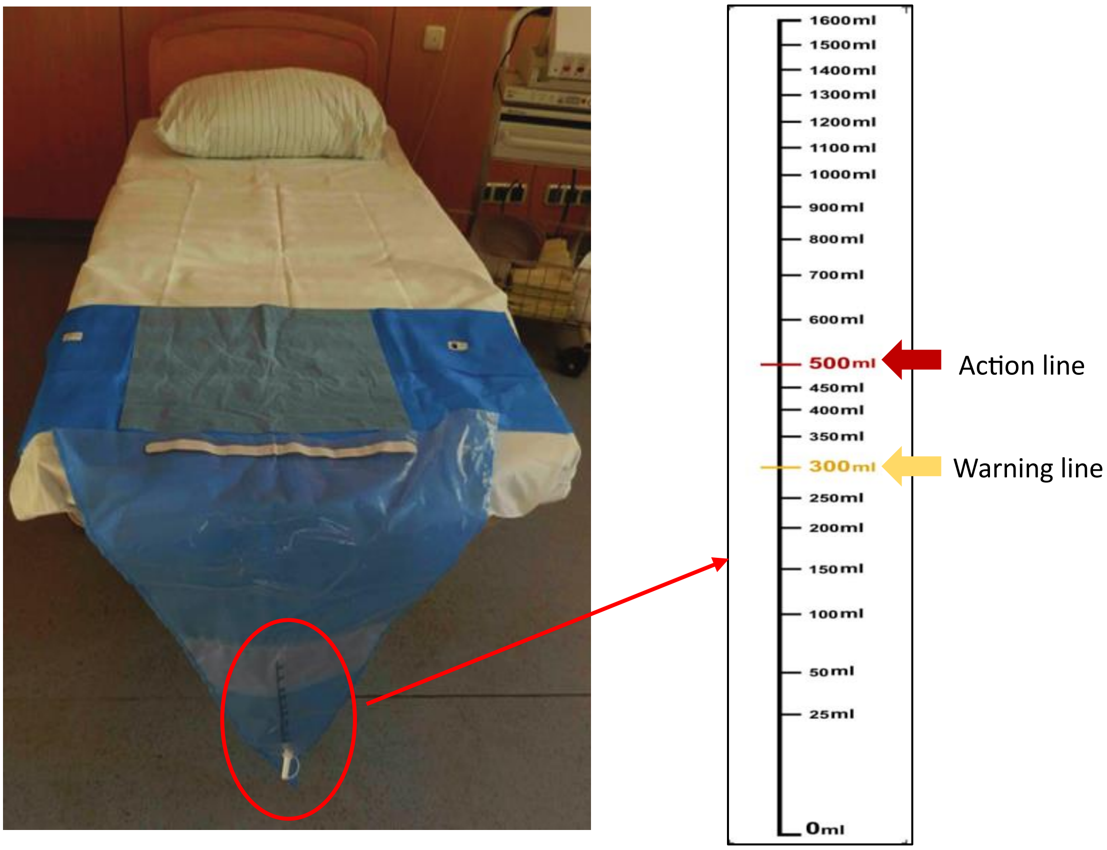

Figure S1. Calibrated blood collection drape showing yellow 'warning' and red 'action' lines

- The cumulative blood loss volume reading was taken using the calibration lines on the drape, with the assessment of uterine tone and vaginal blood flow every 15 minutes. Blood pressure and pulse were completed at least once in the first hour and repeated if they were previously abnormal or the woman had ongoing bleeding. These were documented in a blood loss monitoring chart. Abnormal observations included:
  - *Tachycardia (>100 beats per minute [bpm] or an increase of 20 bpm from baseline),*
  - *Decreasing systolic BP (<100 mmHg or a decrease of 20 mmHg from baseline),*
  - *Soft uterine tone,*
  - *Heavy vaginal blood flow, large clots being expelled, or a constant trickle of blood.*
- MOTIVE was triggered if any of the following three criteria was met:
  - Clinical judgement – heavy vaginal blood loss, large blood clots, a constant trickle, or some other concern on the provider's assessment.
  - Blood loss ≥ 300 mL to <500 mL observed in the drape, plus at least one abnormal clinical sign
  - Blood loss ≥ 500 mL observed in the drape (regardless of other clinical signs).

Bundled treatment for PPH

The purpose of the bundled treatment for PPH was to ensure timely and consistent use of recommended interventions for PPH treatment. The bundle, called MOTIVE, consists of uterine Massage, Oxytocic drugs, Tranexamic acid, IntraVenous fluids and Examination and Escalation if required.

In-country adaptations
Oxytocin regimens varied considerably across countries and sites. The regimen selected for E-MOTIVE was commonly used in the participating countries and aligned with current recommendations. In Nigeria, Kenya, and Tanzania the volume of crystalloid for dilution of the initial oxytocin infusion (10 IU) was 500 mL, meanwhile in South Africa, it was (10IU) 100 mL or (10IU) 200 mL. Tranexamic acid (1g) was administered as a slow bolus injection over 10 minutes in Nigeria, Kenya, and Tanzania, whilst in South Africa it was diluted in 100 mL or 200 mL crystalloid and administered over 10 minutes, as a separate infusion to oxytocin infusion.

Key implementation points
Uterine massage
- **Massage of the uterus to stimulate contractions and expel any blood and blood clots**
  - Uterine massage for a maximum of 2 minutes initially.
  - Once contracted, checks undertaken every 15 minutes and massage repeated if the uterus became soft again.

Oxytocic drugs for the treatment of PPH
- **10 IU oxytocin intravenously** in a 200-500 mL bag of crystalloid fluid was administered at the fastest flow rate possible or approximately over 10 minutes. If an infusion was already in-situ it was replaced with a new bag of 10 IU of oxytocin in a 500 mL bag of crystalloid fluid. If there was a delay in starting the IV infusion, providers were asked to give 10 IU of oxytocin by IM injection or 800 micrograms of misoprostol sublingually or rectally until the oxytocin IV infusion could be started.
- **A maintenance infusion of 20 IU of oxytocin in 1000 mL of crystalloid fluid over 4 hours** was administered after the first oxytocin infusion finished.
- If local protocols used misoprostol and oxytocin simultaneously for the treatment of PPH, they could continue to follow this practice.

Tranexamic acid
- **1g Tranexamic acid intravenously (2 x ampoules 500mg/5mL)** as injection or diluted in 100 or 200 mL of crystalloid and administered over 10 minutes.
  - Tranexamic acid could be administered by midwives and nurses after completion of the training, without the need for a medical prescription.
  - A second dose of Tranexamic acid could be given if bleeding continued 30 minutes after the first dose.
  - Tranexamic acid could not be used in women with a clear contraindication to antifibrinolytic therapy. This would include a known thromboembolic event during any pregnancy, labour, or childbirth; severe renal failure; history of convulsions or hypersensitivity to tranexamic acid.
  - To avoid the unintentional medication error of intra-thecal injection of tranexamic acid, we instructed hospitals to avoid storing tranexamic acid near anaesthetic drugs.

IV fluids

- IV fluids were co-administered with the initial oxytocin infusion (500 mL) or as a standalone infusion (500 or 1000 mL); additional fluids could be given if clinically indicated for resuscitation.

<u>**Examination of the genital tract**</u>

- **Careful inspection of the perineum, vagina, and cervix to identify genital tract tears.**
  - If a tear was identified as the source of bleeding, it was repaired. If repair of the tear was beyond the provider's competency, they were asked to apply pressure with clean gauze and call for help.
- **Emptying or checking the bladder**
  - The bladder was emptied with a catheter if found to be distended, or if the woman had not emptied it in the preceding hour or if this was usual local practice.
- **Checking the placenta for completeness**
  - Providers were trained to identify the two sides of the placenta and the membranes and asked to look for large missing pieces.

<u>**Escalation, when required**</u>

- **Providers escalated without delay if any of the following occurred:**
  - There was brisk, uncontrollable bleeding
  - Blood loss ≥ 1000 mL in the calibrated drape
  - The patient had become hypotensive or collapsed
  - Extensive genital tract trauma, including third degree tear (suturing was performed by a skilled provider attending to the woman)
- If providers judged that the bleeding continued after the MOTIVE bundle was administered, they started **treatment for refractory PPH as follows:**
  - Called for additional help; secured a second IV access; obtained blood for full blood count, clotting and crossmatch; ordered blood products and infused further IV fluids as per local protocols.
  - If atony continued, further interventions were implemented as per local protocols. These included: additional or different uterotonics, bimanual uterine compression, non-pneumatic anti-shock garment (NASG), uterine balloon tamponade, and surgical management. A second dose of tranexamic acid was administered if more than 30 minutes had passed since the first dose and bleeding continued.
  - If there was continued bleeding despite initial assessment and management, providers checked for deep vaginal or cervical lacerations. If deep tears were found that first responders could not suture, a temporising vaginal pack was used until help arrived. While providers waited for additional help or transfer to higher level of care, they continued supportive care of the woman including monitoring vital signs, IV fluid resuscitation, and shock management as per local protocols.

Implementation Strategies

PPH Trolley and Carry Case

The purpose of the trolleys and carry cases was to help health workers treat PPH efficiently by having all the necessary medicines and equipment in one place. They also acted as a physical prompt for the timely and consistent use of recommended interventions for PPH treatment and ensure facility preparedness for treating PPH.

In-country adaptations

This strategy was adapted to local needs. Facilities with space constraints opted for carry cases with essential medicines and equipment for the first response PPH treatment for at least one woman. Facilities with no space constraints opted for trolleys equipped to treat up to three women. The trolleys were stocked with medicines and equipment needed for both first-response and refractory treatment for PPH. Stocking of trolleys or carry cases was adapted for each site to match shift patterns and local preferences.

Key implementation points

- The champions were responsible for the initial trolley (Figure S2) or carry case(Figure S3) set up at their facility.
- The trolleys or carry-cases were placed in easily accessible locations within the labour ward.
- Essential medicines that did not require a cold chain were stored in the trolley or carry case. Oxytocin, which requires a cold chain, was stored in a refrigerator on the labour ward.
- A trolley and carry case were set up for training demonstrations and healthcare workers had the opportunity to become familiar with them during practice sessions. Contents of the trolleys or carry cases were arranged and labelled as per local preferences.
- The E-MOTIVE trolley and carry case had an accompanying checklist (Figure S4) detailing all the medicines and equipment to be stocked.
- A designated member of the clinical team at each site was responsible for stocking the trolley or carry case. Each site decided on whether the restocking took place at every shift or every day.

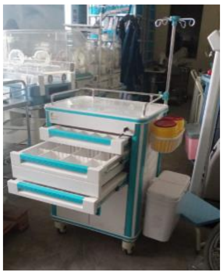

Figure S2: PPH Trolley

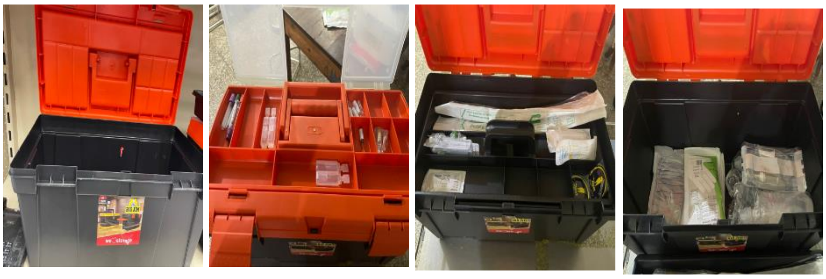

Figure S3: PPH Carry Case

[COUNTRY NAME] Name of Hospital: ____________________________

Week starting DD/MM/YYYY

**PPH Trolley** ITEM	Number of items	✓ Tick each item is in the trolley<br>**Check expiry date of medication**<br>Initial, write date/time at the bottom of the column once trolley is checked and fully stocked<br>**If items are missing, inform E-MOTIVE research staff, Champions and Hub team to ensure all items are available**						
		Please ✓ Monday	Please ✓ Tuesday	Please ✓ Wednesday	Please ✓ Thursday	Please ✓ Friday	Please ✓ Saturday	Please ✓ Sunday
Large Bore IV cannula	x 6							
Tourniquet	x 2							
Alcohol swabs	x 6							
10ml sterile water for injection	x 6							
Medical tape/plaster	x 2							
**TXA** 500mg/5mL vials<br>OR 1gm/10mL vials	x 12							
**Misoprostol** (200 mcg tablets)	x 2 full sheet							
Lignocaine 1% 10mL	x 3							
21G needles (GREEN)	x 12							
23G needles (BLUE)	x 3							
5mL syringes	x 3							
10mL syringes	x 15							
Additive stickers for IV fluids	x 3							
1000ml IV fluids (NaCl or Ringer's Lactate)	x 3							
500ml IV fluids (NaCl or Ringer's Lactate)	x 3							
Giving set	x 6							
Simms speculum	x 6							
Cusco bivalved speculum	x 3							
UBT (Foleys catheter & condom)	x 1							
Sponge-holding forceps	x 9							
SMALL packet sterile gauze	x 6							
MEDIUM packet sterile gauze	x 3							
Catheter bag	x 3							
Foley's urine catheter	x 3							
Size SMALL sterile gloves	x 3							
Size MEDIUM sterile gloves	x 3							
Size LARGE sterile gloves	x 3							
Box non-sterile gloves (MEDIUM)	x 1							
Kidney dish	x 3							
Small sharps container	x 1							
Plastic apron	x 9							
Cradle device [Fully charged]	x 1							
Confirm Action Plan is clean and attached to trolley	1x Action Plan							
Head torch [fully functional]	X 2							
**Date, time, initials**								

[COUNTRY NAME] Name of Hospital: ____________________________

Week starting DD/MM/YYYY

**PPH carry case** ITEM	Number of items	✓ Tick each item is in the trolley<br>**Check expiry date of medication**<br>Initial, write date/time at the bottom of the column once trolley is checked and fully stocked<br>**If items are missing, inform E-MOTIVE research staff, Champions and Hub team to ensure all items are available**						
		Please ✓ Monday	Please ✓ Tuesday	Please ✓ Wednesday	Please ✓ Thursday	Please ✓ Friday	Please ✓ Saturday	Please ✓ Sunday
Large Bore IV cannula	x 2							
Tourniquet	x 2							
Alcohol swabs	x 6							
10ml sterile water for injection	x 4							
Medical tape/plaster	x 2							
**TXA** 500mg/5mL vials<br>OR 1gm/10mL vials	x 10							
21G needles (GREEN)	x 6							
23G needles (BLUE)	x 6							
5mL syringes	x 6							
10mL syringes	x 8							
Additive stickers for IV fluids	x 3							
1000mL IV fluids (NaCl or Ringer's Lactate)	x 1							
500mL IV fluids (NaCl or Ringer's Lactate)	x 2							
Giving set	x 2							
SMALL packet sterile swabs	x 2							
MEDIUM packet sterile swabs	x 2							
Catheter bag	x 1							
Foley's catheter	x 1							
Size SMALL sterile gloves	x 2							
Size MEDIUM sterile gloves	x 2							
Size LARGE sterile gloves	x 2							
Lignocaine 1% 10mL	x 1							
Plastic apron	x 1							
Cradle device [Fully charged]	x 1							
**Date, time, initials**								

Figure S4. Trolley and carry case checklist

Training & Clinical protocols

The purpose of training and clinical protocols was to improve the knowledge and skills of healthcare workers on the early detection and bundled treatment of PPH. The guiding principles were "low-dose / high-frequency" simulation-based on-site multidisciplinary training. The training program was developed in partnership with Jhpiego (www.jhpiego.org).

In-country adaptations

The training included specific skills for midwives and nurses to administer all E-MOTIVE drugs, including tranexamic acid, safely when required without medical prescription. A clinical protocol was developed for each facility and approved by the hospital leadership for midwives and nurses to administer essential drugs.

Key implementation points

Subject areas covered

- Providing respectful care and communication to women and their families
- Communicating respectfully and effectively with other providers
- Preparing for birth / Prepare for PPH
    - Emergency trolley checklist
    - Recommended steps for maintaining oxytocin quality at the facility level
- Main causes of PPH
- Early detection of PPH
- Calling for help
- Triggers to begin MOTIVE
- MOTIVE bundle
    - How to massage the uterus
    - How to give oxytocic drugs
    - How to give tranexamic acid
    - How to start an IV infusion
    - How to examine and escalate
- Assessing clotting status
- Communicating during an emergency using SBAR: Situation-Background-Assessment-Recommendation
- Refractory bleeding: causes, assessment, management and escalation
- Low-dose high-frequency training

Organisation of E-MOTIVE training

- **Training of Trainers (ToT):** Core regular labour ward staff (3 to 4 per health facility) attended the ToT. Staff included the doctor champion, midwife champion, E-MOTIVE research midwife and other facility staff identified by the champions.
- **On-site training:** Members of staff who attended the ToT facilitated on-site training rollout at their health facilities. Training was held in multiple sessions to ensure all staff were trained, and were re-run as necessary to account for new staff.
- **Supervisory visits:** E-MOTIVE hospitals had supervisory visits by the trainers in months 1 and 3 of the 7 month intervention phase.
- Training materials for ToT and on-site training included:
    - Facilitator flipchart – a large-sized teaching aid used during the ToT and on-site training, containing the information from the provider's guide

- Provider's guide – contains the E-MOTIVE training information for both facilitators and providers; all attendees receive a handheld version for easy reference to the teaching
- Action plan – a graphic job aid to help providers identify and manage PPH
- MamaNatalie simulator – birthing simulator used for simulated practice sessions (Figure S5)

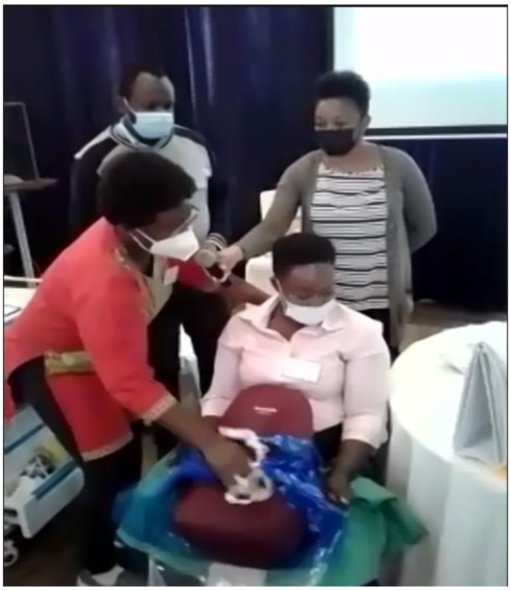

Figure S5. MamaNatalie simulator in use during an E-MOTIVE practice session

Champions

The purpose of the champions was to promote and sustain the implementation of E-MOTIVE. Champions were trained to train and support other healthcare workers to implement E-MOTIVE.

In-country adaptations

Stakeholders from each country opted for two champions (a doctor and a midwife) per facility. There was also a strong preference for champions to be selected based on capability, capacity, and commitment to carry out this role.

Key implementation points

Selection process: Two champions were selected by the health facility leadership, one doctor and one midwife with ample clinical experience and currently working in a clinical capacity in the facility's labour ward.

Role description:

- Be present in the labour ward across different shifts, including weekends.
- Be drivers of change in the facility to reduce the burden of PPH and improve outcomes.
- Become an E-MOTIVE trainer by attending the 'Training of Trainers' course.
- Support the use of the implementation strategies, use of the calibrated obstetric drape and clinical observations, E-MOTIVE training rollout, use of the PPH trolley or carry case and the Audit and Feedback process using the *E-Motive Monthly Audit Newsletter*
- Coordinate and run the on-site training and practice activities for all relevant labour ward staff in the facility, jointly with the other champion and trained clinical staff.

- Facilitate a positive learning environment, including supporting all staff to attend the on-site E-MOTIVE training and the ongoing practice activities.
- Offer to supervise and guide labour ward staff in implementing E-MOTIVE correctly.
- Lead by example in own clinical practice by following the PPH clinical protocol for early detection and treatment using the MOTIVE bundle.
- Work alongside labour ward staff, observing practices to provide positive feedback and specific feedback for targeted constructive improvements in implementing E-MOTIVE correctly.
- Review regular performance indicators and outcome data from their facility to assess if improvements were needed, using the *E-Motive Monthly Audit Newsletter.*
- Present the performance indicators and outcome data to staff using the *E-Motive Monthly Audit Newsletter* at regular monthly meetings. Provide specific feedback on where improvements were needed.
- Work together with the labour ward staff to understand the root causes of the local challenges and address any improvements required in implementing E-MOTIVE.
- If required, act on concerns and barriers to E-MOTIVE implementation and escalate promptly to hospital management to find solutions.
- Work with the champion community to identify ways to improve performance and address implementation challenges.
- Be resourceful and proactive on how to improve the hospital's performance based on what has worked in other hospitals in consultation with the champion community.

Support and resources provided

<u>Champion induction training</u>

Brief overview in the Training of the Trainers course, covering:

- Rationale for E-MOTIVE
- Why we need champions
- What makes a great champion
- What the champion will do

<u>Champions Leadership and Management Skills for Healthcare Professionals course</u>

1-day virtual course hosted by University of Birmingham. Training course included the following sessions:

- How to be an effective leader
- How to be an effective manager
- How to resolve conflict
- How to bring about change
- How to run an audit programme
- How to develop and run a project
- Effective networking
- Effective handling of meetings
- Tackling complex problems
- Effective communication and negotiation
- Risk management and root cause analysis
- How to run an effective education and training programme

<u>Champion community</u>

- Connected champions from facilities across other regions and counties to share experiences and troubleshoot problems.
- Had access to champion's network portal hosted by University of Birmingham (https://www.emotivepph.com/)
- App version available (android)

- Attended and contributed to monthly virtual or face-to-face champion meetings organised by country Hub teams.

Audit and Feedback

The purpose of the audit & feedback strategy was to provide monthly feedback on the key implementation and clinical outcomes, with the aim of catalysing positive action. Information was made available on PPH detection rates and the use of key components of the PPH treatment bundle, as well as clinical outcomes such as PPH, severe PPH, blood transfusion, laparotomy, and deaths.

In-country adaptations

The implementation and clinical outcomes, the format and frequency of audit were adapted according to health workers' preferences. In addition, champions wanted to benchmark their facility performance against national average rates for implementation and clinical outcomes.

Key implementation points

- The E-MOTIVE Monthly Audit Newsletter was generated automatically from the trial dataset monthly and shared with each facility.
- A sample of the E-MOTIVE Monthly Audit Newsletter is shown in Figure S6, along with the explanation of each item.
- The E-MOTIVE Monthly Audit Newsletter was printed and displayed in a prominent location on labour ward. Each month's audit newsletter was displayed side-by-side with preceding months' newsletters.
- The audit and feedback Newsletter was discussed in existing monthly departmental meetings. If monthly departmental meetings did not exist, they were initiated and chaired by the E-MOTIVE champions.
- During the meetings, the performance indicators and outcome data were discussed, and targets and strategies for improvement were agreed.
- The champions across all facilities in a country met virtually or in person every month or every two months to learn and share best practice.

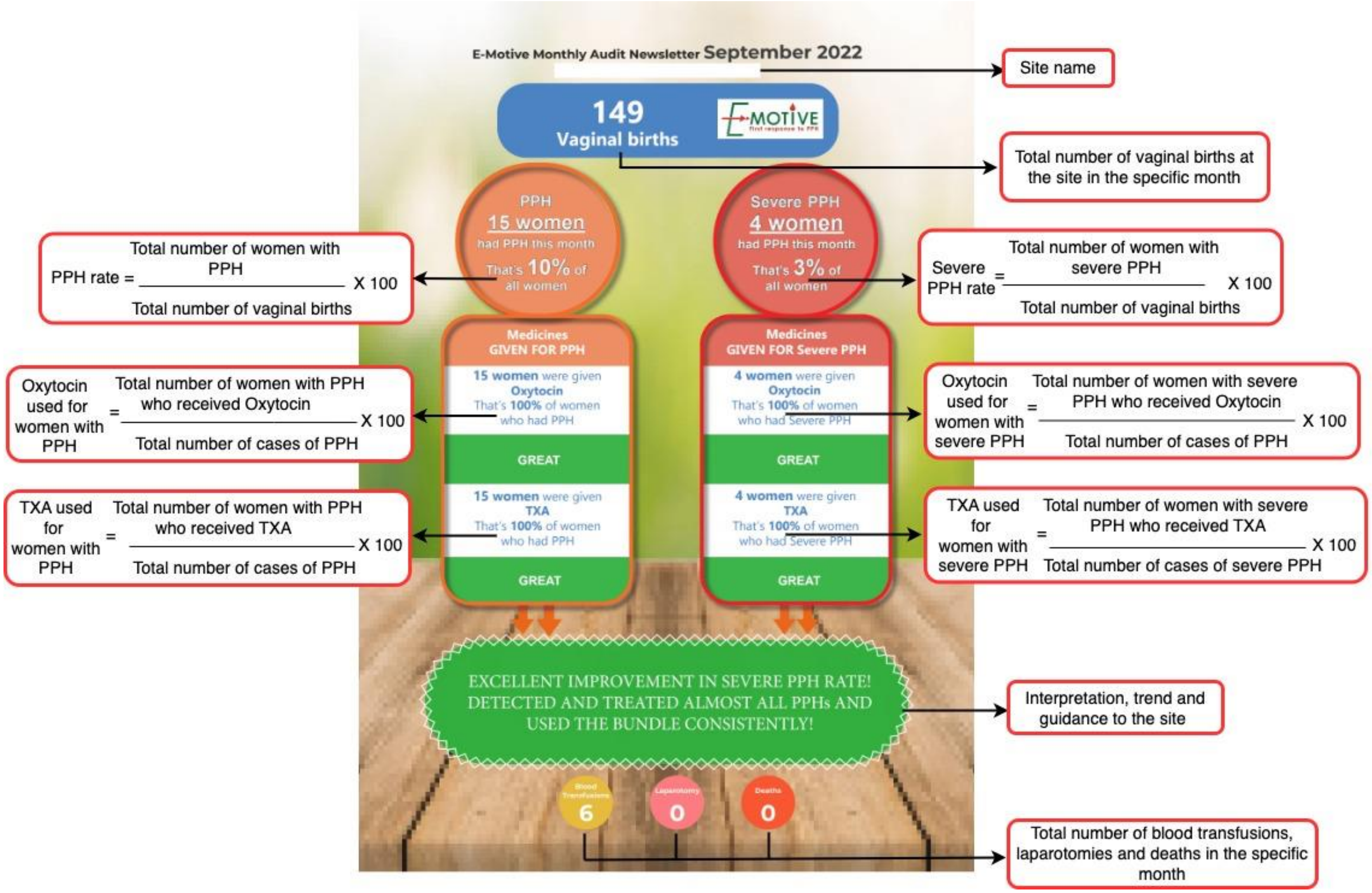

Figure S6. Sample of an E-MOTIVE monthly audit and feedback with the variable explained (red box); 90-100% for the key implementation outcomes was the target and shown with green colour and 'GREAT' as feedback. For below 90% it was shown as amber and 'NEEDS IMPROVEMENT' as feedback.

Appendix 3. Standards for Reporting Implementation Studies: the StaRI checklist for completion

The StaRI standard should be referenced as: Pinnock H, Barwick M, Carpenter C, Eldridge S, Grandes G, Griffiths CJ, Rycroft-Malone J, Meissner P, Murray E, Patel A, Sheikh A, Taylor SJC for the StaRI Group. Standards for Reporting Implementation Studies http://www.bmj.com/content/356/bmj.i6795.full. *BMJ* 2017;356:i6795

The detailed Explanation and Elaboration document, which provides the rationale and exemplar text for all these items is: Pinnock H, Barwick M, Carpenter C, Eldridge S, Grandes G, Griffiths C, Rycroft-Malone J, Meissner P, Murray E, Patel A, Sheikh A, Taylor S, for the StaRI group. Standards for Reporting Implementation Studies http://bmjopen.bmj.com/content/7/4/e013318.full?ijkey=vv4LKZxc25YcLJv&keytype=ref. *BMJ Open* 2017 2017;7:e013318

Notes: A key concept of the StaRI standards is the dual strands of describing, on the one hand, the implementation strategy and, on the other, the clinical, healthcare, or public health intervention that is being implemented. These strands are represented as two columns in the checklist.

The primary focus of implementation science is the implementation strategy (column 1) and the expectation is that this will always be completed.	The evidence about the impact of the intervention on the targeted population should always be considered (column 2) and either health outcomes reported or robust evidence cited to support a known beneficial effect of the intervention on the health of individuals or populations.

The StaRI standards refers to the broad range of study designs employed in implementation science. Authors should refer to other reporting standards for advice on reporting specific methodological features. Conversely, whilst all items are worthy of consideration, not all items will be applicable to, or feasible within every study.

Checklist item		Reported on page #	Implementation Strategy	Reported on page #	Intervention
			"Implementation strategy" refers to how the intervention was implemented		"Intervention" refers to the healthcare or public health intervention that is being implemented.
**Title and abstract**					
Title	1	We report as a mixed-methods process evaluation, which is more appropriate given the study design and methodology	Identification as an implementation study, and description of the methodology in the title and/or keywords		
Abstract	2	We report as a mixed-methods process evaluation, which is more appropriate given the study design and methodology. Description	Identification as an implementation study, including a description of the implementation strategy to be tested, the evidence-based intervention being implemented, and defining the key implementation and health outcomes.		

		of intervention and implementation strategy and outcomes included in abstract.			
Introduction					
Introduction	3	Page 6	Description of the problem, challenge or deficiency in healthcare or public health that the intervention being implemented aims to address.		
Rationale	4	Page 6	The scientific background and rationale for the implementation strategy (including any underpinning theory/framework/model, how it is expected to achieve its effects and any pilot work).	Page 6	The scientific background and rationale for the intervention being implemented (including evidence about its effectiveness and how it is expected to achieve its effects).
Aims and objectives	5	Page 7	The aims of the study, differentiating between implementation objectives and any intervention objectives.		
Methods: description					
Design	6	Page 7	The design and key features of the evaluation, (cross referencing to any appropriate methodology reporting standards) and any changes to study protocol, with reasons		
Context	7	Page 7, original trial publication available here: https://www.nejm.org/doi/full/10.1056/NEJMoa2303966#sec-2.	The context in which the intervention was implemented. (Consider social, economic, policy, healthcare, organisational barriers and facilitators that might influence implementation elsewhere).		
Targeted 'sites'	8	Page 6-7	The characteristics of the targeted 'site(s)' (e.g., locations/personnel/resources etc.) for implementation and any eligibility criteria.	Page 6-7	The population targeted by the intervention and any eligibility criteria.
Description	9	Briefly page 6, in detail in Appendix 2	A description of the implementation strategy	Briefly page 6, in detail in Appendix 2	A description of the intervention
Sub-groups	10	Not applicable	Any sub-groups recruited for additional research tasks, and/or nested studies are described		
Methods: evaluation					
Outcomes	11	Page 7-8, table 1	Defined pre-specified primary and other outcome(s) of the implementation strategy, and how they were assessed. Document any pre-determined targets	Page 7-8, table 1	Defined pre-specified primary and other outcome(s) of the intervention (if assessed), and how they were assessed. Document any pre-determined targets
Process evaluation	12	Page 6-7, with details reported in reference #18	Process evaluation objectives and outcomes related to the mechanism by which the strategy is expected to work		

		(Forbes 2024), an intervention development paper			
Economic evaluation	13	Economic evaluation published elsewhere: https://www.nature.com/articles/s41591-024-03069-5	Methods for resource use, costs, economic outcomes and analysis for the implementation strategy	Economic evaluation published elsewhere: https://www.nature.com/articles/s41591-024-03069-5	Methods for resource use, costs, economic outcomes and analysis for the intervention
Sample size	14	Page 7 (observation), page 8 (qualitative), page 9 (survey)	Rationale for sample sizes (including sample size calculations, budgetary constraints, practical considerations, data saturation, as appropriate)		
Analysis	15	Page 8 (observation and qualitative), page 9 (survey), page 9 (data integration across sources)	Methods of analysis (with reasons for that choice)		
Sub-group analyses	16	Not applicable	Any a priori sub-group analyses (e.g. between different sites in a multicentre study, different clinical or demographic populations), and sub-groups recruited to specific nested research tasks		
**Results**					
Characteristics	17	Table 2	Proportion recruited and characteristics of the recipient population for the implementation strategy	Table 2	Proportion recruited and characteristics (if appropriate) of the recipient population for the intervention
Outcomes	18	Primary outcomes of the trial are reported in the main trial report: https://www.nejm.org/doi/full/10.1056/NEJMoa2303966#sec-2	Primary and other outcome(s) of the implementation strategy	Primary outcomes of the trial are reported in the main trial report: https://www.nejm.org/doi/full/10.1056/NEJMoa2303966#sec-2	Primary and other outcome(s) of the Intervention (if assessed)
Process outcomes	19	Page 9-15, Tables 3-6, Figure 2, Appendices	Process data related to the implementation strategy mapped to the mechanism by which the strategy is expected to work		
Economic evaluation	20	Economic evaluation published elsewhere:	Resource use, costs, economic outcomes and analysis for the implementation strategy	Economic evaluation	Resource use, costs, economic outcomes and analysis for the intervention

		https://www.nature.com/articles/s41591-024-03069-5		published elsewhere: https://www.nature.com/articles/s41591-024-03069-5	
Sub-group analyses	21	Not applicable	Representativeness and outcomes of subgroups including those recruited to specific research tasks		
Fidelity/ adaptation	22	Page 9-14 (fidelity results reported across calibrated drape, MOTIVE treatment bundle, and 4 implementation strategies)	Fidelity to implementation strategy as planned and adaptation to suit context and preferences	Page 9-14 (fidelity results reported across calibrated drape, MOTIVE treatment bundle, and 4 implementation strategies)	Fidelity to delivering the core components of intervention (where measured)
Contextual changes	23	Page 14-15	Contextual changes (if any) which may have affected outcomes		
Harms	24	Not applicable	All important harms or unintended effects in each group		
Discussion					
Structured discussion	25	Page 15-16	Summary of findings, strengths and limitations, comparisons with other studies, conclusions and implications		
Implications	26	Page 15-16	Discussion of policy, practice and/or research implications of the implementation strategy (specifically including scalability)	Page 15-16	Discussion of policy, practice and/or research implications of the intervention (specifically including sustainability)
General					
Statements	27	Page 17, Appendix 1	Include statement(s) on regulatory approvals (including, as appropriate, ethical approval, confidential use of routine data, governance approval), trial/study registration (availability of protocol), funding and conflicts of interest		

Appendix Figure 1. The E-MOTIVE intervention

Reproduced with permission from: Gallos I, Devall A, Martin J, et al. Randomized Trial of Early Detection and Treatment of Postpartum Hemorrhage. New England Journal of Medicine 2023; 389(1): 11-21. https://www.nejm.org/doi/full/10.1056/NEJMoa2303966

E	M	O	T	IV	E
**Early Detection and Trigger Criteria**	**Massage of Uterus**	**Oxytocic Drugs**	**Tranexamic Acid**	**IV Fluids**	**Examination and Escalation**
Calibrated drape for the the collection of blood, with trigger lines at 300 ml and 500 ml for the first hr after birth<br>Observations (blood loss, blood flow, uterine tone) every 15 min documented on the blood-loss monitoring chart<br>Blood pressure and pulse monitored once in the first hr post partum and documented on the blood-loss monitoring chart<br>**Trigger Criteria**<br>Clinical judgment<br>Blood loss ≥500 ml<br>Blood loss ≥300 ml plus one abnormal observation	Massage until uterus has contracted or for 1 min	10 IU IV oxytocin injected or diluted in 200–500 ml crystalloid administered over 10-min period, plus a maintenance dose of 20 IU IV oxytocin diluted in 1000 ml saline administered over 4-hr period (with misoprostol 800 μg if used)	1 g IV tranexamic acid injected or diluted in 200 ml crystalloid administered over 10-min period	IV fluids in addition to the infusion should be given if clinically indicated for resuscitation and will require a second intravenous access	Ensure bladder is empty, evacuate clots, check for tears with an internal examination and placenta for completeness<br>Escalate if bleeding does not stop after first response or clinician is unable to identify or manage cause of bleeding

Implementation Strategies

Audit newsletters: Sharing with all staff monthly rates of detection and bundle use, along with rates of PPH, severe PPH, blood transfusion, laparotomy, and death from PPH and giving feedback at monthly departmental meetings

Champions: Midwife and doctor to oversee change, troubleshoot, give feedback on audit newsletters, connect with other champions by means of chats, meetings, and websites for sharing knowledge and lessons learned

Trolley or carry case: Restocking of all medicines and devices used for treatment of PPH after every use and completion of a stocking checklist at the start of every shift

Training: Onsite, simulation-based, and peer-assisted training, lasting from 90 min to an entire workday, facilitated by the use of provider guides, flipcharts, and job aids displayed in labor wards

Appendix Figure 2. E-MOTIVE Research Programme

This figure depicts the structure of the E-MOTIVE Research Programme, including the formative phase, intervention phase, and post-intervention phase. This paper presents the analysis of the trial process evaluation, and results from the other phases are presented elsewhere.

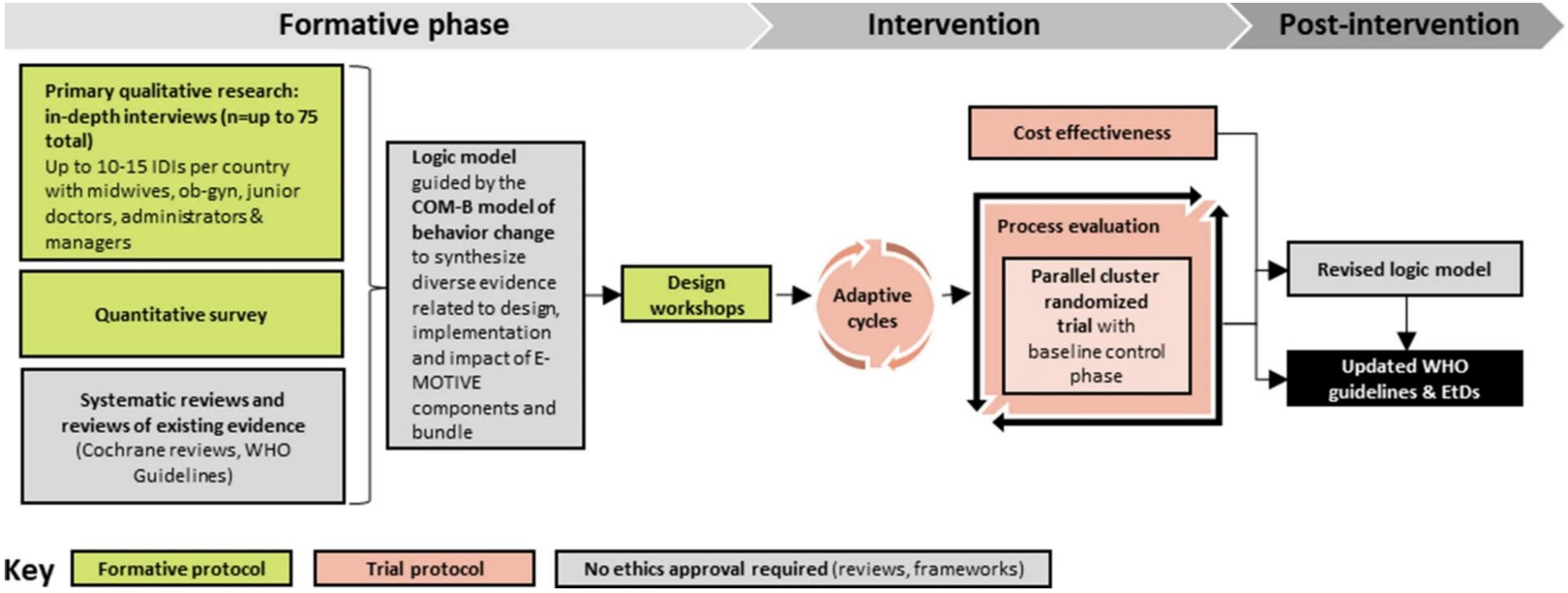

Appendix Figure 3: Intervention survey data: acceptability and feasibility of implementation strategies

This figure depicts frequency data from the cross-sectional survey conducted in intervention sites, about health workers' acceptability and feasibility of the E-MOTIVE implementation strategies: PPH trolley or carry case, audit and feedback, simulation-based, on-site training, and PPH champions.

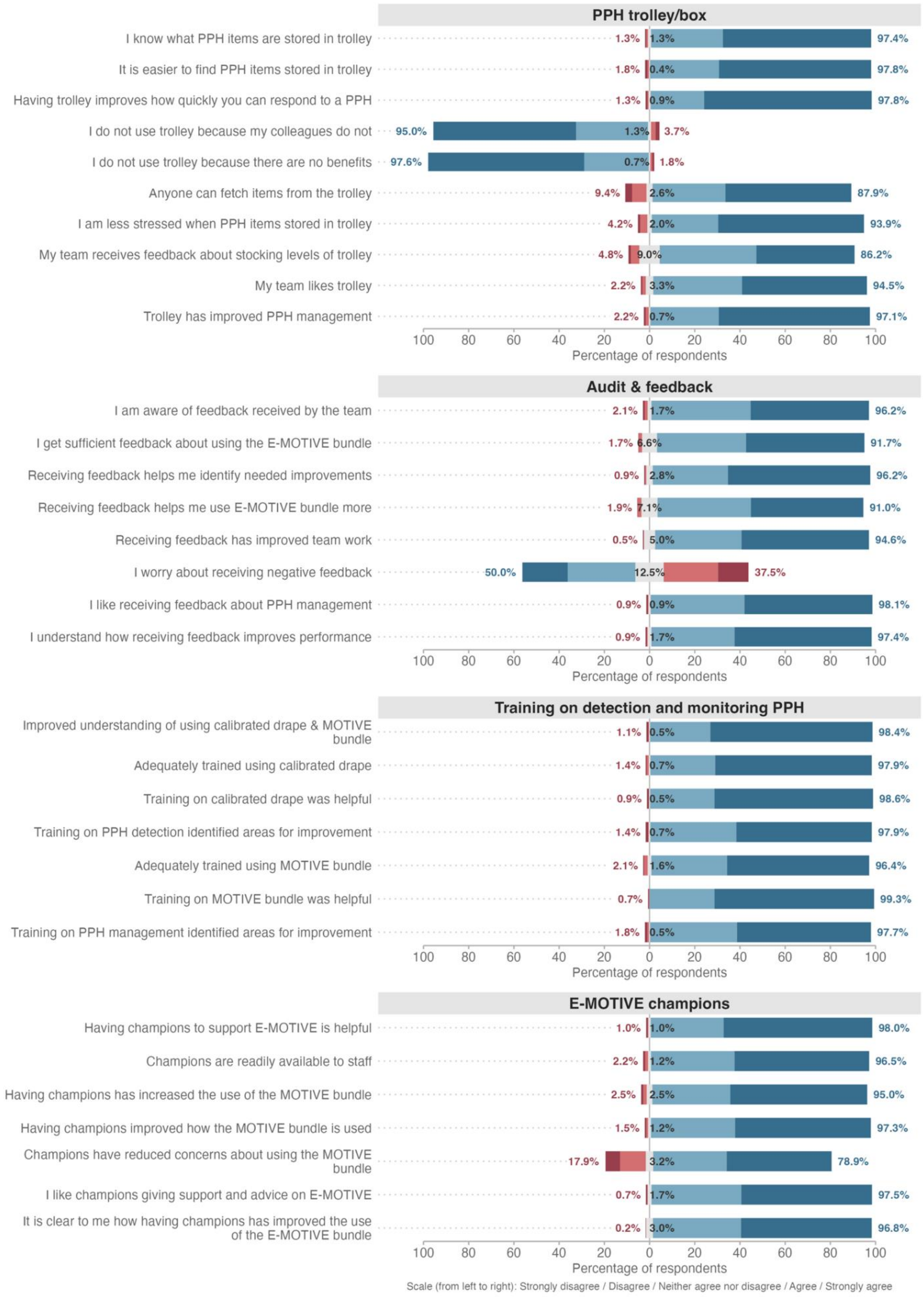

Appendix Figure 4: Control survey data: PPH detection and management

This figure depicts frequency data from the cross-sectional survey conducted in control sites, about use of the uncalibrated blood-collection drape (used for trial outcome measurement) and approaches to PPH management, to assess potential threats of contamination.

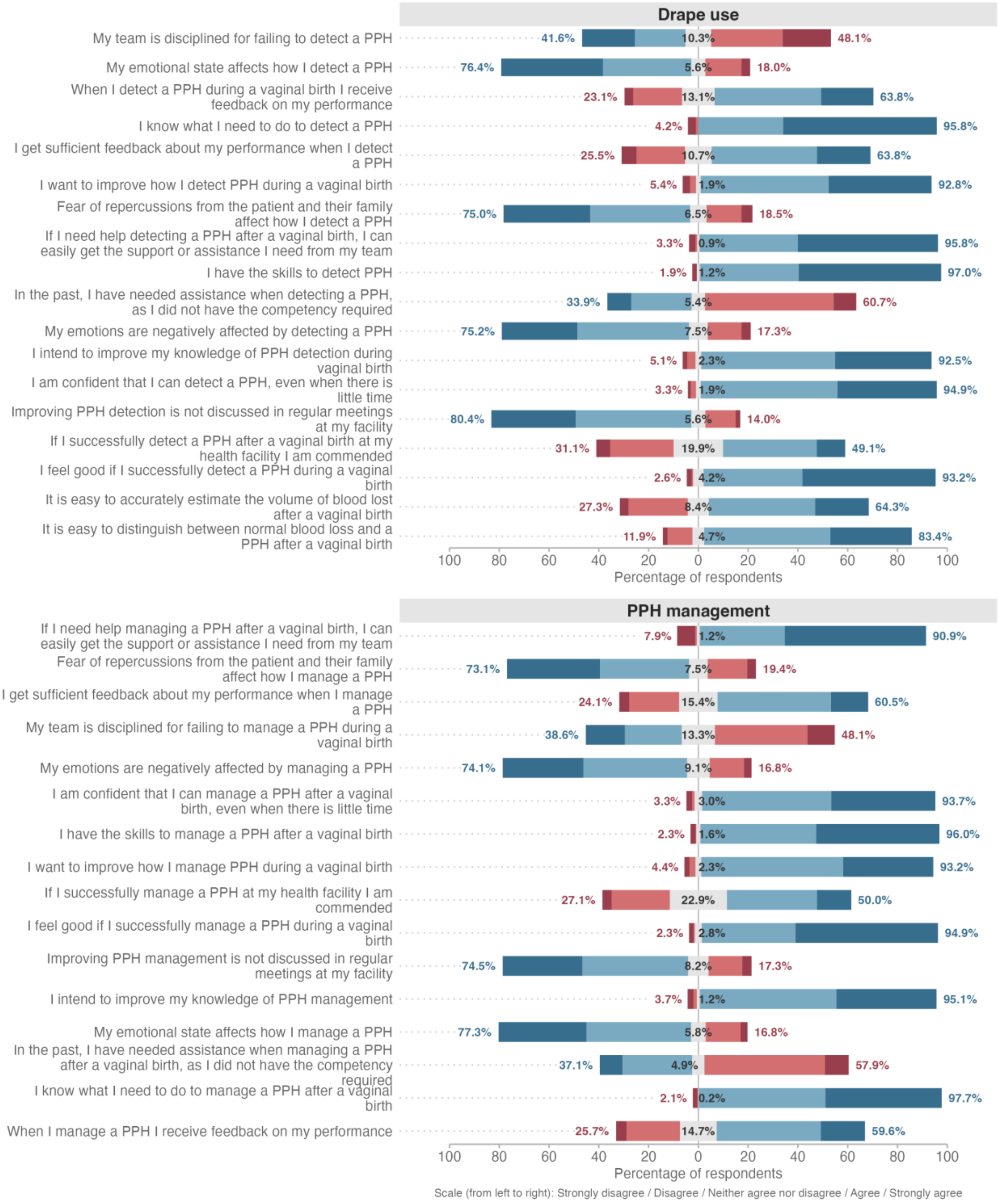

Appendix Table 1. Sociodemographic information.

This table presents sociodemographic information (frequencies and percentages) about participants across data sources and countries.

	Kenya	Nigeria	South Africa	Tanzania	Total
**Observations**					
**Women with vaginal birth**	734 (28·5%)	925 (35·9%)	268 (10·3%)	651 (25·3%)	2,578
**Women with PPH**	75 (25·4%)	141 (47·8%)	57 (19·4%)	22 (7·4%)	295
**Qualitative interviews: intervention sites**	8 (25·0%)	8 (25·0%)	8 (25·0%)	8 (25·0%)	2,578
**Doctor**	3 (33·4%)	2 (22·2%)	2 (22·2%)	2 (22·2%)	295
**Midwife/nurse-midwife**	2 (13·4%)	5 (33·3%)	3 (20·0%)	5 (33·3%)	32 (100·0%)
**Nurse**	2 (25·0%)	3 (37·5%)	2 (25·0%)	1 (12·5)%	9 (28·1%)
**Qualitative interviews: control sites**	4 (26·7%)	4 (26·7%)	3 20%)	4 (26·7%)	15 (46·9%)
**Doctor**	2 (40·0%)	1 (20·0%)	1 (20·0%)	1 (20·0%)	8 (25·0%)
**Midwife/nurse-midwife**	1 (14·3%)	1 (14·3%)	2 (28·6%)	3 (42·8%)	15 (100·0%)
**Nurse**	1 (33·3%)	2 (66·7%)	0 (0%)	0 (0%)	5 (33·3%)
**Surveys: Intervention sites**	128 (27·8%)	181 (39·3%)	62 (13·4%)	90 (19·5%)	461 (100·0%)
**Doctor**	33 (25·8%)	78 (43·1%)	27 (43·5%)	13 (14·4%)	151 (32·8%)
**Midwife/nurse-midwife**	65 (50·8%)	96 (53·0%)	35 (56·5%)	69 (76·7%)	265 (57·5%)
**Nurse**	30 (23·4%)	7 (3·9%)	0 (0·0%)	8 (8·9%)	45 (9·8%)
**Surveys: Control sites**	130 (30·4%)	158 (36·9%)	53 (12·4%)	87 (20·3%)	428 (100·0%)
**Doctor**	37 (28·5%)	75 (47·5%)	15 (28·3%)	24 (27·6%)	151 (35·3%)
**Midwife/nurse-midwife**	61 (46·9%)	81 (51·3%)	38 (71·7%)	62 (71·3%)	242 (56·5%)
**Nurse**	32 (24·6%)	2 (1·3%)	0 (0·0%)	1 (1·1%)	35 (8·2%)

Appendix Table 2: Intervention survey data: sociodemographic characteristics

This table presents detailed sociodemographic information (frequencies and percentages) about participants in the cross-sectional survey in the intervention sites.

	Kenya n=128	Nigeria n=181	South Africa n=62	Tanzania n=90	Total n=461	p value†
**Current position**						
Nurse-midwife	65 (50·8%)	96 (53·0%)	35 (56·5%)	69 (76·7%)	265 (57·5%)	<0.0001
Doctor	33 (25·8%)	78 (43·1%)	27 (43·5%)	13 (14·4%)	151 (32·8%)	
Nurse	30 (23·4%)	7 (3·9%)	0 (0·0%)	8 (8·9%)	45 (9·8%)	
**Total years of experience in this position**						
0–5 years	70 (54·7%)	96 (53·0%)	33 (53·2%)	45 (50·0%)	244 (52·9%)	0.93
≥6 years	58 (45·3%)	85 (47·0%)	29 (46·8%)	45 (50·0%)	217 (47·1%)	
**Total years working in this position at this facility**						
0–5 years	95 (74·2%)	108 (59·7%)	44 (71·0%)	55 (61·1%)	302 (65·5%)	0.035
≥6 years	33 (25·8%)	73 (40·3%)	18 (29·0%)	35 (38·9%)	159 (34·5%)	
**Last time detected a PPH or assisted in PPH detection**						
≤7 days	105 (82·0%)	133 (73·5%)	43 (69·4%)	50 (55·6%)	331 (71·8%)	0.0003
>7 days to ≤6 months	23 (18·0%)	48 (26·5%)	19 (30·6%)	40 (44·4%)	130 (28·2%)	
**Last time managed or assisted in PPH management**						
≤7 days	102 (79·7%)	131 (72·4%)	43 (69·4%)	49 (54·4%)	325 (70·5%)	0.0008
>7 days to ≤6 months	26 (20·3%)	50 (27·6%)	19 (30·6%)	41 (45·6%)	136 (29·5%)	
**Managed PPH as a team**						
Yes – always	100 (78·1%)	143 (79·0%)	50 (80·6%)	71 (78·9%)	364 (79·0%)	1.0
Yes – most of the time	27 (21·1%)	37 (20·4%)	12 (19·4%)	19 (21·1%)	95 (20·6%)	
No – worked alone	1 (0·8%)	1 (0·6%)	0 (0·0%)	0 (0·0%)	2 (0·4%)	

†Fisher's exact and chi-squared tests were used.

Appendix Table 3: Control survey data: sociodemographic characteristics

This table presents detailed sociodemographic information (frequencies and percentages) about participants in the cross-sectional survey in the control sites.

	Kenya n=130	Nigeria n=158	South Africa n=53	Tanzania n=87	Total n=428	p value†
**Current position**						
Nurse-midwife	61 (46·9%)	81 (51·3%)	38 (71·7%)	62 (71·3%)	242 (56·5%)	<0·0001
Doctor	37 (28·5%)	75 (47·5%)	15 (28·3%)	24 (27·6%)	151 (35·3%)	
Nurse	32 (24·6%)	2 (1·3%)	0 (0·0%)	1 (1·1%)	35 (8·2%)	
**Total years of experience in this position**						
0–5 years	78 (60·0%)	67 (42·4%)	30 (56·6%)	50 (57·5%)	225 (52·6%)	0·014
≥6 years	52 (40·0%)	91 (57·6%)	23 (43·4%)	37 (42·5%)	203 (47·4%)	
**Total years working in this position at this facility**						
0–5 years	107 (82·3%)	89 (56·3%)	33 (62·3%)	54 (62·1%)	283 (66·1%)	<0·0001
≥6 years	23 (17·7%)	69 (43·7%)	20 (37·7%)	33 (37·9%)	145 (33·9%)	
**Last time detected a PPH or assisted in PPH detection**						
≤7 days	105 (80·8%)	122 (77·2%)	36 (67·9%)	77 (88·5%)	340 (79·4%)	0·026
>7 days to ≤6 months	25 (19·2%)	36 (22·8%)	17 (32·1%)	10 (11·5%)	88 (20·6%)	
**Last time managed or assisted in PPH management**						
≤7 days	105 (80·8%)	117 (74·1%)	36 (67·9%)	79 (90·8%)	337 (78·7%)	0·0034
>7 days to ≤6 months	25 (19·2%)	41 (25·9%)	17 (32·1%)	8 (9·2%)	91 (21·3%)	
**Managed PPH as a team**						
Yes – always	101 (77·7%)	115 (72·8%)	41 (77·4%)	76 (87·4%)	333 (77·8%)	0·18
Yes – most of the time	28 (21·5%)	41 (25·9%)	12 (22·6%)	11 (12·6%)	92 (21·5%)	
No – worked alone	1 (0·8%)	2 (1·3%)	0 (0·0%)	0 (0·0%)	3 (0·7%)	
**Training received in the last 2 years**	104 (80·0%)	92 (58·2%)	37 (69·8%)	40 (46·0%)	273 (63·8%)	<0·0001
**When the last training was received**						
≤3 months	50 (38·5%)	20 (12·7%)	13 (24·5%)	17 (19·5%)	100 (23·4%)	<0·0001
>3 to 6 months	20 (15·4%)	18 (11·4%)	3 (5·7%)	3 (3·4%)	44 (10·3%)	
>6 to <12 months	17 (13·1%)	21 (13·3%)	6 (11·3%)	5 (5·7%)	49 (11·4%)	
>12 months	17 (13·1%)	33 (20·9%)	15 (28·3%)	15 (17·2%)	80 (18·7%)	
**Did not received training**	26 (20·0%)	66 (41·8%)	16 (30·2%)	47 (54·0%)	155 (36·2%)	
**Any quality improvement initiatives in the last 6 months**						
Yes	68 (52·3%)	46 (29·1%)	21 (39·6%)	44 (50·6%)	179 (41·8%)	<0·0001
No	38 (29·2%)	56 (35·4%)	20 (37·7%)	34 (39·1%)	148 (34·6%)	
Don't know	24 (18·5%)	56 (35·4%)	12 (22·6%)	9 (10·3%)	101 (23·6%)	

†Fisher’s exact and chi-squared tests were used.

Appendix Table 4: Intervention survey data: calibrated drape and bundle use

This table presents data (frequency and percentages) from the cross-sectional survey conducted in intervention sites, about health workers' acceptability and feasibility of the calibrated blood-collection drape and MOTIVE bundle. This data is visually depicted in Figure 2.

	Kenya n=128	Nigeria n=181	South Africa n=62	Tanzania n=90	Total n=461	p-value†	Implementation outcome
**Other methods of PPH detection used**							
Visual estimation	40 (31·2%)	100 (55·2%)	40 (64·5%)	45 (50·0%)	225 (48·8%)	<0·0001	High fidelity
Vital signs	120 (93·8%)	163 (90·1%)	56 (90·3%)	80 (88·9%)	419 (90·9%)	0·60	
Uterine tone and size	110 (85·9%)	137 (75·7%)	44 (71·0%)	72 (80·0%)	363 (78·7%)	0·063	
Counting or weighing blood-soaked swabs	69 (53·9%)	93 (51·4%)	31 (50·0%)	60 (66·7%)	253 (54·9%)	0·086	
Blood loss monitoring chart	102 (79·7%)	117 (64·6%)	37 (59·7%)	79 (87·8%)	335 (72·7%)	<0·0001	
**Belief that visual estimation of blood loss is…**							
Very effective	10 (7·8%)	28 (15·5%)	16 (25·8%)	19 (21·1%)	73 (15·8%)	<0·0001	Acceptability
Somewhat effective	33 (25·8%)	74 (40·9%)	28 (45·2%)	23 (25·6%)	158 (34·3%)		
Not effective	85 (66·4%)	79 (43·6%)	18 (29·0%)	48 (53·3%)	230 (49·9%)		
**Belief that using vital signs is…**							
Very effective	94 (73·4%)	109 (60·2%)	35 (56·5%)	68 (75·6%)	306 (66·4%)	0·0078	Acceptability
Somewhat effective	32 (25·0%)	65 (35·9%)	21 (33·9%)	21 (23·3%)	139 (30·2%)		
Not effective	2 (1·6%)	7 (3·9%)	6 (9·7%)	1 (1·1%)	16 (3·5%)		
**Belief that using uterine tone and size is…**							
Very effective	94 (73·4%)	102 (56·4%)	30 (48·4%)	65 (72·2%)	291 (63·1%)	0·0004	Acceptability
Somewhat effective	30 (23·4%)	64 (35·4%)	21 (33·9%)	20 (22·2%)	135 (29·3%)		
Not effective	4 (3·1%)	15 (8·3%)	11 (17·7%)	5 (5·6%)	35 (7·6%)		
**Belief that counting/weighing blood-soaked swabs is…**							
Very effective	43 (33·6%)	54 (29·8%)	17 (27·4%)	38 (42·2%)	152 (33·0%)	0·010	Acceptability
Somewhat effective	58 (45·3%)	97 (53·6%)	37 (59·7%)	28 (31·1%)	220 (47·7%)		
Not effective	27 (21·1%)	30 (16·6%)	8 (12·9%)	24 (26·7%)	89 (19·3%)		
**Belief that blood loss monitoring chart is…**							
Very effective	101 (78·9%)	116 (64·1%)	34 (54·8%)	79 (87·8%)	330 (71·6%)	<0·0001	Acceptability
Somewhat effective	21 (16·4%)	59 (32·6%)	22 (35·5%)	8 (8·9%)	110 (23·9%)		
Not effective	6 (4·7%)	6 (3·3%)	6 (9·7%)	3 (3·3%)	21 (4·6%)		
**Reasons for not using PPH trolley, carry-case or box**							Mixed feasibility
Not adequately stocked	9 (7·0%)	26 (14·4%)	9 (14·5%)	2 (2·2%)	46 (10·0%)	0·0057	
Inconsistent supply	13 (10·2%)	26 (14·4%)	6 (9·7%)	2 (2·2%)	47 (10·2%)	0·021	
Incorrect supply	3 (2·3%)	5 (2·8%)	4 (6·5%)	1 (1·1%)	13 (2·8%)	0·31	
Not easily located	3 (2·3%)	8 (4·4%)	10 (16·1%)	1 (1·1%)	22 (4·8%)	0·0005	
Lack of space	5 (3·9%)	8 (4·4%)	1 (1·6%)	0 (0·0%)	14 (3·0%)	0·16	
PPH items were kept in another place	11 (8·6%)	9 (5·0%)	11 (17·7%)	1 (1·1%)	32 (6·9%)	0·0005	
Drugs kept in the fridge	8 (6·2%)	17 (9·4%)	14 (22·6%)	2 (2·2%)	41 (8·9%)	0·0001	

	Kenya n=128	Nigeria n=181	South Africa n=62	Tanzania n=90	Total n=461	p-value†	Implementation outcome
Forgot to use	0 (0·0%)	5 (2·8%)	9 (14·5%)	0 (0·0%)	14 (3·0%)	<0·0001	
Was not allowed to use because of role	1 (0·8%)	3 (1·7%)	0 (0·0%)	0 (0·0%)	4 (0·9%)	0·62	
Damaged	0 (0·0%)	4 (2·2%)	0 (0·0%)	1 (1·1%)	5 (1·1%)	0·29	
**Reasons for not attending practice drill sessions (n=64)**							
I was absent	5 (41·7%)	10 (28·6%)	1 (14·3%)	6 (60·0%)	22 (34·4%)	0·29	Mixed feasibility
No practice drill sessions held	3 (25·0%)	8 (22·9%)	4 (57·1%)	1 (10·0%)	16 (25·0%)		
No practice drill sessions held for my shift	4 (33·3%)	9 (25·7%)	2 (28·6%)	2 (20·0%)	17 (26·6%)		
Other	0 (0·0%)	8 (22·9%)	0 (0·0%)	1 (10·0%)	9 (14·1%)		

†Fisher's exact and chi-squared tests were used.

Appendix Table 5: Intervention observation data: clinical assessments in the first hour after birth among women with vaginal birth

This table presents data (frequency and percentages) from the observations of 2,578 women with vaginal birth conducted in intervention sites, about clinical assessments conducted at 30 minutes (second assessment), 45 minutes (third assessment, and 60 minutes (fourth assessment) after birth, and provides information about the implementation outcomes of fidelity and adoption. Clinical assessments at 15 minutes after birth are presented in Table 4 of the main paper.

	**Kenya n=734**	**Nigeria n=925**	**South Africa n=268**	**Tanzania n=651**	**Total** n=2578	**p-value†**	**Implementation outcome**
**2nd clinical assessment: completed by** (**n=1795**)							Mixed fidelity/adoption
Midwife (including student midwife or nurse)	545 (81.5%)	27 (4.7%)	143 (60.1%)	199 (64.2%)	914 (50.9%)	<0.0001	
Research midwife	121 (18.1%)	551 (95.3%)	0 (0.0%)	110 (35.5%)	782 (43.6%)		
Doctor (including medical student and intern)	3 (0.4%)	0 (0.0%)	21 (8.8%)	1 (0.3%)	25 (1.4%)		
Enrolled nurse assistant	0 (0.0%)	0 (0.0%)	74 (31.1%)	0 (0.0%)	74 (4.1%)		
**2nd clinical assessment: calibrated drape measurement lines checked** (**n=1795**)	663 (99.1%)	575 (99.5%)	235 (98.7%)	286 (92.3%)	1759 (98.0%)	<0.0001	High fidelity
**2nd clinical assessment: where was drape funnel lying when reading calibration lines** (**n=1759**)							Mixed fidelity
Hanging over edge of bed	373 (56.3%)	438 (76.2%)	227 (96.6%)	280 (97.9%)	1318 (74.9%)	<0.0001	
Flat on bed	290 (43.7%)	137 (23.8%)	8 (3.4%)	6 (2.1%)	441 (25.1%)		
**2nd clinical assessment: if flat on bed, how were calibrated drape lines read** (**n=441**)							Mixed fidelity
Moved to edge of bed	275 (94.8%)	18 (13.1%)	2 (25.0%)	6 (100.0%)	301 (68.3%)	<0.0001	
Visualised flat on bed	14 (4.8%)	22 (16.1%)	6 (75.0%)	0 (0.0%)	42 (9.5%)		
Lifted to eye level	1 (0.3%)	97 (70.8%)	0 (0.0%)	0 (0.0%)	98 (22.2%)		
**2nd clinical assessment: calibrated drape measurement lines documented** (**n=1795**)	665 (99.4%)	563 (97.4%)	235 (98.7%)	281 (90.6%)	1744 (97.2%)	<0.0001	High fidelity
**3rd clinical assessment: completed by** (**n=1403**)							Mixed fidelity/adoption
Midwife (including student midwife or nurse)	473 (82.8%)	23 (4.8%)	130 (60.5%)	86 (62.8%)	712 (50.7%)	<0.0001	
Research midwife	95 (16.6%)	457 (95.2%)	0 (0.0%)	51 (37.2%)	603 (43.0%)		
Doctor (including medical student and intern)	3 (0.5%)	0 (0.0%)	14 (6.5%)	0 (0.0%)	17 (1.2%)		
Enrolled nurse assistant	0 (0.0%)	0 (0.0%)	71 (33.0%)	0 (0.0%)	71 (5.1%)		
**3rd clinical assessment: calibrated drape measurement lines checked** (**n=1403**)	560 (98.1%)	480 (100.0%)	210 (97.7%)	121 (88.3%)	1371 (97.7%)	<0.0001	High fidelity
**3rd clinical assessment: where was drape funnel lying when reading calibration lines** (**n=1371**)							Mixed fidelity
Hanging over edge of bed	330 (58.9%)	366 (76.2%)	205 (97.6%)	118 (97.5%)	1019 (74.3%)	<0.0001	
Flat on bed	230 (41.1%)	114 (23.8%)	5 (2.4%)	3 (2.5%)	352 (25.7%)		
**3rd clinical assessment: if flat on bed, how were calibrated drape lines read** (**n=352**)							

	Kenya n=734	Nigeria n=925	South Africa n=268	Tanzania n=651	Total n=2578	p-value†	Implementation outcome
Moved to edge of bed	220 (95.7%)	18 (15.8%)	1 (20.0%)	3 (100.0%)	242 (68.8%)	<0.0001	Mixed fidelity
Visualised flat on bed	10 (4.3%)	21 (18.4%)	4 (80.0%)	0 (0.0%)	35 (9.9%)		
Lifted to eye level	0 (0.0%)	75 (65.8%)	0 (0.0%)	0 (0.0%)	75 (21.3%)		
**3rd clinical assessment: calibrated drape measurement lines documented (n=1403)**	566 (99.1%)	474 (98.8%)	210 (97.7%)	122 (89.1%)	1372 (97.8%)	<0.0001	
**4th clinical assessment: completed by** (**n=887**)							Mixed fidelity/adoption
Midwife (including student midwife or nurse)	233 (78.5%)	10 (2.8%)	87 (60.0%)	51 (59.3%)	381 (43.0%)	<0.0001	
Research midwife	61 (20.5%)	349 (97.2%)	0 (0.0%)	35 (40.7%)	445 (50.2%)		
Doctor (including medical student and intern)	3 (1.0%)	0 (0.0%)	8 (5.5%)	0 (0.0%)	11 (1.2%)		
Enrolled nurse assistant	0 (0.0%)	0 (0.0%)	50 (34.5%)	0 (0.0%)	50 (5.6%)		
**4th clinical assessment: calibrated drape measurement lines checked (n=887)**	291 (98.0%)	359 (100.0%)	144 (99.3%)	80 (93.0%)	874 (98.5%)	<0.0001	High fidelity
**4th clinical assessment: where was drape funnel lying when reading calibration lines** (**n=874**)							Mixed fidelity
Hanging over edge of bed	192 (66.0%)	279 (77.7%)	140 (97.2%)	79 (98.8%)	690 (78.9%)	<0.0001	
Flat on bed	99 (34.0%)	80 (22.3%)	4 (2.8%)	1 (1.2%)	184 (21.1%)		
**4th clinical assessment: if flat on bed, how were calibrated drape lines read** (**n=183**)							Mixed fidelity
Moved to edge of bed	99 (100.0%)	13 (16.5%)	1 (25.0%)	1 (100.0%)	114 (62.3%)	<0.0001	
Visualised flat on bed	0 (0.0%)	17 (21.5%)	3 (75.0%)	0 (0.0%)	20 (10.9%)		
Lifted to eye level	0 (0.0%)	49 (62.0%)	0 (0.0%)	0 (0.0%)	49 (26.8%)		
**4th clinical assessment: calibrated drape measurement lines documented** (**n=887**)	294 (99.0%)	355 (98.9%)	143 (98.6%)	80 (93.0%)	872 (98.3%)	0.0086	High fidelity

†Fisher's exact (with and without Monte Carlo simulation with 1 million replicates) and chi-squared tests were used.

Appendix Table 6: Qualitative themes related to each implementation outcomes for early detection of PPH

This table presents data (themes and exemplar quotations) from the qualitative interviews with health workers conducted in intervention sites, about early detection of PPH. The table is organised under each implementation outcome of interest: fidelity, adoption, adaptation, acceptability, and feasibility. A summary version of this table is presented in Table 5 of the main manuscript.

Themes	Kenya	Nigeria	South Africa	Tanzania
**Fidelity, adoption, adaptation**				
Consistent adoption of calibrated drape for early detection of PPH	'Currently, we are using the E-MOTIVE drape. Previously we used to estimate' (Midwife, #11)	'We used the drape, the E-MOTIVE drape' (Midwife, #05)	'We always use a drape. with each and every woman' (Nurse, #26)	'We use them on every mother delivering normally' (Doctor, #17)
Calibrated drape correctly used as intended	'We usually put the drape when the mother approaches the second stage of labour… then we put it immediately after they delivered the baby and delivered the placenta' (Midwife, #10)	'The drape is usually applied immediately after the mum delivered before the delivery of the placenta…It is applied beneath the patients, and it's tied on' (Midwife, #05)	we check your blood loss most every time and that we do most every 15 minutes for the first hour. Okay. And then after the first hour, if the bleeding is still continuing, then we do it for another hour (Midwife, #28)	'We use a drape to every woman with normal delivery, soon after delivery we put a drape before delivering the placenta and we manage for 1 hour to record drape weight at every 15minutes we read. But after one hour if the mother has no other problems and there is no progress of bleeding, we remove it but in case of PPH we might continue for the 2 hours and above until when the mother is stable' (Midwife, #23)
Vital signs consistently taken	'Yes, we do. We take blood pressure.... plus, pulse rate, respirations.'(Midwife, #9)	'We always take vital signs before the woman's delivered and after she deliver' (Midwife, #12)	.. specifically in visible signs of the patient --does she look pale?...Ask is she is thirsty you know, things like that. Are you feeling alright? And then the vital signs that includes are the blood pressure is your pulse, right?' (Midwife, #28)	'vital signs, we measure blood pressure and pulse rate' (Midwife, #23)
**Acceptability**				
Earlier and more accurate detection of PPH using the calibrated drape<br><br>*(COM-B: Reflective motivation + Psychological capability)*	'Now because we were using the calibrated drapes, it's more accurate. And you're ...we're more likely to detect PPH earlier than when you were just doing visual visual...visual inspection used to probably underestimate the loss of blood' (Doctor, #13)	'Because we are able to detect it early. That's immediately it reaches with the help of drapes, of course, the E-MOTIVE drapes, immediately we detect PPH' (Midwife, #02)	'I like it. It's a very good tool to use…That's quite a good thing [calibrated drape] I must say, for us to be able to diagnose a patient early enough to act early enough (Midwife, #28)	'The main problem was on detection, you detect when a patient is in late stages and has started to develop complications and others were ending up in organ failure and we were referring but now we don't have referrals due to PPH' (Doctor, #21)
Ease of use and cleanliness<br><br>*(COM-B: Reflective motivation + Psychological capability)*	'The drapes are easier for detection. Prior we just used to estimate using our eyes, sometimes we just use the green towels…. we've been cleaning the mother…. at such moments, sometimes blood flows on the floor' (Doctor, #14)	'Because it's less messy actually unlike before, taking deliveries on the clean bed and everything. It tends to get messy. But with drape it collects everything inside and …. it makes it neat actually' (Midwife, #02)	'it's easier for them to monitor and you know, to alert, otherwise everybody is normally assuming that there might be its individual dependent, depending on how they feel the blood loss is unless it's obviously a lot' (Doctor, #32)	'First it helps us on cleanliness because the drape is sterilized and once opened its used on a single patient after that you will measure the amount of blood, it will not be used to another patient, the other patient has her own new one. So it is good, it

				doesn't need to be sterilized, it's sterilized and single use'(Midwife, #17
Vital signs as valued prompts to action<br><br>*((COM-B: Psychological capability)*	'Taking action means for example, blood loss is 300 mls and the blood pressure is above 110 or 140, the systolic is abnormal that is now you have to intervene' (Midwife, #11)	'Vital signs are cardinal indicators. So, if any change happens…maybe you know the patient has PPH... maybe the pulse rate maybe to go low, and then blood pressure maybe high' (Midwife, #03)	'…why would my patient's blood pressure suddenly drop? Why would the pulse be high? So, you need to go and investigate physically and see…are they responding to the treatment that we're giving them' (Midwife, 28)	'If there are changes you…if HB was low or there is over bleeding than normal. You need to be close to the patient so that you understand what the problem is' (Midwife, #24)
Acceptability of the drape by women giving birth<br><br>*(COM-B: Social opportunity)*	Putting them in the drape is quite a challenge. Sometimes they refuse so you have to coax them or force them (Doctor, #14)	We tell them the purpose of it. It's because we want to measure your blood loss…. sometimes for some it's quite uncomfortable, but….I've never witnessed a patient that actually refused it. Because we properly explained to them before applying it (Midwife, #02)	You need to explain to your patients because they will feel uncomfortable if you don't explain why you have to put a drape and what is the reason for it, but so far, we didn't have any problems without patients (Midwife, #27)	You just find that she has removed herself from the drape, you tell her no, an hour has not finished yet (Midwife, #22)
**Feasibility**				
Availability of supplies<br><br>*(COM-B: Physical Opportunity)*	'I've never lacked it I don't know how they how much we have in stock but there is no moment I've seen that we are lacking' (Doctor, #14)	'Yes, for now we have it available' (Midwife, #03)	'Yes, we have [drapes]. So, we never went out of stock' (Midwife, #26)	'They are adequate, enough stock and in case they are out of stock we order, we have never been having a shortage of drapes' (Midwife, #23)
Barriers to taking vital signs<br><br>*(COM-B: Physical + Social Opportunity; Reflective Motivation)*	'When our BP machine has got no batteries and maybe even the cradle… maybe it has not been charged… forces us to go to another to another natal ward, or post-natal ward we each share that machine' (Midwife, #16)	'Sometimes following the effects of misoprostol, the woman will be shivering…therefore reading may be inaccurate, but you still do it because you want to know if there is a change in the system of a patient.' (Midwife, #01)	'it's a little bit challenging when babies need to be breastfed or when we have a perineal tears that need to be sutured. So, there's constantly two or three nurses busy, one will do the observations, one will attend to the perennial tears. So, I think most of our challenges when a patient is on the drape, and she needs to be sutured. So that's quite an uncomfortable position for her to be in.'(Midwife, #28)	'It is not done by hundred percent, I can't say that I check every 15minutes, sometimes you are too busy and there is a lot of complications so you end up checking once or twice, but you try to check before ending of that 1 hour even if you are very busy' (Midwife, #23)

Appendix Table 7: Qualitative themes related to each implementation outcomes for PPH management

This table presents data (themes and exemplar quotations) from the qualitative interviews with health workers conducted in intervention sites, about management of PPH. The table is organised under each implementation outcome of interest: fidelity, adoption, adaptation, acceptability, and feasibility. A summary version of this table is presented in Table 5 of the main manuscript.

Themes	Kenya	Nigeria	South Africa	Tanzania
**Fidelity, adoption, adaptation**				
Variation in when the MOTIVE bundle is triggered to manage a PPH	'As minimal as high as 300 mls or the yellow line that is 300 mis. But anything above 500 is with or without any vital signs, we will consider as PPH. So, we start the treatment at that point' (Doctor, #13)	'When blood loss reached 300 mls, we check the patient vitals, then we trigger the E-MOTIVE immediately' (Midwife, #01)<br><br>'When blood loss reached 500 mls, that's when PPH is diagnoses. So, still we trigger the bundle.' (Midwife, #07)	'You see that it's 500 mls of blood loss and more. And that you're gonna start the MOTIVE bundle' (Midwife, #27)	'Most of the time we use drape weight and vital signs, every 15 minutes we monitor the mother. So it means when you check on the drape, let's say she has bleed 300mils, if you check vital signs and there is one or more worrying sign either BP is low or pulse rate is high, so that shakes you and if you see the mother is still bleeding , the flow is either heavy or clot or whatever, if you check and see there something lets trigger the bundle before the mother go in to worsening…' (Midwife, #22)
Adherence to MOTIVE bundle for management of PPH	'As we take interventions, the other members start taking the vitals... some ...they implement the MOTIVE bundle by administering tranexamic acid, IV fluids, with.... saline and 10 international unit, oxytocin, ... flow very fast, and the other one take part in uterine massage' (Midwife, #10)	We give it closely together…. you have to do everything at the fastest time even before that 15 minutes as was set. at your fastest possible time to all the good things for the woman (Midwife, #12)	'We've been having quite a few patients that have been diagnosed as PPH. And we've been managing them according to the protocol' (Midwife, #30)	'You start by massaging the uterus, then you give oxytocin then you give tranexamic acid. And also, you do examination' (Midwife, #15
Deviations from, and additions to, MOTIVE bundle	'When I'm called, when I ask what has been given sometimes TXA hasn't been given, so I have to instruct them to give. Yeah. I don't know why' (Doctor, #14)	'I would not give her tranexamic acid…everybody come up administer the uterotonics … by the time I empty the bladder, massage the uterus, the bleeding may stop. So, some are more often than others' (Midwife, #8)	'We start off with 10 units of oxytocin, which we run into 100 mils of normal saline over five minutes. Then we give one gram of TXA also in 200 mils of saline after the oxytocin over 10 minutes and we start the patient on a 20 units of oxytocin maintenance at 125 mls an hour….If the bleeding is still not controlled…we repeat the TXA, what is 100gms in 200 mls and obviously we escalate to the doctor… decides whether we're going to give the patient cycotex or ergometrine' (Midwife, #30)	'Treatment given other than MOTIVE bundle maybe blood transfusion, if she has bled excessively, we must control blood then we check how much is it, we give blood transfusion if she is eligible … those are other treatments which we give for a mother with PPH (Midwife, #23)
**Acceptability**				
Improved outcomes for women<br><br>*(COM-B: Reflective Motivation)*	It has reduced the maternal mortality and morbidity. So, most of the times we have been able to capture it before it becomes detrimental to the mother's health (Doctor, #12)	'Yes, benefits because we have positive results' (Midwife, #01)	It has helped help everyone like us also as healthcare professionals to manage your PPH case efficiently. And also, for patients' sake, they also like <u>we're not missing out</u> on anything (Doctor, #32)	You [as a team] can manage PPH and save a mother not ending up in shock (Midwife, #22)
Empowerment of nurses and midwives<br><br>*(COM-B: Reflective Motivation + Social Opportunity)*	Our nursing staff is more empowered, is more empowered to deal PPH. So rarely, rarely they need us (Doctor, #13)	It has made me feel more professional (Midwife, #01)	Our nursing staff is empowered to start treatment and not wait for it. Because it before it's too late to actually start treatment (Doctor, #25)	-

Acceptance as part of clinical role and responsibilities<br><br>*(COM-B: Social Opportunity)*	It is part of my clinical roles. And also, it's part of my responsibility to administer as I detect the condition of the mother and then I implement this (Midwife, #11)	E-MOTIVE bundle is actually supposed to be research but any research that is impacting the outcome of patients... Yeah, it's part of my clinical role (Doctor, #08)	I think it's a very good way of expanding our role as midwives (Midwife, #28)	-
Impact on workload<br><br>*(COM-B: Reflective Motivation + Physical Opportunity)*	-	There is no increase in the workload (Midwife, #03)	There was like a lot of resistance because it was like an added workload on the staff. And I'm sure everybody knows not the hospital is like chaotic. (Midwife, #30)	The increase in responsibilities is one of job responsibilities, work has increased perhaps in the documents, in doing documentation (Midwife, #19)
Initial reluctance and adapting to something new<br><br>*(COM-B: Reflective Motivation)*	I think they [midwives]were finding because it's something new. So, to adapt was a bit different and difficult (Doctor, #32)	At first when we were not familiar it's kind of....you can easily forget it, but for now when we are used to it so it's no more difficult (Midwife, #05)	So, staff, were actually a bit hesitant at first, but once it's been implemented, it's like part of our routine now (Midwife, #30)	The first time it was difficult because I didn't understand it, but after I got the training …I felt it an easy way to help me when staying with the woman for the whole hour (Midwife, #18)
Self-efficacy and ease of delivering the bundle<br><br>*(COM-B: Reflective Motivation + Psychological capability)*	I'm confident because every so far, the period I've worked in labour ward as that MOTIVE bundle been key guide have seen it working It has never failed. It has never failed. So, you have that confidence in it (Midwife, #16)	There is not any difficulty because….we used to do all these things before the coming of MOTIVE bundle (Midwife, #03)	I feel confident in applying the MOTIVE bundle (Midwife, #27)	It's easy because you'll be able to detect it quickly, because if it didn't have measurement, it might be a lot of blood, but you don't know how many mls they are, so if there's a problem, you won't be able to detect it early (Midwife, #20)
Easy to remember and becoming automatic<br><br>*(COM-B: Automatic motivation + Psychological capability)*	It becomes a normal procedure for us. So as soon as you detected a patient with PPH, you just continue and give all this bundle (Midwife, #03)	Because in the beginning, you used to forget but it is now stuck in our brains. Okay. I don't think we ever forget (Midwife, #27)	They [Midwives] rely on the protocol so much; they stop to use common sense. And that kind of comes through with it, we, because we're just looking at blood. And although the protocols say we should look at clinical features as well, I find that people only look at the blood, and they forget to look at the patient (Doctor, #25)	It's simplified and it's put in a manner that you won't forget easily. It is more simplified, and it is straight, you know after you finish this, you go to that and that … meaning it has become like a routine (Midwife, #17)
Disciplinary action for not adhering to bundle<br><br>*(COM-B: Social opportunity)*	Now we don't do disciplinary action ... for those of us who maybe miss what we do is mentorship (Doctor, # 13)	Yes, we get punished if once we do not use the MOTIVE and there's a case of PPH (Midwife, #05)	Sister [name], she is very strict about following guidelines and protocols and stuff like that…. I would say you can be worried if you don't follow the bundle, and if know that it was a PPH case you didn't act upon it (Midwife, #29)	There are disciplinary actions it might happen, though here is very few, maybe if it happens the mother has PPH and management was not provided effectively, we normally meetings. We have ward meeting which involves MOI so we correct each other, that in this scenario mother would have ended up to 400mls but you let her reaches 1000mils and give her complications or why didn't you deliver this and that in that case (Midwife, #23)
Negative emotions<br><br>*(COM-B: Automatic Motivation)*	As a nurse the only stress you feel is you don't want any mother to have PPH" (Nurse, #11)	There is obviously there is fear of what if something happens and maybe we even it leads to morbidity or mortality because of that blood loss (Midwife, #02)	There's like a lot of emotions that go through, you're stressed out, you're scared, you don't know what outcome, you're trying to make sure that your management is going as fast as possible, so that you can help the patient out (Nurse, #30)	This bundle makes me feel confident, because I know that when use this out, the mother will not have any problems. I will be monitoring there, I check. I am not afraid at all; I am doing it without any fear (Midwife, #19)

**Feasibility**				
Good understanding of bundled approach to PPH management<br><br>*(COM-B: Psychological capability)*	The MOTIVE bundle is early detection, the PPH then M stands for massage of the uterus, O stand for special enough oxytocin and then I for IV fluids T for TXA. Then, E for escalation that is so when you have that E-MOTIVE in mind (Midwife, #11)	The MOTIVE bundle is a process of steps that we use in early detection of early detection and management of PPH. We do uterine massage like I said before, we give oxytocin injections, we give tranexamic acid, we give the set of IV fluids an also pass misoprostol and we escalate (Midwife, #02)	E-MOTIVE is basically the early detection and management of PPH in patients who were using medications that have been prescribed according to the E-MOTIVE protocol to manage. Yeah, so it's basically we measure the blood loss, and which treating the patients accordingly (Midwife, #30)	The MOTIVE bundle is a set of medicine and procedures put in place to help a mother who has delivered and having excessive bleeding. This bundle consists of the following including E-MOTIVE trolley, its where the whole bundle is carried, there is the drape that measure blood, there are the principles for early determination. to administer oxytocin, medicine that helps the uterus to contract and prevent bleeding, there is tranexamic acid that helps in blood clotting, there is misoprostol for uterus contraction, there are the fluids to help restore the patients vitals, then there is the monitoring charts that you fill to help in the monitoring of the patient to determine the next step to take, we also have the VSA machine that helps determine the mother's progression blood pumping, blood and pulse pressure and it also set us with time frame to attend the mother and past the time frame I should take a different step. All set of principles and the medication and the whole trolley setup, the bundle is complete, and it gives us the time frame (Doctor, #17)
Staff shortages and workforce challenges<br><br>*(COM-B: Social Opportunity)*	Challenges is the thing... just about the staffing. So, it's kind of it gets us disorganised because if the other person that you need to call for help is already on another person too ...... she also needs help (Midwife, Nigeria #2)	We have very short staff here (Midwife, #27)	Sometimes there are some issues with some of the agency sisters that don't work here on a usual basis. They're just come and do part time shifts. So, they don't usually know the protocol.	That is a challenge, staffs are not adequate in labour ward sometimes you might be alone in the shift (Midwife, #23)
Need for multiple staff to deliver the bundle<br><br>*(COM-B: Social Opportunity + Reflective motivation)*	I have implemented the bundle alone. And it was a bit tricky because have to be so fast to implement (Midwife, #11)	I think it's easier to deliver it as a team (Midwife, #02)	You need a team. You can't do anything alone. You can start something alone (Midwife, #28)	It is easy when you are working as a team, but it is difficult when you are doing individually (Midwife, #18)
Improved team working and communication<br><br>*(COM-B: Social Opportunity)*	It's really improved. I think it's easier now to work as a team because when the bell rings, everybody knows you're supposed to, to be there and assist and even to share us with managing a patient with PPH (Doctor, #13)	Everybody comes to this including the doctors. If the patient already doesn't have an IV line they come to set it; another person is getting the fluid, so there's actually teamwork - well coordinated (Midwife, #02	With me being the nurse, so I can't put up the drape. So, I need to call the midwife and with mixing the medication. So, we've worked well together as a team (Nurse, #26)	Communication has changed greatly…. for now, shouting has been synchronised, once you shout the others understand what is happening why is happening, so the respond quickly as compared to the past before someone was aware not trained (Midwife, #17)
Peer support and encouragement<br>*(COM-B: Social Opportunity)*	Someone is going to be there and is going to remind you, you have not applied the drape It's time to apply the drape, please put it on (Midwife, #9	The matron in charge also tries to say that we administer all the bundle to help the women (Midwife, #05)	Your colleagues, like, motivate you and they cheer you on, like, you know, you did a good job, the patient is stable, well done, you know, things like that actually boosts our teams<br>From our manager from the research sister, (name), the interns have been like	

			very hands on with us as well (Midwife, #30)	
Involvement of research midwife<br>*(COM-B: Social Opportunity)*	That is a big challenge comes a problem because right now, if my colleague has a PPH and this is a general hospital and when we are all on duty three of us and we have 2 midwives and one E-MOTIVE staff one right now…… we have eight delivery beds. So, to be divided…two of us or three of us, it's very difficult. (Midwife, #16)	-	-	They are availabe. Including me who have been trained recently, trainers and my fellow staffs who have studied together with E-MOTIVE staffs are always ready and available whenever we have a problem especially with our trolley, they know where to access those equipments quicker than someone who has not been trained, because they know where they are. (Doctor, #2)
Availability of drugs<br><br>*(COM-B: Physical Opportunity)*	'Oxytocin we have but tranexamic acid…. most likely to be out of stock…sometimes we didn't have even normal saline' (Midwife, #15)	'It is always readily available. The oxytocin or the misoprostol and the rest that we need, the IV fluids, the catheter, all the things that we need yeah, readily available' (Midwife, #02)<br><br>'Especially the oxytocin sometimes, because the quality of it is not as like the branded one that we used to have from the MOTIVE bundle' (Midwife, #03)	-	'You have found a mother with PPH but there is no tranexamic acid… So that was a big challenge' (#18, midwife)<br><br>'Sometimes the facility lacks IV fluids, so we use those special fluids provided by E-MOTIVE' (Midwife, #24)
Bed shortages<br>*(COM-B: Physical Opportunity)*	-	-	It's so busy, our turnover is so high that sometimes, you know, there's no beds. Yeah, that one hour, the patients on the bench, whereas we needed the bed for someone else (Midwife, #31)	'The challenges maybe there are few labour beds, there are only three, and there are a lot of mothers who come for delivery' (Midwife, #19)

Appendix Table 8: Qualitative themes related to the implementation strategies

This table presents data (themes and exemplar quotations) from the qualitative interviews with health workers conducted in intervention sites, about the E-MOTIVE implementation strategies. The table is organised under each implementation outcome of interest: fidelity, adoption, adaptation, acceptability, and feasibility.

Theme	Implementation outcome	Kenya	Nigeria	South Africa	Tanzania
**Training**					
Training delivery and uptake	Fidelity, Adoption	'We have our E-MOTIVE staff with us, they have been doing it [training]' (Midwife, #10)	'They [E-MOIVE staff] came here and gave us intensive training…we did practical sessions on it, we were given scenarios on how to respond to certain situations, so we did it practically and written also' (Midwife, #08)	'Everyone has been trained, everyone knows what to do' (Midwife, #26)<br><br>We did attend a training. That was a ninety-minute training, if I'm not mistaken, we had like, the theoretical parts. And obviously, the practical parts of managing the bundle. Yeah, with the scenarios of using the bundle to manage the patient (Midwife, #30)	'Yes, [training] from the facility in charge, and the other special nurse for E-MOTIVE (Midwife, #24)'
Improved confidence, knowledge and skills because of training	Acceptability	'Things move very fast and very smoothly. And I think it's partly it's because of the training.' (Midwife, #08)	'I think I can properly manage PPH wherever I find myself because of the training' (Midwife, #02)	'Very, very helpful, because the more you did the roles, the more you become alert for the next time you have a patient….you remember things better, because you're doing it all the time. Repeatedly.' (Midwife, #28)	'The first time, it was difficult, because I didn't understand it, but after I got the training….I felt it an easy way to help me when staying with the woman for the whole hour.' (Midwife, #18)
Training improved communication amongst teams	Acceptability	'We are having more, like we speak one language….if there is a PPH, I only ask if the bundle has been activated. Nurses tell me yes, we've done this and this in the bundle. And I understand.' (Doctor, #14)	-	-	'It has been improved because there were training that was provided. When we are at work, you must do every possible method to be able to interact well with your partner.' (Midwife, #22)
Lack of time as a barrier to attending training	Feasibility	'…the only change here would be, of course, it consumes times for the staff' (Doctor, #13)	'I just haven't had the time. The others have done so.' (Doctor, #07)	-	About three times, we practice several times whenever we have time because we don't have too much time to cover everything (Doctor, #21)
**PPH trolley or carry case**					
Availability and use of PPH trolley/carry case	Fidelity	'Yes, the carry case, is there…honestly, I have not used it personally. So, I will not comment much about it. But we use the trolley all the time.' (Doctor, #13)	'Yes, we have the E-Motive trolley always nearby' (Midwife, #02)<br><br>'You just move the trolley or you shout for someone to bring the trolley for you' (Doctor, #07)	There's a fridge with the oxytocin in and there's a [cupboard] where the other medications are kept... Also, the fluids are in another storeroom, but it's close by so again (Nurse, #26)	'For the E-MOTIVE champions, for example there in charge, they are the in charge of managing that, that MOTIVE bundle which is an emergency kit in order to make sure everything is complete and in case if someone has used should replace (Midwife, #20)'
Benefits of having a PPH trolley/ carry case	Acceptability	For us having shortage of staff it is helpful because everything is organized, it is different if you are three or four even if the things are not organized others will help to find, if you are alone, you just open the carry case and take and later you replace used materials. It is very helpful (Midwife, #21)	The helpful thing is that you don't need to be running and descending, go to the fridge go because everything is stocked there on the trolley. So you just put your things there is nothing.' (Nurse, #01)	Everything is stored in that box. So, it's like not us running helter-skelter trying to locate supplies. We have everything at that moment to start management faster (Midwife, #30)	The PPH trolley helps a lot because it is near our beds, so when it happens, you find it easy to pick it up and start serving the mother. but if they could be far, it could be a problem you must run and take them from somewhere, so the proximity of the trolley helps us a lot (Midwife, #19)

Consistent stocking of PPH trolley/case	Feasibility	'Currently we check on everything, every morning, so it is usually stocked.' (Doctor, #12)	'It is always stocked' Midwife, #02)	'What I usually do when we are done with it with a box, I will just restock what been taken out. And then every day in the morning, the box is checked that is all the things are there.' (Nurse, #02)	To replace we had a meeting and decided to ask mothers to purchase so that the trolley doesn't have shortage, currently we have improved in managing that part (Midwife, #21)
Insufficient PPH trolleys/cases	Feasibility	-	'We have only one trolley from E-MOTIVE. And sometimes, depending on the number of deliveries, I think the challenge we're facing, because two different women might be having a PPH and you can't just roll it, the trolley to one person and ignore the other' (Midwife, 06)	-	-
**PPH Champions**					
Champions appointed	Fidelity	'Yes….we have four [champions].' (Nurse, #01)	'We have three E-MOTIVE champions' (Midwife, #10)	'Yes, 2 nurses (sisters) and doctor' (Nurse, #29)	'There are champions, I know three of them and I know three of them and there is also a leader who is in charge internally, apart from the champions like [name] and who was in-charge of the E-MOTIVE staff in here so for the ones who come here from other places are few (Midwife, #18)'
Fulfilling the role of a champion	Fidelity	'We do the training….we've done continuing medical educations, you've done the practice sessions. Generally, it's just describe it as mentorship' (Doctor, #13)	'They normally come around the ward, whether they're on duty or not, to observe whether the drapes are being used and how they are being used, whether they are being used correctly….and also make sure the PPH drawer or cabinet is fully stocked' (Doctor, #09)	'If there's any problems or concerns or something... recently I asked Dr. [name], just tell me what, what's the newest thing now? Do we do this? How does this fit in? I mean, [inaudible and unclear] championing in that regard.' (Nurse, #01)	'Their responsibilities are to supervise us, ensuring the E-MOTIVE services are given, ensuring equipment are available and ensuring the team works as per the training' (Doctor, #01)
Tension between champions and other ward staff	Acceptability	-	'The champion may take so much power trying to correct people and so it's not everybody that is amenable to correction so people may be hurt.' (Doctor, #09)	-	-
Champions are helpful	Acceptability	'The [champions] are helpful, especially the nurses, they are usually on the ground very helpful….train us occasionally, reminds us to implement the bundle.' (Doctor, #14)	'She's always there giving you a listening ear. Because when we started out the bundle…she was telling all the team that was involved serving us in lighting or before we even went for training and enlightened us, so she has done very well.' (Midwife, #12)	'I must day that they are very supportive of us as staff on the floor. So, they will always when we tell them. there is a patient who has been diagnosed with PPH they'll always ask 'have you sorted?'... or how far are you now with the bundle? Can I help you? (Midwife, #28)	They stand as role models for us to show us that when you achieve this, you have to do his… they did their great job to educate and teach practically that you are supposed to do this and this in order to reach our goals (Midwife, #18)
**Audit and feedback**					
Audit newsletter displayed or discussed with other staff	Fidelity	'Yes. We've had discussions with the consultant, though, mostly virtual, like on WhatsApp comment after they posted the [audit report] report. And then I think, during the training, we also discussed one of the reports' (Doctor, #06)	'There is no feedback, any feedback' (Midwife, #04)<br><br>'We receive feedback monthly, through the champions' [Nurse, #06]	'I haven't seen the newsletter which comes out' (Doctor, #32)<br><br>sister [name] gives us feedback or she gets emails, that stuff that she needs to report back to us (Midwife, #26)	'Most of the time newsletter come monthly, and every month we have ward meeting so we discuss during that time' (midwife, #23)

Feedback is motivating	Acceptability	'It has been helping us to see how we are doing for every month, after practicing E-MOTIVE, so we can see the gap to se where we have done well.' (Midwife, '10)	'Whenever I received the newsletter and see that there is improvement, so it makes me put more effort in' (Midwife, #04)	'It is nice to know that we're picking up so many cases like before, I don't think there was proper stats of like, you know, and I think also, they you probably pick up certain amount of cases, but now we are picking up more because of the drapes. Yeah, because of the proper measurement' (Midwife, #26)	'It [feedback] encourages them [staff] if you are told that you have done seventy percent but you have failed thirty percent in delivery of TXA or oxytocin you are delivering but not effectively so you will try your best in the next season to move from seventy to ninety so you will put more efforts because they know they are graded in that intervention so they have to improve and impress' (Doctor, #21)

Appendix Table 9: Qualitative themes related to contamination

This table presents data (themes and exemplar quotations) from the qualitative interviews with health workers conducted in intervention and control sites, about potential threats of contamination between the intervention and control trial arms. The table is organised according to the potential contamination threat.

Theme label	Kenya	Nigeria	South Africa	Tanzania
Participation in other research studies	We had a study on sepsis .... Yes, we also have an ongoing study on preeclampsia and hypertensive disease in pregnancy…. Generally, they…do not impact on the E-MOTIVE study because the issues being handled with the study in those studies are slightly different from PPH (Doctor, #13)	Honestly, I'm not aware of that. It's just E-MOTIVE is the first I've witnessed, and maybe that's because I must have not been here for long. I've only stayed one year in labour ward and I met E-MOTIVE there. I have not heard of any other (Midwife, #02)	This is the main study which we had in obstetrics recently (Doctor, #32)	The first one was [another PPH related study] and we were measuring by using clothes, (Midwife, #22)<br><br>Since I work here, I haven't seen anything than E-MOTIVE (Midwife, #23)
Guidelines and other quality improvement initiatives	'Maybe the bell. It was a team's idea actually, was borrowed from one of the sessions we had as one of the champions, we had a champion session at Mombasa, whereby we borrowed that idea from other... one of the facilities. So, I think it's a good thing. It's improved teamwork, because once the bell rings, people come around, and people know that it's an emergency. So, it's a good idea for us' (Doctor, #13)	We have the National Guideline for management of PPH which has come out the one for 2022; this one was applied in between the trial by the national government (Midwife, #11)	None reported	It is only the ministry's guide and what we learnt in college some of the people who have attended seminars otherwise and it's just those guides (Doctor, #17)<br><br>Other sources maybe we have a biosafety group they are just reminding us how to fight these PPH, it is same with what we are doing (Midwife, #19)
Sharing with others	'Yes, I have. My fellow doctors….we will be talking…I had colleagues asked me this question on PPH ..this and this happened. And then I'll suggest to them to try to use the bundle. Yes. So that... they tell me it's actually it's It sounds easy. Those who have applied it, find it very easy to use. So, I think it should be our recommend that maybe... it should be ... I mean, if it's spread all over so that everybody uses it, I think it will improve the outcomes for mothers who get PPH' (Doctor, #13)	Tell my friend …. we are doing so that was something that we're discussing. I say we triggered bundle, then she asked what's treatment bundle (Midwife, #01)	'Definitely with other institutes type private and government institutes as well. So, there has been like a lot of people that have like what, you know, during our random talks, when we talk about work and stuff, we mentioned things like the bundle and management of patients and stuff like that. And they have taken a keen interest, actually others I know, that are working in other hospitals have mentioned how, you know, what a great initiative it is. And, you know, they actually hope that they could have something like that in the institute's because, I mean, it makes management so much more effective for the patient and at the end of the day, the patient outcome that we reprioritize (Midwife, #3)'	'Maybe in another hospital, when you are talking on the phone and you are discussing, we have an E-MOTIVE project, where you directed to do things, we given bundles, and give TXA to prevent PPH (Midwife, #19)'
Changes to practice in control sites: estimating blood loss using uncalibrated drape	'Since the start of the study that [drape] is what was used. Before it was estimation, estimation, no weighing no nothing. Just to estimating the amount of blood the women have lost...we are using the drape to every patient deliver...Since the beginning of this study I would say about 100% of use of these drape for the patients.' (Doctor, #11)	'Actually now, with this E-MOTIVE, the drape that we usually put under women when they deliver, it helps us to detect PPH especially with the level of ...volume of blood in the drape... [we use it] For every patient that comes to the delivery.' (Nurse, #10)	"We use drapes... we are using the drapes at the moment to estimate the blood' (Nurse, #9)	'Currently what we do is first, as soon as a mother deliver we have those E-MOTIVE drapes, so when the child comes out and before the placenta come out, we put those drape which inside it has a soft gauze, full soft not those that are partial soft.' (Doctor, #12)

Appendix Table 10: Control survey data: calibrated drape and bundle use (contamination)

This table presents data (frequencies and percentages) from the cross-sectional survey with health workers conducted in control sites, about potential threats of contamination in the control sites regarding methods of PPH detection and management.

	Kenya n=130	Nigeria n=158	South Africa n=53	Tanzania n=87	Total n=428	p-value†	Implementation outcome
**Methods used for PPH detection**							Low
Drape[1]	123 (94·6%)	148 (93·7%)	51 (96·2%)	81 (93·1%)	403 (94·2%)	0·87	contamination
Visual estimation	76 (58·5%)	120 (75·9%)	27 (50·9%)	53 (60·9%)	276 (64·5%)	0·0012	
Vital signs	100 (76·9%)	113 (71·5%)	32 (60·4%)	50 (57·5%)	295 (68·9%)	0·0092	
Uterine tone and size	76 (58·5%)	88 (55·7%)	24 (45·3%)	37 (42·5%)	225 (52·6%)	0·070	
Counting or weighing blood-soaked swabs	109 (83·8%)	117 (74·1%)	21 (39·6%)	47 (54·0%)	294 (68·7%)	<0·0001	
**How often a drape was used**							Low
Often/always	121 (93·1%)	137 (86·7%)	50 (94·3%)	82 (94·3%)	390 (91·1%)	0·23	contamination
Sometimes	6 (4·6%)	11 (7·0%)	1 (1·9%)	1 (1·1%)	19 (4·4%)		
Never/rarely	3 (2·3%)	10 (6·3%)	2 (3·8%)	4 (4·6%)	19 (4·4%)		
**Duration of drape used (n=426)**							Low
30 min	18 (14·0%)	25 (15·9%)	6 (11·3%)	6 (6·9%)	55 (12·9%)	0·23	contamination
≥60 min	111 (86·0%)	132 (84·1%)	47 (88·7%)	81 (93·1%)	371 (87·1%)		
**Liked using a drape for PPH detection**	128 (98·5%)	154 (97·5%)	52 (98·1%)	84 (96·6%)	418 (97·7%)	0·81	
**Effective methods for PPH detection**							Low
Using tools (e.g., kidney basin, swabs, linen)	97 (74·6%)	134 (84·8%)	29 (54·7%)	56 (64·4%)	316 (73·8%)	<0·0001	contamination
Visual estimation	59 (45·4%)	99 (62·7%)	31 (58·5%)	51 (58·6%)	240 (56·1%)	0·027	
Vital signs	100 (76·9%)	123 (77·8%)	35 (66·0%)	58 (66·7%)	316 (73·8%)	0·11	
Uterine tone and size	75 (57·7%)	80 (50·6%)	27 (50·9%)	41 (47·1%)	223 (52·1%)	0·45	
Counting and weighing blood-soaked swabs	122 (93·8%)	136 (86·1%)	34 (64·2%)	56 (64·4%)	348 (81·3%)	<0·0001	
**Methods used to manage PPH**							Low
Uterine massage	120 (92·3%)	149 (94·3%)	52 (98·1%)	77 (88·5%)	398 (93·0%)	0·15	contamination
Monitor blood pressure & pulse rate	103 (79·2%)	130 (82·3%)	49 (92·5%)	76 (87·4%)	358 (83·6%)	0·11	
Perform examination of cause of bleeding	122 (93·8%)	142 (89·9%)	45 (84·9%)	78 (89·7%)	387 (90·4%)	0·29	
Administer intravenous fluids	115 (88·5%)	133 (84·2%)	49 (92·5%)	83 (95·4%)	380 (88·8%)	0·048	
Administer tranexamic acid	115 (88·5%)	112 (70·9%)	41 (77·4%)	72 (82·8%)	340 (79·4%)	0·0025	
Administered uterotonics	124 (95.4%)	147 (93.0%)	50 (94.3%)	74 (85.1%)	395 (92.3%)	0.035	
**Availability of uterotonics**							Low
Often/always	116 (89·2%)	154 (97·5%)	51 (96·2%)	80 (92·0%)	401 (93·7%)	0·022	contamination
Sometimes	14 (10·8%)	4 (2·5%)	2 (3·8%)	6 (6·9%)	26 (6·1%)		
Never/rarely	0 (0·0%)	0 (0·0%)	0 (0·0%)	1 (1·1%)	1 (0·2%)		
**Availability of tranexamic acid**							Low
Often/always	88 (67·7%)	123 (77·8%)	47 (88·7%)	54 (62·1%)	312 (72·9%)	0·0017	contamination
Sometimes	37 (28·5%)	29 (18·4%)	4 (7·5%)	23 (26·4%)	93 (21·7%)		

	Kenya n=130	Nigeria n=158	South Africa n=53	Tanzania n=87	Total n=428	p-value†	Implementation outcome
Never/rarely	5 (3·8%)	6 (3·8%)	2 (3·8%)	10 (11·5%)	23 (5·4%)		
**Availability of IV fluids**							Low contamination
Often/always	113 (86·9%)	155 (98·1%)	53 (100·0%)	78 (89·7%)	399 (93·2%)	0·0002	
Sometimes	17 (13·1%)	3 (1·9%)	0 (0·0%)	9 (10·3%)	29 (6·8%)		
Never/rarely	0 (0·0%)	0 (0·0%)	0 (0·0%)	0 (0·0%)	0 (0·0%)		
**Equipment**							Low contamination
Often/always	102 (78·5%)	153 (96·8%)	48 (90·6%)	79 (90·8%)	382 (89·3%)	<0·0001	
Sometimes	23 (17·7%)	5 (3·2%)	2 (3·8%)	8 (9·2%)	38 (8·9%)		
Never/rarely	5 (3·8%)	0 (0·0%)	3 (5·7%)	0 (0·0%)	8 (1·9%)		

†Fisher's exact and chi-squared tests were used. [1] Control sites used uncalibrated drapes for blood loss collection as part of the trial outcome measurement.

Appendix Study Instrument 1: Qualitative Interview Guide (*Control Sites*)

Note to the interviewer: To recap, the aim of this interview is to find out:

- What is currently done to detect and to manage a PPH
- What is likely to influence PPH practice

IMPORTANT: Remember this is the control site so they will not have a calibrated drape or have received any training about using E-MOTIVE. Please avoid mentioning either the calibrated drape or the MOTIVE bundle to participants.

Instructions and suggestions for the interview

a. Please ask all questions in **BOLD (or have responses to these questions)**
b. The questions *in italics* are follow up prompts to help further elaborate participants' responses. Please wait before asking the *prompt questions* until after the participants has responded to the question in bold first.
c. Based on the person's responses, don't ask prompts that have already been discussed in the response or prompts that don't make sense in the context of the participants' response
d. Use the *prompt questions* to get more detailed information about how the drape is being used and what might influence its use. Also, their views and experiences of what influences current PPH management.

Interviewer (please read to the participant at start of interview):

Thank you very much for taking the time to speak to me today. My name is [interviewer name], and I am one of the E-MOTIVE study team members. Before we begin, can I please confirm that you have received a copy of the study information sheet and consent form?

As a reminder, this study aims to explore how postpartum haemorrhage (PPH) is currently detected and managed in hospitals such as this. We are interested in hearing your views and experiences about what currently happens in practice, and what factors influence how PPH is detected and managed

So please give your honest feedback, there are no right or wrong answers. Everything you say will be treated confidentially and will not be shared with any of your colleagues, or anyone outside of the EMOTIVE study team. You are free to answer in as much or as little detail as you wish, to skip over any questions you do not wish to answer, and to pause or stop the interview at any time if needed.

This interview will take approximately one hour- depending on how much you have to say. Can I please check you are free now to talk for this amount of time?

I would also like to please record our conversation- so that I can capture your responses accurately, and so that I can listen to you rather than take many notes. Can I confirm you are happy for me to start recording?

Section 1 - Background

I would like to start with understanding a little about your role in your current job.

1. **What is your current position?**
2. **How long have you been a [nurse, midwife, doctor]?**
3. **How long have you worked at this hospital?**

Section 2 - PPH detection

I would like to start by asking you some questions about PPH detection.

4. **Please describe what happened when you recently detected or assisted in detecting a PPH?**
   a) **How was the PPH detected?**
   b) **Were any tools used? If so, which ones?**
   c) **Is this what typically happens?**
5. **What do you use to estimate blood loss?**

If participant mentions that they use a drape, ask:

   a) **To what extent do you use the drape to estimate blood loss and decide what needs to be done next?**

6. **To what extent, does determining the blood loss inform what needs to be done next?**
7. **In order to detect a woman's response to blood loss, do you take vital signs? which ones?**
   - If yes, **How often are vital signs taken and for how long?**
   - If no, **Why not?**
8. **Are there any challenges with taking vital signs?**
9. **What typically happens in response to a change in the vital signs?**

Section 3 - PPH management

Now, I'd like to ask you some questions about PPH management.

10. **Please describe what happened when you recently managed or assisted in the care for a woman with a PPH?**
    Prompts:
    a) **What treatments were given to manage that PPH?**
    b) **In what sequence were the treatments given?**
    c) **Did you encounter any issues or challenges with giving the treatments?**
    If yes, **what were they?**
11. **Are there any other treatments routinely given to manage a PPH?**
12. **Do you ever wait to see if one treatment works before giving another treatment?**
    If yes, **After which treatments (if any) would you typically wait and see?**
13. **How easy or difficult is it to manage a PPH?**
    a) **Are there any treatments you feel more or less confident doing?**
    b) **Are any treatments more or less likely to be forgotten?**
14. **Do you have everything you need to manage a PPH?**
    Prompts for resources: time, staff, equipment, drugs information, training
    a) **What happens when what you need is not available or not working?**
15. **Do you think managing a PPH is part of your clinical role?**
16. **To what extent, do you think you have the knowledge and skills to manage a PPH?**

If not, **what would you like to receive training on?**

17. **Have you received any training on PPH since the training at medical/ midwifery/nursing school?**

    If yes,

    a) **When was the training?**
    b) **Where was the training? (internal or external)**
    c) **What did the training cover?**
    d) **How was it delivered? (in classroom or online)**
    e) **Has the training been helpful or unhelpful?**

18. **Do you use any clinical guidelines or protocols for detecting and managing a PHH? If so which ones?**

    If yes, **To what extent do you refer to them to inform your PPH practice?**

19. **To what extent do you receive any information or feedback about your performance of managing PPHs?**
20. **Do you think managing a PPH is too much for one person to do?**
21. **To what extent do you work as a team to manage a PPH?**

    a) **Do you think you work well as a team? Why or why not?**

    Prompts: Does anything get in the way of team working? What would help to support team working?

22. **Are there any issues with communication within teams?**

    If yes, **What are the issues?**

23. **To what extent do work colleagues influence the management of a PPH?**
24. **To what extent do women experiencing a PPH influence the management of a PPH?**
25. **To what extent do the women's relatives influence the management of a PPH?**
26. **How does managing a PPH made you feel?**

    Prompts: stress; anxious; worried or no emotional response

27. **To what extent does disciplinary action being taken on staff or on the hospital influence the detection and management of PPH in this facility?**

    a) **How does this impact on what you have told me about how staff do your job?**

Section 4 – Wrapping up

28. **Is there anything else that has been introduced in this ward or hospital that might have influenced PPH detection or PPH management in the last 6 months?**

    Prompts: any other initiatives, e.g., national guidelines, quality improvement initiatives

29. **Before the E-MOTIVE project was introduced here or since it started, are you aware of any other research studies taking places in your hospital related to maternal health or PPH?**

    If yes**, Do you feel that it has impacted on PPH practice?**

30. **Have you discussed the E-MOTIVE project with any colleagues from other hospitals at all?**
31. **Is there anything else about the E-MOTIVE project that you would like to share with me that's not been covered in the discussion already?**

These are all of the questions I have for you today, thank you so much for your time.

Appendix Study Instrument 2: Qualitative Interview Guide (*Intervention Sites*)

Note to the interviewer: To recap, the aim of this interview is to:

- Find out if the E-MOTIVE bundle is being used as intended (i.e., has a 'bundled' approach to PPH detection and management been integrated into current PPH practice?)
- What might influence its use?

We also want to **understand** whether participants think it is feasible and acceptable to use the E-Motive bundle as part of PPH detection and management, whether:

- Have any modifications occurred (i.e., is anything being done differently to the E-MOTIVE training)
- What they think are the consequences and impact of using the bundle.

Instructions and suggestions for interview:

a. Please ask all questions in **BOLD (or have responses to these questions)**
b. The questions *in italics* are follow up prompts to help further elaborate participants' responses. Please wait before asking the *prompt questions* until after the participants has responded to the question in bold first.
e. Based on the person's responses, don't ask prompts that have already been discussed in the response or prompts that don't make sense in the context of the participants' response
f. Use the *prompt questions* to get more detailed information about how the E-MOTIVE bundle and PPH kit/trolley is being used and what might influence its use. Also, their views and experiences of the E-MOTIVE training and champions, receiving audit and feedback

Interviewer (please read to participants at start of interview):

Thank you very much for taking the time to speak to me today. My name is [interviewer name], and I am one of the E-MOTIVE study team members. Before we begin, can I please confirm that you have received a copy of the study information sheet and consent form?

As a reminder, this study aims to explore your experiences and views of using the EMOTIVE bundle to care for women with postpartum haemorrhage. We are also interested in understanding your experiences and views associated supporting activities.

Your responses will help us understand how the E-MOTIVE bundle and supporting interventions are being used in practice, any challenge or problems and how we improve it. So please give your honest feedback, there are no right or wrong answers. Everything you say will be treated confidentially and will not be shared with any of your colleagues, or anyone outside of the EMOTIVE study team. You are free to answer in as much or as little detail as you wish, to skip over any questions you do not wish to answer, and to pause or stop the interview at any time if needed.

This interview will take approximately one hour- depending on how much you have to say. Can I please check you are free now to talk for this amount of time?

I would also like to please record our conversation- so that I can capture your responses accurately, and so that I can listen to you rather than take many notes. Can I confirm you are happy for me to start recording?

Section 1 - Background

I would like to start with understanding a little about your role in your current job.

32. What is your current position?

33. How long have you been a [nurse, midwife, doctor]?

34. How long have you worked at this hospital?

Section 2 - PPH detection

I would like to now ask you some questions about PPH detection.

35. Please describe what happened when you recently detected or assisted in the care for a woman with a PPH?

   a) How was the PPH detected in that case?

   b) Is this what typically happens?

36. What do you use to estimate blood loss?

If they do not mention a drape in response to Qs 4-5, ask:

37. Do you ever use a drape to determine blood loss?

If yes, ask questions in Column A of table

If no, ask questions in Column B of table

A - Drape is used	B - Drape is not used
**38. What does the drape look like? Are there any marks or calibrations on it?**<br>**39. How and when is the drape applied?**<br>**40. How does measuring the blood loss in the calibrated drape inform what needs to be done next?**<br>Prompts:<br>**a) What typically happens when blood loss reaches 300mls?**<br>**b) What typically happens when blood loss reaches 500mls?**<br>**41. How easy or difficult is it to use the calibrated drape?**<br>**42. How has using the calibrated drape impacted on PPH detection compared to how detection was done before?**<br>**43. Are calibrated drapes readily available in the ward?**<br>If no, **why not?**<br>**a) Do you have enough of them?**<br>**44. How do women react to having a calibrated drape placed underneath them?**<br>**45. Do your colleagues and/or peers influence your use the calibrated drape?**<br>If yes, **how?**<br>**46. Do you like using the calibrated drape?**<br>If yes**, why?** If no, **why not?**<br>Now, ask Question 16	**1. Can you take me through any reasons why the drape is not being used?**<br>**2. Is anything else being used instead of the drape to estimate blood loss?**<br>Now ask Question 16

47. In order to detect a woman's response to blood loss, do you take vital signs? Which ones?

If yes, **How often are vital signs taken and for how long?**

If no, **Why not?**

48. Do you ever encounter any problems or challenges when taking vital signs?

If yes, **can you take me through these or give me an example?**

49. **What typically happens in response to a change in the vital signs?**

Section 3 - PPH management

Now, I'd like to ask you some questions about PPH management.

50. **Please describe what happened when you recently managed or assisted in the care for a woman with a PPH?**
    Prompts:
    d) **What treatments are given to manage that PPH?**
    e) **In what sequence were the treatments given?**
    f) **Is this what usually happens when treating a PPH?**
    g) **Did you encounter any issues or challenges with giving the treatments?**
    If yes, **what were they? Can you give me an example?**
51. **Do you have everything you need to deliver the E-MOTIVE bundle?**
    Prompts for resources: time, staff, equipment
52. **Do you have a PPH trolley, PPH carry care or PPH box in the ward?**
    If yes,
    a) **Have you experienced any challenges to accessing and using the trolley/carry case/box?**
    b) **Have there been any issues with the trolley being kept stocked?**
53. **Are PPH drugs and equipment kept in any other places?**
54. **For you, what are the helpful and unhelpful things about having a PPH trolley/carry case/box?**
55. **Have any changes been made to the PPH trolley/carry case/box compared to how it was set-up when first introduced into the ward?**
56. **Do you think your workload has changed at all since the E-MOTIVE bundle was introduced?**
    Prompts:
    a) **To what extent is delivering the E-MOTIVE bundle is too much for one person to do?**
    b) **To what extent is delivering the E-MOTIVE bundle is too much for a team to do?**
57. **Are there any benefits of using the E-MOTIVE bundle?**
58. **Are there any disadvantages of using the E-MOTIVE bundle?**
59. **More generally, how often are uterine <u>M</u>assage, <u>O</u>xytocin, <u>T</u>XA, <u>IV</u> fluids, and <u>E</u>xamination & Escalation, usually given as part of managing a PPH?**
    a) **Are any done less often than others? Why?**
60. **To what extent are these components given closely together?**
    a) **Do you ever wait to see if one treatment works before giving another treatment?**
    If yes, **After which treatments (if any) would you typically wait and see?**
61. **Are there any other treatments routinely given to manage a PPH?**

Section 4 - Influences on uptake of E-MOTIVE bundle

I would now like to ask some questions about using the E-MOTIVE bundle to detect and manage a PPH.

62. **Can you briefly take me through your understanding of the E-MOTIVE bundle?**
    If no, **please explain why?**
63. **How easy or difficult is it to use the E-MOTIVE bundle?**
64. **How easy or difficult is it to remember to deliver the E-MOTIVE bundle?**
    a) **Are there any steps that you are more likely to forget?**
65. **Do you think using the E-MOTIVE bundle is part of your clinical roles?**
66. **Do you know about any guidelines or posters on how to detect and manage a PPH?**
    If yes, **To what extent do you refer to them to inform your PPH practice?**
67. **Have you received training on how to use the E-MOTIVE bundle?** If no, **Why?**
    If yes, ask, if not mentioned

a) ***Who provided it?***
b) ***What was the duration of the training?***
c) ***Who attended the training?***

68. **After the initial training, have you participated in any additional practice sessions?**
If yes, **What are the helpful and unhelpful things about the practice sessions**?
If no, **Why have you not participated in any practice sessions**?
69. **To what extent, do you feel adequately trained to deliver the E-MOTIVE bundle?**
If not**, What else is needed?**
70. **Have you recently received any other PPH education or training other than the E-MOTIVE training?**
71. **To what extent has team-work and communication within the changed since using the E-MOTIVE bundle?**
72. **To what extent do work colleagues influence using the E-MOTIVE bundle?**
73. **To what extent do women experiencing a PPH and their relatives influence use of E-MOTIVE bundle?**
74. **How has using the E-MOTIVE bundle made you feel?**
Prompts: (more/less stress; more/less anxiety; more/less worries)
75. **Do have any concerns about disciplinary actions resulting from <u>not</u> using the E-MOTIVE bundle to detect and to manage a PPH?**
76. **To what extent, would you plan to use the E-MOTIVE bundle for all PPHs**?
77. **How confident are you that the E-MOTIVE bundle can solve any problems associated with PPH?**
78. **Do you think that using the E-MOTIVE bundle has made a difference to PPH practice and outcomes?**

<u>Section 5 – E-MOTIVE Champion</u>

I would now like to move on to ask you about the E-MOTIVE champions.

79. **Do you have E-MOTIVE champions at your site?**
If yes, **How many? What is their clinical role/professions?**
80. **How have champions supported staff, what do they do?**
81. **For you, what are the helpful and unhelpful things about having a E-MOTIVE champion?**

<u>Section 6 – Audit and Feedback</u>

I would like to move on to ask you about any feedback shared with sites about using the E-MOTIVE bundle.

82. **To what extent do you receive any feedback or information about your team's use of the E-MOTIVE bundle?**
Prompts: **Have you seen or received the E-MOTIVE Monthly Audit Newsletter?**
If yes,
    a) **How useful was it?**
    b) **Has it influenced your practice in any way?** If yes, **can you please explain how so?**
    c) **Is there any other information you would like to receive in the newsletter?**
83. **Have you discussed the E-MOTIVE monthly audit newsletter with any colleagues within the hospital? Any with any colleagues outside the hospital?**
84. **Has the newsletter been changed or added to in any way by the staff in this hospital?**
If yes, **can you give me an example?**
85. **Do you receive feedback on PPH practice in this hospital through any other sources?**
If yes, **What are they?**

Section 7 – Wrapping up

86. **Is there anything else that has been introduced in the ward or hospital that might have influenced PPH detection or PPH management during the E-MOTIVE trial?**
    Prompts: any other initiatives, e.g., national guidelines, quality improvement initiatives
87. **Before the E-MOTIVE project was introduced here or since it started, are you aware of any other research studies taking places in your hospital related to maternal health or PPH?**
    If yes**, Do you feel that it has impacted on the E-MOTIVE study at all? How so?**
88. **Has anything else been done in their hospital to try and support the delivery of E-MOTIVE?**
    If yes**, What are they?**
89. **Have you discussed E-MOTIVE with any colleagues from other hospitals at all?**
90. **Is there anything else about the E-MOTIVE project that you would like to share with me that's not been covered in the discussion already?**

These are all of the questions I have for you today, thank you so much for your time.

Appendix Study Instrument 3: Survey (*Control Sites)*

12. DEMOGRAPHICS

For the purpose of this survey we will be focusing on the detection and management of postpartum haemorrhage (PPH) after vaginal births in your hospital. By detection we are referring to the initial diagnosis of PPH. This survey is not focused on the prevention of PPH.

There are no right or wrong answers to any of the questions asked in this survey, we are simply interested in exploring your personal experience and beliefs surrounding PPH. We want to reassure you that the answers you give will not be able to be linked back to you, and will remain anonymous and confidential.

We would like to start with a few questions about your role in your current job, and the facility where you work.

11. What is your current position?

- ☐ Nurse-Midwife/ Midwife
- ☐ Nurse
- ☐ Medical Officer, AMO, Clinical Officer
- ☐ Medical Doctor – Resident
- ☐ Consultant Doctor
- ☐ House Officer in training
- ☐ Non-physician Clinical/Medical Officer
- ☐ Student or Trainee
- ☐ Other (please specify): ____________________

12. How long, in years, have you worked in this position at this hospital?

- ☐ 0-5 years
- ☐ 6-10 years
- ☐ More than 10 years

13. How long, in years, have you worked in this position in total? [Note: The answer you provide must be equal or greater than your answer to the previous question]

- ☐ 0-5 years
- ☐ 6-10 years
- ☐ More than 10 years

13. DETECTING POSTPARTUM HAEMORRHAGE (PPH)

The next section of the survey is about how postpartum haemorrhage (PPH) is detected after a vaginal birth at your hospital.
By detection we are referring to the initial diagnosis of PPH.

14. Is detecting PPH after a vaginal birth part of your clinical role?

- [ ] Yes
- [ ] No

14. DETECTING POSTPARTUM HAEMORRHAGE (PPH)

15. When was the last time you detected and/or assisted in the detection of a PPH?

- [ ] In the last week
- [ ] ≤6 months
- [ ] >6 months
- [ ] I have never performed or assisted in a vaginal birth

16. Which of the following are used to help detect PPH (tick all that apply):

- [ ] Visual estimation
- [ ] Using vital signs (blood pressure, heart rate, respirations)
- [ ] Using uterine tone and size
- [ ] Counting and/or weighing blood-soaked swabs
- [ ] Using a blood loss collection drape

17. In your opinion, are these methods effective in helping you to detect a PPH? (Tick all that apply)

- [ ] Collecting and determining blood loss using a tool (e.g., kidney basin, swabs, linen)
- [ ] Visual estimation of blood loss
- [ ] Using vital signs (blood pressure, heart rate, respiration)
- [ ] Using uterine tone and size
- [ ] Counting and/or weighing blood-soaked swabs
- [ ] Other (please specify): ____________________

18. How often would you use a blood loss collection drape to determine blood loss?

- [ ] Never
- [ ] Rarely
- [ ] Sometimes
- [ ] Often
- [ ] Always

19. If the blood collection drape is used to determine blood loss, when do you apply it?

- [ ] Before birth (e.g., before the baby comes out)
- [ ] After the baby comes out but before placenta is delivered
- [ ] After placenta is delivered
- [ ] Some other time (please specify):

20. If a drape is used, approximately how long is it left in place?

- [ ] 30 minutes (about half an hour)
- [ ] 60 minutes (about an hour)
- [ ] 90 minutes (about an hour and a half)
- [ ] 120 minutes (about 2 hours)
- [ ] Some other time (please specify):

21. Do you like using the drape to determine blood loss?

- [ ] Yes
- [ ] No

22. To what extent do you agree or disagree with the following statements about detecting a PPH after a vaginal birth

	Strongly disagree	Disagree	Neither agree nor disagree	Agree	Strongly agree
My team is disciplined for failing to detect a PPH	☐	☐	☐	☐	☐
My emotional state affects how I detect a PPH	☐	☐	☐	☐	☐
When I detect a PPH during a vaginal birth I receive feedback on my performance	☐	☐	☐	☐	☐
I know what I need to do to detect a PPH	☐	☐	☐	☐	☐
I get sufficient feedback about my performance when I detect a PPH	☐	☐	☐	☐	☐
I want to improve how I detect PPH during a vaginal birth	☐	☐	☐	☐	☐
Fear of repercussions from the patient and	☐	☐	☐	☐	☐

their family affect how I detect a PPH					
If I need help detecting a PPH after a vaginal birth, I can easily get the support or assistance I need from my team	☐	☐	☐	☐	☐
I have the skills to detect PPH	☐	☐	☐	☐	☐
In the past, I have needed assistance when detecting a PPH, as I did not have the competency required	☐	☐	☐	☐	☐
My emotions are negatively affected by detecting a PPH	☐	☐	☐	☐	☐
I intend to improve my knowledge of PPH detection during vaginal birth	☐	☐	☐	☐	☐
I am confident that I can detect a PPH, even when there is little time	☐	☐	☐	☐	☐
Improving PPH detection is not discussed in regular meetings at my facility	☐	☐	☐	☐	☐
If I successfully detect a PPH after a vaginal birth at my health facility I am commended	☐	☐	☐	☐	☐
I feel good if I successfully detect a PPH during a vaginal birth	☐	☐	☐	☐	☐
It is easy to accurately estimate the volume of blood lost after a vaginal birth	☐	☐	☐	☐	☐
It is easy to distinguish between normal blood loss and a PPH after a vaginal birth	☐	☐	☐	☐	☐

15. MANAGING POSTPARTUM HAEMORRHAGE (PPH)

23. Is managing PPH after a vaginal birth part of your clinical role?

- [ ] Yes
- [ ] No
- [ ] Not sure

24. When was the last time you managed and/or assisted in the management of a PPH?

- [ ] In the last week
- [ ] ≤6 months
- [ ] >6 months

25. When managing a PPH, do you typically work as part of a team?

- [ ] Yes, always
- [ ] Yes, most of the time
- [ ] No, I work alone

26. When managing a PPH, what actions would you take for a primary response to a PPH after a vaginal birth? (Tick all that apply)

- [ ] Uterine massage
- [ ] Blood pressure and pulse check
- [ ] Examination for cause of bleeding (placenta, genital tract, bladder)
- [ ] Administer IV fluids
- [ ] Administer tranexamic acid (TXA)
- [ ] Administer uterotonics (e.g., any uterotonics including ergometrine, oxytocin, misoprostol, etc.)
- [ ] Other (please specify):

27. When managing a PPH, do you?

- [ ] Give interventions (e.g., massage, drugs) one at a time without waiting to see the result
- [ ] Give an intervention (e.g., massage, drugs), wait to see if that component works if it doesn't, give another component
- [ ] Give all interventions (e.g., massage, drugs) at once or in quick succession
- [ ] Other (please specify):

28. At your hospital, how readily available are uterotonic drugs (e.g., oxytocin, misoprostol, carbetocin, ergometrine)?

- [ ] Always available
- [ ] Often available
- [ ] Sometimes available
- [ ] Rarely available
- [ ] Never available

29. At your hospital, how readily available is tranexamic acid (TXA)?

- [ ] Always available
- [ ] Often available
- [ ] Sometimes available
- [ ] Rarely available
- [ ] Never available

30. At your hospital, how readily available are IV fluids?

- [ ] Always available
- [ ] Often available
- [ ] Sometimes available
- [ ] Rarely available
- [ ] Never available

31. At your hospital, how readily available is the equipment (e.g., linens/pads, cannulas, gloves) needed to manage a PPH?

- ☐ Always available
- ☐ Often available
- ☐ Sometimes available
- ☐ Rarely available
- ☐ Never available

32. To what extent do you agree or disagree with the following statements:

	Strongly disagree	Disagree	Neither agree nor disagree	Agree	Strongly agree
If I need help managing a PPH after a vaginal birth, I can easily get the support or assistance I need from my team	☐	☐	☐	☐	☐
Fear of repercussions from the patient and their family affect how I manage a PPH	☐	☐	☐	☐	☐
I get sufficient feedback about my performance when I manage a PPH	☐	☐	☐	☐	☐
My team is disciplined for failing to manage a PPH during a vaginal birth	☐	☐	☐	☐	☐
My emotions are negatively affected by managing a PPH	☐	☐	☐	☐	☐
I am confident that I can manage a PPH after a vaginal birth, even when there is little time	☐	☐	☐	☐	☐
I have the skills to manage a PPH after a vaginal birth	☐	☐	☐	☐	☐
I want to improve how I manage PPH during a vaginal birth	☐	☐	☐	☐	☐
If I successfully manage a PPH at my health facility I am commended	☐	☐	☐	☐	☐
I feel good if I successfully manage a PPH during a vaginal birth	☐	☐	☐	☐	☐
Improving PPH management is not discussed in regular meetings at my facility	☐	☐	☐	☐	☐

I intend to improve my knowledge of PPH management	☐	☐	☐	☐	☐
My emotional state affects how I manage a PPH	☐	☐	☐	☐	☐
In the past, I have needed assistance when managing a PPH after a vaginal birth, as I did not have the competency required	☐	☐	☐	☐	☐
I know what I need to do to manage a PPH after a vaginal birth	☐	☐	☐	☐	☐
When I manage a PPH I receive feedback on my performance	☐	☐	☐	☐	☐

16. TRAINING

Next, we would like to focus on the training you have received around detecting and managing a PPH

33. Have you received any PPH training in the last 2 years?

- [ ] Yes
- [ ] No

17. TRAINING

34. When was the PPH training?

- [ ] In the last 3 months
- [ ] In the last 6 months
- [ ] Over 6 months ago
- [ ] Over a year ago

35. Do you think that you and your colleagues need additional training on PPH detection and management?

- [ ] Yes
- [ ] No
- [ ] Not sure

36. Do you use any of the following guidelines or protocols for detecting and managing PPH? (Tick all that apply)

- [ ] International guidelines and/or protocols (WHO)
- [ ] National guidelines and/or protocols (Ministry of Health)
- [ ] Local guidelines and/or protocols (District/Regional)
- [ ] Hospital guidelines and/or protocols

37. Who delivered the most recent training on PPH? (Tick all that apply)

- [ ] Trainers from your hospital
- [ ] Trainers from the district or region
- [ ] Trainer from non-governmental organisations (NGO)
- [ ] Trainers from the Ministry of Health
- [ ] Medical/nursing/midwifery school
- [ ] Other (please specify):

38. What was the format of the training? (Tick all that apply)

- [ ] Presentation
- [ ] Lectures
- [ ] Textbook/Pamphlets
- [ ] Simulation
- [ ] Demonstrations
- [ ] Workshops
- [ ] Other (please specify):

18. FINAL QUESTIONS

39. Have any of the following been introduced in the ward or hospital during the last 6 months?

	Yes	No	Don't know
Quality improvement initiatives on maternal health and/or PPH	☐	☐	☐

If yes, please give details here

40. Have any of the following been introduced in the ward or hospital during the last 6 months?

	Yes	No	Don't know
Provision of national or local guidelines/protocols	☐	☐	☐

If yes, please give details here

41. Have any other maternal health or PPH research studies taking place at the moment or in the last 6 months in your hospital?

- ☐ No
- ☐ Don't know
- ☐ Yes (please give details here)

42. Have any other maternal health or PPH research studies taking place at the moment or in the last 6 months in your hospital?

- ☐ Yes
- ☐ No
- ☐ Not sure

Appendix Study Instrument 4: Survey (*Intervention Sites*)

11. Demographics with information

For the purposes of this survey, we are focusing on your views and experiences of using the E-MOTIVE bundle to care for a woman with a postpartum haemorrhage (PPH).

Please give honest feedback, there are no right or wrong answers to any of the questions asked in this survey, we are simply interested in exploring your personal experience and beliefs about detecting and managing PPH using E-MOTIVE bundle.

We want to reassure you that the answers you give will not be able to be linked back to you and will remain anonymous and confidential.

We would like to start with a few questions about your role in your current job, and the facility where you work. Please answer work through the questions on this page before progressing on to the next section of questions.

1. What is your current position?

- [ ] Nurse-Midwife/ Midwife
- [ ] Nurse
- [ ] Medical Officer, AMO, Clinical Officer
- [ ] Medical Doctor – Resident
- [ ] Consultant Doctor
- [ ] House Officer in training
- [ ] Non-physician Clinical/ Medical Officer
- [ ] Student or Trainee
- [ ] Other (please specify):

2. How long, in years, have you worked in this position at this hospital?

- [ ] 0-5 Years
- [ ] 6-10 years
- [ ] More than 10 years

1. 3. How long, in years, have you worked in this position in total?

Note: The answer you provide must be equal or greater than your answer to the previous question

- [ ] 0-5 years
- [ ] 6-10 years
- [ ] More than 10 years

12. Detecting postpartum haemorrhage

The next section of the survey is about how postpartum haemorrhage is detected after a vaginal birth at your hospital. By detection we are referring to the initial diagnosis of PPH

2. 4. Is detecting PPH after a vaginal birth part of your clinical role?

- ☐ Yes
- ☐ No

5. When was the last time you detected and/or assisted in the detection of a PPH?

- ☐ In the last week
- ☐ ≤6 months
- ☐ >6 months
- ☐ I have never performed or assisted with a vaginal delivery

13. Detecting postpartum haemorrhage

6. Do you use a drape for blood loss collection after vaginal birth? (***The calibrated drapes are clear plastic marked with small black lines/markers at intervals between 0-1600ml, in addition the marker at 300ml is yellow and the marker at 500ml is red.***)

- ☐ Yes, a calibrated drape
- ☐ Yes, a non-calibrated drape (no lines)
- ☐ No

14. Detecting postpartum haemorrhage

3. 7. How often do you use a calibrated drape used to detect a PPH?

- [ ] Never
- [ ] Rarely
- [ ] Sometimes
- [ ] Often
- [ ] Always

15. Detecting postpartum haemorrhage

4. **8. Why would you not use a calibrated drape to detect a PPH? (Please, tick all that apply)**

- ☐ Inconsistent supply
- ☐ Not available on the ward
- ☐ Not easily located when needed
- ☐ Prefer not to use it

5. 9. If you use a calibrated drape, when do you apply it?

- ☐ Before birth (e.g., before the baby comes out)
- ☐ After the baby comes out but before placenta is delivered
- ☐ After placenta is delivered
- ☐ Some other time (please specify)

Please, specify "some other time" :

6. 10. If a calibrated drape is used, approximately how long is it left in place?

- ☐ 30 minutes (about half an hour)
- ☐ 60 minutes (about an hour)
- ☐ 90 minutes (about an hour and a half)
- ☐ 120 minutes (about 2 hours)
- ☐ Some other time (please specify)

Please, specify "some other time" :

18. Detecting postpartum haemorrhage

7. 11. Do you use any of the following to help detect PPH (in addition, to the drape, if used)? (Tick all that apply)

- ☐ Visual estimation
- ☐ Using vital signs (blood pressure, heart rate, respirations)
- ☐ Using uterine tone and size
- ☐ Counting and/or weighing blood-soaked swabs
- ☐ Blood loss monitoring chart
- ☐ Other (please specify):

8. 12. In your opinion, how effective are each of these methods in helping you to detect a PPH? (Tick all that apply)

	Not effective	Somewhat effective	Very effective
Collecting and measuring blood loss using a calibrated drape	☐	☐	☐
Visual estimation of blood loss	☐	☐	☐
Using vital signs (blood pressure, pulse, heart rate, respirations)	☐	☐	☐
Using uterine tone and size	☐	☐	☐
Counting and/or weighing blood-soaked swabs	☐	☐	☐
Blood loss monitoring chart	☐	☐	☐

19. Detecting postpartum haemorrhage

9. **13. To what extent do you agree or disagree with the following statements**

	Strongly disagree	Disagree	Neither agree nor disagree	Agree	Strongly agree
I know what I need to do to detect a PPH during a vaginal birth using the calibrated drape	☐	☐	☐	☐	☐
I have the skills needed to use the calibrated drape to measure blood loss	☐	☐	☐	☐	☐
It is easier to measure blood loss using a calibrated drape than it was before the calibrated drape was introduced	☐	☐	☐	☐	☐
I have become more confident at detecting a PPH since the calibrated drape was introduced	☐	☐	☐	☐	☐
The detection of PPH has improved at this hospital since the calibrated drape was introduced	☐	☐	☐	☐	☐
I feel less stress when using the calibrated drape to detect a PPH than it was before the calibrated drape was introduced	☐	☐	☐	☐	☐
I think my workload has increased since the calibrated drape was introduced	☐	☐	☐	☐	☐
I like using the calibrated drape to detect a PPH	☐	☐	☐	☐	☐
It is clear to me how using the calibrated drape to detect a PPH during a vaginal birth will improve PPH detection	☐	☐	☐	☐	☐
Team-working has improved since the calibrated drape was introduced to detect a PPH	☐	☐	☐	☐	☐
My team is disciplined for failing to use the calibrated drape to detect a PPH	☐	☐	☐	☐	☐

I do not use the calibrated drape because my colleagues do not use it	☐	☐	☐	☐	☐
I do not use the calibrated drape because women do not want to lie on it	☐	☐	☐	☐	☐
Our team has received sufficient feedback about how we are using the calibrated drape to detect PPH	☐	☐	☐	☐	☐
I plan to regularly use the calibrated drape to detect a PPH	☐	☐	☐	☐	☐
The drape will help us to recognise a PPH more rapidly/ accurately	☐	☐	☐	☐	☐
I think that PPH is detected earlier when a calibrated drape is used	☐	☐	☐	☐	☐
I feel that use of the calibrated drape has interfered with my other clinical responsibilities	☐	☐	☐	☐	☐

20. Managing Postpartum Haemorrhage

14. Is managing PPH after a vaginal birth part of your clinical role?

- [ ] Yes
- [ ] No

21. Managing postpartum haemorrhage

10. 15. When was the last time you managed and/or assisted in the management of a PPH?

- ☐ last week
- ☐ ≤6 months
- ☐ >6 months

11. 16. When managing a PPH, do you typically work as part of a team?

- ☐ Yes, always
- ☐ Yes, most of the time
- ☐ No, I work alone

12. 17. When managing a PPH, what actions would you take for a primary response to a PPH after a vaginal birth? (Tick all that apply)

- ☐ Uterine massage
- ☐ Blood pressure and pulse check
- ☐ Examination for cause of bleeding (placenta, genital tract, bladder)
- ☐ Administer IV fluids
- ☐ Administer tranexamic acid (TXA)
- ☐ Administer uterotonics (e.g., any uterotonics including ergometrine, oxytocin, misoprostol, etc.)
- ☐ Other (please specify): ____________________

22. Managing postpartum haemorrhage

MOTIVE bundle stands for uterine **M**assage, **O**xytocin, **T**XA, **IV** fluids and **E**xamination & Escalation (if required)

13. 18. When managing a PPH, how often do you use all components of the MOTIVE bundle?

- [ ] Never
- [ ] Rarely
- [ ] Sometimes
- [ ] Usually
- [ ] Always

14. **19. When using the MOTIVE bundle, do you?**

- [ ] Give all the components at once or in quick succession
- [ ] Give components one at a time without waiting to see the result
- [ ] Give a component, wait to see if that component works if it doesn’t, give another component
- [ ] Other (please specify):

24. Managing postpartum haemorrhage

15. 20. To what extent do you agree or disagree with the following statements about managing a PPH after a vaginal birth.

	Strongly disagree	Disagree	Neither agree nor disagree	Agree	Strongly agree
I know what I need to do when using the MOTIVE bundle to manage a PPH	☐	☐	☐	☐	☐
I have the skills to use the MOTIVE bundle to manage a PPH	☐	☐	☐	☐	☐
If I need help managing a PPH, I can easily get the support or assistance from my colleagues or managers.	☐	☐	☐	☐	☐
I am confident that I can manage a PPH, even when there is little time.	☐	☐	☐	☐	☐
I feel less stress when using the MOTIVE bundle to manage a PPH, compared to before MOTIVE bundle was introduced.	☐	☐	☐	☐	☐
I think my workload has decreased since the MOTIVE bundle was introduced.	☐	☐	☐	☐	☐
Team-working is better since the MOTIVE bundle was introduced.	☐	☐	☐	☐	☐
My team is disciplined for failing to use the MOTIVE bundle.	☐	☐	☐	☐	☐
If my colleagues do not use MOTIVE bundle, then I may not use it either.	☐	☐	☐	☐	☐
PPH management is more effective when the MOTIVE bundle is used.	☐	☐	☐	☐	☐
I regularly use the MOTIVE bundle to manage a PPH.	☐	☐	☐	☐	☐
I like using the MOTIVE bundle to manage a PPH.	☐	☐	☐	☐	☐
It is clear to me how	☐	☐	☐	☐	☐

using the MOTIVE bundle has improved PPH management.	☐	☐	☐	☐	☐
I feel using the MOTIVE bundle to manage a PPH has interfered with other competing priorities.	☐	☐	☐	☐	☐

25. Managing postpartum haemorrhage

16. 21. To manage PPH ___

	Always available	Often available	Sometimes available	Rarely available	Never available
At your hospital, how readily available are uterotonic drugs (e.g., oxytocin, misoprostol, carbetocin, ergometrine)?	☐	☐	☐	☐	☐
At your hospital, how readily available is tranexamic acid (TXA)?	☐	☐	☐	☐	☐
At your hospital, how readily available are IV fluids?	☐	☐	☐	☐	☐

17. 22. Do you have a PPH trolley/carry case/box in the labor, delivery, or postnatal wards?

☐ Yes in all (the labor, delivery, or postnatal wards)

☐ Yes - in some of these wards

☐ No - none of these wards

26. Managing postpartum haemorrhage

18. 23. How often do you typically use the PPH trolley/carry case/ box to manage a PPH?

- [ ] Never
- [ ] Rarely
- [ ] Sometimes
- [ ] Often
- [ ] Always

19. **24. Why would you <u>not</u> use the PPH trolley/carry case/box? <u>(please, tick all that apply)</u>**

- ☐ Not adequately stocked
- ☐ Inconsistent supplies
- ☐ Incorrect supplies
- ☐ Not easily located when required
- ☐ Storage issues - lack of space
- ☐ PPH items kept in another nearby place
- ☐ Drugs are kept in the fridge
- ☐ Forget to use it
- ☐ Not allowed to use it - because of role
- ☐ Can't use - damaged and/or broken
- ☐ Other (please specify):

28. Managing postpartum haemorrhage

20. 25. To what extent do you agree or disagree with the following statements about managing a PPH after a vaginal birth

	Strongly disagree	Disagree	Neither agree nor disagree	Agree	Strongly agree
I know what PPH items are stored in the trolley/ carry-case/ box	☐	☐	☐	☐	☐
It is easier to find PPH items when stored in the trolley/ carry-case/ box	☐	☐	☐	☐	☐
I think having the trolley/ carry-case/ box improves how quickly you can respond to a PPH	☐	☐	☐	☐	☐
I do not use the trolley/ carry-case/ box because my colleagues do not use it	☐	☐	☐	☐	☐
I do not want to use the trolley/ carry-case/ box because there are no benefits	☐	☐	☐	☐	☐
Anyone working on the ward can fetch items from the trolley/ carry-case/ box when a PPH is detected	☐	☐	☐	☐	☐
I experience less stress when PPH items are stored in the trolley/ carry-case/ box	☐	☐	☐	☐	☐
My team receives feedback about the stocking levels of the trolley/ carry-case/ box	☐	☐	☐	☐	☐
My team like having a trolley/ carry-case/ box	☐	☐	☐	☐	☐
It is clear to me how having a PPH trolley/ carry-case/ box has improved of PPH management	☐	☐	☐	☐	☐

21. 26. Have you received any feedback or information about your team's use of the E-MOTIVE bundle?

- [ ] Yes
- [ ] No

22. From who did you receive feedback about your team's use of the E-MOTIVE bundle?

- [ ] Hospital management
- [ ] Team manager
- [ ] Other (please specify):

23. 27. Who receives feedback or information about your teams use of the E-MOTIVE bundle?

- [ ] All staff in the maternity unit
- [ ] Only managerial and/or senior staff
- [ ] Only doctors
- [ ] Only midwives or nurses
- [ ] I do not know

24. 28. In what format is feedback or information about your teams use of E-MOTIVE shared? (Tick all that apply)

- [ ] E-MOTIVE Monthly Audit newsletter
- [ ] E-MOTIVE posters displayed in the ward
- [ ] Discussions at team meetings
- [ ] Individual feedback
- [ ] I do not know

25. 29. How often is feedback or information about your teams' use of the E-MOTIVE bundle shared?

- [ ] Every 2-3 weeks
- [ ] Every month
- [ ] Only once or twice in the last 6 months
- [ ] I do not know

26. 30. To what extent do you agree or disagree with the following statements about feedback.

	Strongly disagree	Disagree	Neither agree nor disagree	Agree	Strongly agree
I am aware of the feedback received by the team	☐	☐	☐	☐	☐
I get sufficient feedback about using the E-MOTIVE bundle	☐	☐	☐	☐	☐
Receiving feedback has helped me identify where improvements are needed	☐	☐	☐	☐	☐
I have used the E-MOTIVE bundle more because of the feedback, I have received	☐	☐	☐	☐	☐
Team-working is better since feedback was introduced	☐	☐	☐	☐	☐
I worry about receiving negative feedback because of what it might say	☐	☐	☐	☐	☐
I like receiving feedback about PPH management at this hospital	☐	☐	☐	☐	☐
I understand how receiving PPH management feedback has improved my performance	☐	☐	☐	☐	☐

32. E-MOTIVE Training

Next, we would like to focus on the training you have received around detecting and managing a PPH using the E-MOTIVE bundle.

27. 31. Were you trained on using the E-MOTIVE bundle to detect and manage a PPH?

- [ ] Yes
- [ ] No

33. E-MOTIVE Training

28. 32. Why have you not received training on the E-MOTIVE bundle?

- [ ] E-MOTIVE training has not happened in my hospital
- [ ] E-MOTIVE training happened, but I was unable to attend
- [ ] E-MOTIVE training happened before I started working at this hospital
- [ ] E-MOTIVE training happened, but I decided not to attend because there is not enough time
- [ ] E-MOTIVE training happened, but I decided not to attend because it is not a priority
- [ ] E-MOTIVE training happened, but I decided not to attend because it is not relevant to my role
- [ ] Other (please specify):

34. E-MOTIVE Training

29. 33. Is facilitating E-MOTIVE training as an onsite trainer part of your role?

- [ ] Yes
- [ ] No

30. 34. Did you receive training from:

	Yes	No
Jhpiego trainers	☐	☐
E-MOTIVE trainers	☐	☐
E-MOTIVE Champion	☐	☐
Other staff member	☐	☐
I have not received training	☐	☐

Other (please, specify)

35. E-MOTIVE Training

31. **35. What was the format of the E-MOTIVE training? (<u>Please tick all that apply</u>)**

- [ ] Presentation
- [ ] Lectures
- [ ] Textbook/Pamphlets
- [ ] Simulation
- [ ] Demonstration
- [ ] Workshops
- [ ] Other (please specify):

____

36. E-MOTIVE Training

32. 36. Have you attended any practice drill sessions?

- [ ] Yes
- [ ] No

37. E-MOTIVE Training

33. 37. How many practice drill sessions have you participated in the last 6 months?

- [ ] 1-2
- [ ] 3-4
- [ ] All 5
- [ ] None

38. E-MOTIVE Training

34. 38. Why have you not attended any practice drill sessions?

- [ ] No practice drill sessions held
- [ ] No practice drill sessions held for my shift
- [ ] I was absent
- [ ] I choose not to attend
- [ ] Not enough time
- [ ] Not a priority
- [ ] Not helpful
- [ ] Not part of my role
- [ ] Other (please specify):

39. E-MOTIVE Training

35. 39. To what extent do you agree with the following statements about training on detection and monitoring PPH:

	Strongly disagree	Disagree	Neither agree nor disagree	Agree	Strongly agree
I have a better understanding of using the E-MOTIVE bundle to detect and manage a PPH	☐	☐	☐	☐	☐
I have been adequately trained in using the MOTIVE bundle to manage a PPH	☐	☐	☐	☐	☐
I think the training on managing a PPH using the MOTIVE bundle was helpful	☐	☐	☐	☐	☐
I think the training identified areas where improvements in PPH management could be made	☐	☐	☐	☐	☐
I have been adequately trained in using the calibrated drape to detect a PPH	☐	☐	☐	☐	☐
I think the training on using the calibrated drape was helpful	☐	☐	☐	☐	☐
I think the training identified areas where improvements in PPH detection could be made	☐	☐	☐	☐	☐

36. 40. I have recently received other PPH education and training other than the E-MOTIVE training

☐ Yes

☐ No

If "Yes", please, specify

40. E-MOTIVE CHAMPIONS

37. 41. Is being an E-MOTIVE champion part of your role

- [ ] Yes
- [ ] No

38. 42. Are you aware of the E-MOTIVE champions at your hospital?

- [ ] Yes
- [ ] No

41. E-MOTIVE CHAMPIONS

39. 43. To what extent do you agree with these statements about E-Motive Champions:

	Strongly disagree	Disagree	Neither agree nor disagree	Agree	Strongly agree
Having champions support staff to use E-MOTIVE is helpful	☐	☐	☐	☐	☐
The champions are readily available to the staff	☐	☐	☐	☐	☐
Having champions has increased the use of the E-MOTIVE bundle	☐	☐	☐	☐	☐
Having champions improved how the E-MOTIVE bundle is used	☐	☐	☐	☐	☐
The support of champions has reduced any concerns about using the E-MOTIVE bundle	☐	☐	☐	☐	☐
I like champions giving support and advice on the E-MOTIVE bundle to the team	☐	☐	☐	☐	☐
It is clear to me how having champions has improved the use of the E-MOTIVE bundle	☐	☐	☐	☐	☐

42. FINAL QUESTIONS

40. 44. Have any of the following been introduced in the ward or hospital during the E-MOTIVE trial?

	Yes (please specify in the Comment box below)	No	Don’t know
Other Quality Improvement (QI) initiatives on maternal health and/or PPH	☐	☐	☐
Provision of national or local guidelines/protocols	☐	☐	☐

If "Yes", please, specify

41. 45. Have any other maternal health or PPH research studies taking place at the moment or between 2019 and 2022 in your hospital?

- ☐ Yes (please specify in the Comment box below)
- ☐ No
- ☐ Don't know

If "Yes", please, specify

42. 46. Have you discussed the E-MOTIVE study with any colleagues from other hospitals at all?

- [ ] Yes (please specify in the Comment box below)
- [ ] No
- [ ] Not sure

If "Yes", please, specify

Appendix Study Instrument 5: Observation guide (*Intervention Sites*)

HCP observations

Record ID ______________________

REDCap Username ______________________

1. Details of observations

Date of observations ______________________

Hospital name
- ◯ [redacted]
- ◯ [redacted]
- ◯ [redacted]
- ◯ [redacted]
- ◯ [redacted]
- ◯ [redacted]
- ◯ [redacted]

E-MOTIVE patient ID
One E-MOTIVE ID per HCP observation ______________________

Name of observer(s) ______________________

2. Maternal risk factors

Maternal weight (at booking) ______________________ (kg)

Maternal height (at booking) ______________________ (cm)

Does the woman suffer from any of the following conditions?

	Yes	No
Hypertension	◯	◯
Diabetes	◯	◯
Autoimmune disease	◯	◯
Coagulation disorder	◯	◯
Sexually transmitted infection	◯	◯
Heart disease	◯	◯
Kidney disease	◯	◯
HIV	◯	◯

Malaria	◯	◯
Hepatitis B	◯	◯
Hepatitis C	◯	◯
Chagas disease	◯	◯
Tetanus	◯	◯
Tuberculosis	◯	◯
Epilepsy	◯	◯
Uterine fibroids	◯	◯
Other	◯	◯

If other, please specify: ______________________

3. Pregnancy, labour and birth related risk factors

Was a Hb measurement taken during pregnancy? ◯ Yes ◯ No

If yes, what was the result of the last Hb measurement?
Please enter the most recent Hb measurement ______________________ (g/dl)

If yes, what date was the last Hb measurement taken? (DD/MM/YYYY)
Please enter the date of the most recent Hb measurement ______________________

Did the woman have any of the following pregnancy, labour or birth related risk factors?

	Yes	No
Previous neonatal death	◯	◯
Take regular iron tablets for >1 month in pregnancy	◯	◯
Hookworm treated in pregnancy	◯	◯
Placenta previa or low lying, accreta, increta or percreta	◯	◯
Antepartum haemorrhage (APH)	◯	◯
Intrauterine growth restriction (IUGR)	◯	◯
Polyhydramnios	◯	◯
Oligohydramnios	◯	◯
Placental abruption	◯	◯
Gestational diabetes	◯	◯
Chorioamnionitis (foul-smelling vaginal discharge)	◯	◯

Pregnancy induced hypertension (PIH) or gestational hypertension	◯	◯
Pre-eclampsia	◯	◯
Eclampsia	◯	◯
Intrapartum haemorrhage	◯	◯
Febrile in labour (temperature >38°C)	◯	◯
Pushing > 60 minutes	◯	◯
Induction of labour	◯	◯
Augmentation of labour	◯	◯
Received antibiotics in labour	◯	◯
Meconium liquor	◯	◯
Compound presentation (vertex and another presenting part simultaneously e.g., hand and vertex)	◯	◯
Breech presentation	◯	◯
Malpresentation or malposition (presenting part other than vertex or breech, e.g. face presentation)	◯	◯
Shoulder dystocia	◯	◯
Uterine inversion	◯	◯
Uterine rupture	◯	◯
Other	◯	◯

If other, please specify: ______________________________

Did the woman arrive to the hospital in poor condition prior to giving birth?
◯ Yes
◯ No

If yes, please describe in detail her condition and the diagnosis: ______________________________

4. Exploring issues with drape use

What time was the baby born? (HH:MM, 24 hr clock e.g. 22:31) ______________________________
((HH:MM, 24 hr clock e.g. 22:31))

Who conducted the birth?

- ◯ Midwife
- ◯ Research midwife
- ◯ Student Midwife
- ◯ Doctor
- ◯ Intern
- ◯ Other

If other, please state which cadre: ______________________

Who else was present at the birth (other than the practitioner conducting the birth)?

	Yes	No
Midwife	◯	◯
Research midwife	◯	◯
Student Midwife	◯	◯
Doctor	◯	◯
Intern	◯	◯
Other	◯	◯

Number of midwives present: ______________________

Number of research midwives present: ______________________

Number of student midwives present: ______________________

Number of doctors present: ______________________

Number of interns present: ______________________

If other, please state which cadre: ______________________

Number of other persons present: ______________________

Was oxytocin given for Active Management of Third Stage of Labour (AMTSL)?

- ◯ Yes
- ◯ No

Who administered oxytocin?

- ◯ Midwife
- ◯ Student Midwife
- ◯ Research midwife
- ◯ Doctor
- ◯ Intern
- ◯ Other

If other, please state: ______________________

What brand of oxytocin was administered?	◯ Climax Oxytocin<br>◯ Curtocin Injection<br>◯ DERM<br>◯ Evatocin<br>◯ Extocin<br>◯ Galaxy<br>◯ Labtocin<br>◯ Mark<br>◯ Oxymed<br>◯ Oxynir<br>◯ Oxyt<br>◯ Oxytocin 10<br>◯ Oxyzed 10IU<br>◯ Pitocin<br>◯ Pitons Oxytocin Injection<br>◯ Spec Oxytocin<br>◯ Syntocinon<br>◯ Utocin<br>◯ Vitocin-10<br>◯ Zytocin<br>◯ Not known<br>◯ Other
If other, please state:	______________________
What time was oxytocin administered?<br><br>(HH:MM, 24 hr clock e.g. 22:31)	______________________
What total dose of oxytocin was administered in IU?	______________________<br>(IU)
What route was used for oxytocin administration?<br><br>Select all that apply	☐ IM<br>☐ IV
Was misoprostol given for Active Management of Third Stage of Labour (AMTSL)?	◯ Yes<br>◯ No
Who administered misoprostol?	◯ Midwife<br>◯ Student Midwife<br>◯ Research midwife<br>◯ Doctor<br>◯ Intern<br>◯ Other
If other, please state:	______________________
What time was misoprostol administered?<br><br>(HH:MM, 24 hr clock e.g. 22:31)	______________________
What dose of misoprostol was administered?	◯ 400mcg<br>◯ 600mcg<br>◯ 800mcg<br>◯ Other

If other, please state:	______________________ (mcg)
What route was used for misoprostol administration?<br><br>Select all that apply	☐ Oral or sublingual<br>☐ Per rectal (PR)
Was ergometrine given for Active Management of Third Stage of Labour (AMTSL)?	◯ Yes<br>◯ No
Who administered ergometrine?	◯ Midwife<br>◯ Student Midwife<br>◯ Research midwife<br>◯ Doctor<br>◯ Intern<br>◯ Other
If other, please state:	______________________
What time was ergometrine administered?<br><br>(HH:MM, 24 hr clock e.g. 22:31)	______________________
What total dose of ergometrine was administered in mcg?	______________________ (mcg)
What route was used for ergometrine administration?<br><br>Select all that apply	☐ IM<br>☐ IV
Was carbetocin given for Active Management of Third Stage of Labour (AMTSL)?	◯ Yes<br>◯ No
Who administered carbetocin?	◯ Midwife<br>◯ Student Midwife<br>◯ Research midwife<br>◯ Doctor<br>◯ Intern<br>◯ Other
If other, please state:	______________________
What time was carbetocin administered?<br><br>(HH:MM, 24 hr clock e.g. 22:31)	______________________
What total dose of carbetocin was administered in mcg?	______________________ (mcg)
What route was used for carbetocin administration?<br><br>Select all that apply	☐ IM<br>☐ IV

Please provide details of any other medication that was administered	__________________
Who applied the drape?	◯ Midwife<br>◯ Student Midwife<br>◯ Research midwife<br>◯ Doctor<br>◯ Intern<br>◯ Other
If other, please state:	__________________
Is the drape tied and secured around the woman's waist?	◯ Yes<br>◯ No
What time was the funnel portion of the drape opened ready to collect blood postpartum?<br>(HH:MM, 24 hr clock e.g. 22:31)	__________________<br>((HH:MM, 24 hr clock e.g. 22:31))

THE TIME YOU HAVE ENTERED IS BEFORE THE TIME THE BABY WAS BORN. PLEASE CHECK YOUR ENTRY AND EDIT ACCORDINGLY.

Is only blood and blood-soaked gauze/pads going into the funnel?	◯ Yes<br>◯ No
If no, what else is put into the funnel?<br><br>Select all that apply	☐ Cotton wool<br>☐ Placenta<br>☐ Umbilical cord<br>☐ Other
If other, please state:	__________________
What time was the placenta delivered?<br>(HH:MM, 24 hr clock e.g. 22:31)	__________________<br>((HH:MM, 24 hr clock e.g. 22:31))

THE TIME YOU HAVE ENTERED IS BEFORE THE TIME THE BABY WAS BORN. PLEASE CHECK YOUR ENTRY AND EDIT ACCORDINGLY.

Was Manual Removal of Placenta (MROP) performed?	◯ Yes<br>◯ No
Was the placenta checked before discarding?	◯ Yes<br>◯ No
If yes, what was the status of the placenta and/or membranes?	◯ Complete<br>◯ Incomplete<br>◯ Unknown

3. Vital signs, observations and blood loss measurement

If any observations and/or vital signs were completed, please select 'Yes' and answer the questions

If no observations and/or vital signs were completed, select no and go to Section 4. Outcomes on page 15

1st set of observations

Were the first set of observations and/or vital signs conducted?	◯ Yes<br>◯ No
Time observations and/or vital signs performed (HH:MM, 24 hr clock e.g. 22:31)	______________________<br>((HH:MM, 24 hr clock e.g. 22:31))
THE TIME YOU HAVE ENTERED IS BEFORE THE TIME THE DRAPE WAS APPLIED. PLEASE CHECK YOUR ENTRY AND EDIT ACCORDINGLY.	
Are the observations and/or vital signs being documented in the woman's clinical record?	◯ Yes<br>◯ No
If yes, where are the observations and/or vital signs documented?<br><br>Select all that apply	☐ Blood loss monitoring and WHO first response PPH bundle triggering chart<br>☐ Partograph<br>☐ Written in patient notes<br>☐ Early warning chart<br>☐ Treatment sheet<br>☐ Nursing Cardex<br>☐ Other
If other, please state:	______________________
Time observations documented (HH:MM, 24 hr clock e.g. 22:31)	______________________<br>((HH:MM, 24 hr clock e.g. 22:31))
THE TIME YOU HAVE ENTERED IS BEFORE THE TIME THE DRAPE WAS APPLIED. PLEASE CHECK YOUR ENTRY AND EDIT ACCORDINGLY.	
Who completed the observations and/or vital signs?	◯ Midwife<br>◯ Research midwife<br>◯ Student Midwife<br>◯ Doctor<br>◯ Intern<br>◯ Other
If other, please state:	______________________
Did you observe a blood pressure reading being taken?	◯ Yes<br>◯ No
If yes, provide the documented blood pressure reading (mmHg)	______________________<br>(e.g. 120/180 (DO NOT ADD UNITS))
or tick if not documented	◯ Not documented

Did you observe a pulse reading being taken?	◯ Yes<br>◯ No
If yes, provide the documented pulse reading (beats per minute)	__________________<br>(e.g. 82)
or tick if not documented	◯ Not documented
Did you observe uterine tone being assessed?	◯ Yes<br>◯ No
If yes, what was the documented uterine tone status?	◯ Soft<br>◯ Hard<br>◯ Not documented
Did you observe the practitioner assessing vaginal blood loss?	◯ Yes<br>◯ No
If yes, what was the documented vaginal blood loss status?<br><br>Select all that apply	☐ Heavy flow<br>☐ Large clots<br>☐ Trickle<br>☐ Normal<br>☐ Not documented
Is the blood swept into the funnel under the woman?	◯ Yes<br>◯ No
Did you observe the practitioner assessing the calibrated drape measurement line?	◯ Yes<br>◯ No
If yes, how is the funnel of the drape lying when reading the calibration lines?	◯ Hanging over the edge of the bed<br>◯ Flat on the bed
If flat on the bed, how are the calibration lines on the drape being read?	◯ Moved to the edge of the bed<br>◯ Visualised flat on the bed<br>◯ Other
If other, please state:	__________________
Was the cumulative calibrated drape measurement line reading documented?	◯ Yes<br>◯ No
If yes, what was the documented cumulative calibrated drape measurement line reading?	__________________<br>(ml)
2nd set of observations	
Were the second set of observations and/or vital signs conducted?	◯ Yes<br>◯ No
Time observations and/or vital signs performed (HH:MM, 24 hr clock e.g. 22:31)	__________________<br>((HH:MM, 24 hr clock e.g. 22:31))

THE TIME YOU HAVE ENTERED IS BEFORE THE TIME THE DRAPE WAS APPLIED. PLEASE CHECK YOUR ENTRY AND EDIT ACCORDINGLY.

Are the observations and/or vital signs being documented in the woman's clinical record?
- ○ Yes
- ○ No

If yes, where are the observations and/or vital signs documented?

Select all that apply
- ☐ Blood loss monitoring and WHO first response PPH bundle triggering chart
- ☐ Partograph
- ☐ Written in patient notes
- ☐ Early warning chart
- ☐ Treatment sheet
- ☐ Nursing Cardex
- ☐ Other

If other, please state: ______________________

Time observations documented (HH:MM, 24 hr clock e.g. 22:31)
____
((HH:MM, 24 hr clock e.g. 22:31))

THE TIME YOU HAVE ENTERED IS BEFORE THE TIME THE DRAPE WAS APPLIED. PLEASE CHECK YOUR ENTRY AND EDIT ACCORDINGLY.

Who completed the observations and/or vital signs?
- ○ Midwife
- ○ Research midwife
- ○ Student Midwife
- ○ Doctor
- ○ Intern
- ○ Other

If other, please state: ______________________

Did you observe a blood pressure reading being taken?
- ○ Yes
- ○ No

If yes, provide the documented blood pressure reading (mmHg)
____
(e.g. 120/180)

or tick if not documented
- ○ Not documented

Did you observe a pulse reading being taken?
- ○ Yes
- ○ No

If yes, provide the documented pulse reading (beats per minute)
____
(e.g. 82)

or tick if not documented
- ○ Not documented

Did you observe uterine tone being assessed?
- ○ Yes
- ○ No

If yes, what was the documented uterine tone status?	◯ Soft<br>◯ Hard<br>◯ Not documented
Did you observe the practitioner assessing vaginal blood loss?	◯ Yes<br>◯ No
If yes, what was the documented vaginal blood loss status?<br><br>Select all that apply	☐ Heavy flow<br>☐ Large clots<br>☐ Trickle<br>☐ Normal<br>☐ Not documented
Is the blood swept into the funnel under the woman?	◯ Yes<br>◯ No
Did you observe the practitioner assessing the calibrated drape measurement line?	◯ Yes<br>◯ No
If yes, how is the funnel of the drape lying when reading the calibration lines?	◯ Hanging over the edge of the bed<br>◯ Flat on the bed
If flat on the bed, how are the calibration lines on the drape being read?	◯ Moved to the edge of the bed<br>◯ Visualised flat on the bed<br>◯ Other
If other, please state:	__________________________
Was the cumulative calibrated drape measurement line reading documented?	◯ Yes<br>◯ No
If yes, what was the documented cumulative calibrated drape measurement line reading?	__________________________<br>(ml)
3rd set of observations	
Were the third set of observations and/or vital signs conducted?	◯ Yes<br>◯ No
Time observations and/or vital signs performed (HH:MM, 24 hr clock e.g. 22:31)	__________________________<br>((HH:MM, 24 hr clock e.g. 22:31))
THE TIME YOU HAVE ENTERED IS BEFORE THE TIME THE DRAPE WAS APPLIED. PLEASE CHECK YOUR ENTRY AND EDIT ACCORDINGLY.	
Are the observations and/or vital signs being documented in the woman's clinical record?	◯ Yes<br>◯ No

If yes, where are the observations and/or vital signs documented?<br><br>Select all that apply	☐ Blood loss monitoring and WHO first response PPH bundle triggering chart<br>☐ Partograph<br>☐ Written in patient notes<br>☐ Early warning chart<br>☐ Treatment sheet<br>☐ Nursing Cardex<br>☐ Other
If other, please state:	__________________________________
Time observations documented<br>(HH:MM, 24 hr clock e.g. 22:31)	__________________________________<br>((HH:MM, 24 hr clock e.g. 22:31))

THE TIME YOU HAVE ENTERED IS BEFORE THE TIME THE DRAPE WAS APPLIED. PLEASE CHECK YOUR ENTRY AND EDIT ACCORDINGLY.

Who completed the observations and/or vital signs?	◯ Midwife<br>◯ Research midwife<br>◯ Student Midwife<br>◯ Doctor<br>◯ Intern<br>◯ Other
If other, please state:	__________________________________
Did you observe a blood pressure reading being taken?	◯ Yes<br>◯ No
If yes, provide the documented blood pressure reading (mmHg)	__________________________________<br>(e.g. 120/180)
or tick if not documented	◯ Not documented
Did you observe a pulse reading being taken?	◯ Yes<br>◯ No
If yes, provide the documented pulse reading (beats per minute)	__________________________________<br>(e.g. 82)
or tick if not documented	◯ Not documented
Did you observe uterine tone being assessed?	◯ Yes<br>◯ No
If yes, what was the documented uterine tone status?	◯ Soft<br>◯ Hard<br>◯ Not documented
Did you observe the practitioner assessing vaginal blood loss?	◯ Yes<br>◯ No

If yes, what was the documented vaginal blood loss status?<br><br>Select all that apply	☐ Heavy flow<br>☐ Large clots<br>☐ Trickle<br>☐ Normal<br>☐ Not documented
Is the blood swept into the funnel under the woman?	◯ Yes<br>◯ No
Did you observe the practitioner assessing the calibrated drape measurement line?	◯ Yes<br>◯ No
If yes, how is the funnel of the drape lying when reading the calibration lines?	◯ Hanging over the edge of the bed<br>◯ Flat on the bed
If flat on the bed, how are the calibration lines on the drape being read?	◯ Moved to the edge of the bed<br>◯ Visualised flat on the bed<br>◯ Other
If other, please state:	__________________________________
Was the cumulative calibrated drape measurement line reading documented?	◯ Yes<br>◯ No
If yes, what was the documented cumulative calibrated drape measurement line reading?	__________________________________<br>(ml)
4th set of observations	
Were the fourth set of observations and/or vital signs conducted?	◯ Yes<br>◯ No
Time observations and/or vital signs performed (HH:MM, 24 hr clock e.g. 22:31)	__________________________________<br>((HH:MM, 24 hr clock e.g. 22:31))
THE TIME YOU HAVE ENTERED IS BEFORE THE TIME THE DRAPE WAS APPLIED. PLEASE CHECK YOUR ENTRY AND EDIT ACCORDINGLY.	
Are the observations and/or vital signs being documented in the woman's clinical record?	◯ Yes<br>◯ No
If yes, where are the observations and/or vital signs documented?<br><br>Select all that apply	☐ Blood loss monitoring and WHO first response PPH bundle triggering chart<br>☐ Partograph<br>☐ Written in patient notes<br>☐ Early warning chart<br>☐ Treatment sheet<br>☐ Nursing Cardex<br>☐ Other
If other, please state:	__________________________________

Time observations documented (HH:MM, 24 hr clock e.g. 22:31)	____________ ((HH:MM, 24 hr clock e.g. 22:31))
THE TIME YOU HAVE ENTERED IS BEFORE THE TIME THE DRAPE WAS APPLIED. PLEASE CHECK YOUR ENTRY AND EDIT ACCORDINGLY.	
Who completed the observations and/or vital signs?	◯ Midwife<br>◯ Research midwife<br>◯ Student Midwife<br>◯ Doctor<br>◯ Intern<br>◯ Other
If other, please state:	____________
Did you observe a blood pressure reading being taken?	◯ Yes<br>◯ No
If yes, provide the documented blood pressure reading (mmHg)	____________ (e.g. 120/180)
or tick if not documented	◯ Not documented
Did you observe a pulse reading being taken?	◯ Yes<br>◯ No
If yes, provide the documented pulse reading (beats per minute)	____________ (e.g. 82)
or tick if not documented	◯ Not documented
Did you observe uterine tone being assessed?	◯ Yes<br>◯ No
If yes, what was the documented uterine tone status?	◯ Soft<br>◯ Hard<br>◯ Not documented
Did you observe the practitioner assessing vaginal blood loss?	◯ Yes<br>◯ No
If yes, what was the documented vaginal blood loss status?<br><br>Select all that apply	☐ Heavy flow<br>☐ Large clots<br>☐ Trickle<br>☐ Normal<br>☐ Not documented
Is the blood swept into the funnel under the woman?	◯ Yes<br>◯ No
Did you observe the practitioner assessing the calibrated drape measurement line?	◯ Yes<br>◯ No

If yes, how is the funnel of the drape lying when reading the calibration lines?	◯ Hanging over the edge of the bed<br>◯ Flat on the bed
If flat on the bed, how are the calibration lines on the drape being read?	◯ Moved to the edge of the bed<br>◯ Visualised flat on the bed<br>◯ Other
If other, please state:	__________________
Was the cumulative calibrated drape measurement line reading documented?	◯ Yes<br>◯ No
If yes, what was the documented cumulative calibrated drape measurement line reading?	__________________<br>(ml)

4. Outcomes

What time was the drape removed?<br>(HH:MM, 24 hr clock e.g. 22:31)	__________________<br>((HH:MM, 24 hr clock e.g. 22:31))
THE TIME YOU HAVE ENTERED IS BEFORE THE DRAPE WAS APPLIED. PLEASE CHECK YOUR ENTRY AND EDIT ACCORDINGLY.	
Who removed the drape?	◯ Midwife<br>◯ Student Midwife<br>◯ Research midwife<br>◯ Doctor<br>◯ Intern<br>◯ Other
If other, please state:	__________________
What was the drape weight?	__________________<br>(grams e.g. 1250)
What was the baby weight?	__________________<br>(grams e.g. 3380)
Please take a photo E-MOTIVE ID sticker, drape and baby weight sticker and upload it	
Was the woman alive?<br><br>Is the woman alive before transfer out of the labour ward?	◯ Yes<br>◯ No
Was the baby alive?<br><br>Is the baby alive before transfer out of the labour ward?	◯ Yes<br>◯ No

5. PPH incidence

Did the provider diagnose a PPH (≥ 500ml reading on the calibration drape or clinical judgement) whilst conducting your observations?<br><br>Given treatment dose of a uterotonic	◯ Yes<br>◯ No
If yes, what was the observed blood loss reading on the calibrated blood collection drape at time of PPH diagnosis?	__________________<br>(ml)
What was the cadre of the provider that diagnosed PPH?	◯ Midwife<br>◯ Research midwife<br>◯ Student Midwife<br>◯ Doctor<br>◯ Intern<br>◯ Other
If other, please state:	__________________
Was MOTIVE first response treatment provided?	◯ Yes<br>◯ No
What was the reason for triggering MOTIVE?	◯ Blood loss ≥ 500ml according to the calibrated drape<br>◯ Blood loss ≥ 300ml plus abnormal clinical feature<br>◯ Clinical judgement<br>◯ Other
If clinical judgement, please add details about worrying features	__________________<br>(e.g. clinical judgement, please add details about worrying features)
If other, please add details about worrying features	__________________
What time was MOTIVE triggered?<br>(HH:MM, 24 hr clock e.g. 22:31)	__________________<br>((HH:MM, 24 hr clock e.g. 22:31))

THE TIME YOU HAVE ENTERED IS BEFORE THE DRAPE WAS APPLIED. PLEASE CHECK YOUR ENTRY AND EDIT ACCORDINGLY.

When MOTIVE was triggered, was another/other staff member(s) alerted to come and help?	◯ YES - if practitioner is alone at the bedside<br>◯ YES - some practitioner(s) already at the bedside, other practitioner(s) called<br>◯ NO - no practitioners available<br>◯ NO - practitioners already present (no other practitioners called)
Did a doctor come to help deliver MOTIVE?	◯ Yes<br>◯ No<br>◯ Not applicable (doctor already present)

If yes, what time did the doctor arrive?
(HH:MM, 24 hr clock e.g. 22:31)

____
((HH:MM, 24 hr clock e.g. 22:31))

THE TIME YOU HAVE ENTERED IS BEFORE THE TIME PPH WAS DIAGNOSED. PLEASE CHECK YOUR ENTRY AND EDIT ACCORDINGLY.

Was an IV cannula already inserted prior to triggering MOTIVE (on admission or prior to birth)?
- ◯ Yes
- ◯ No

Trolley/carry case

Was the trolley/carry case brought to the bedside?
- ◯ Yes
- ◯ No

If yes, did this happen while waiting for a midwife or nurse or doctor to help deliver MOTIVE to arrive?
- ◯ Yes
- ◯ No
- ◯ Not applicable (no help was called)

Who brought the trolley/carry case to the bedside?
- ◯ Midwife
- ◯ Student midwife
- ◯ Research midwife
- ◯ Doctor
- ◯ Intern
- ◯ Other

If other, please state:

____

What time was the trolley/carry case brought to the bedside?
(HH:MM, 24 hr clock e.g. 22:31)

____
((HH:MM, 24 hr clock e.g. 22:31))

THE TIME YOU HAVE ENTERED IS BEFORE THE TIME PPH WAS DIAGNOSED. PLEASE CHECK YOUR ENTRY AND EDIT ACCORDINGLY.

Uterine massage

Was uterine massage performed?
- ◯ Yes
- ◯ No

If yes, did this happen while waiting for a midwife/nurse or doctor to help deliver MOTIVE to arrive?
- ◯ Yes
- ◯ No
- ◯ Not applicable (no help was called)

Who performed uterine massage?
- ◯ Midwife
- ◯ Student midwife
- ◯ Research midwife
- ◯ Doctor
- ◯ Intern
- ◯ Other

If other, please state:

____

What time did uterine massage start?
(HH:MM, 24 hr clock e.g. 22:31)

____
((HH:MM, 24 hr clock e.g. 22:31))

THE TIME YOU HAVE ENTERED IS BEFORE THE TIME PPH WAS DIAGNOSED. PLEASE CHECK YOUR ENTRY AND EDIT ACCORDINGLY.

What time did uterine massage stop?
(HH:MM, 24 hr clock e.g. 22:31)

____
((HH:MM, 24 hr clock e.g. 22:31))

THE TIME YOU HAVE ENTERED IS BEFORE THE TIME UTERINE MASSAGE STARTED. PLEASE CHECK YOUR ENTRY AND EDIT ACCORDINGLY.

Why was uterine massage stopped?

____
(e.g. uterus contracted, uterus not contracted but administering medicines, reason not said midwife was observed to stop uterine massage to check the perineum for laceration)

IV access, infusions and fluids

Was an IV cannula (1st IV line) or additional IV cannula (2nd IV line) inserted to help deliver MOTIVE?

- ◯ Yes
- ◯ No

If yes, did this happen while waiting for a midwife/nurse or doctor to help deliver MOTIVE to arrive?

- ◯ Yes
- ◯ No
- ◯ Not applicable (no help was called)

Who inserted the IV?

- ◯ Midwife
- ◯ Student midwife
- ◯ Research midwife
- ◯ Doctor
- ◯ Intern
- ◯ Other

If other, please state:

____

What time was the IV inserted?
(HH:MM, 24 hr clock e.g. 22:31)

____
((HH:MM, 24 hr clock e.g. 22:31))

THE TIME YOU HAVE ENTERED IS BEFORE THE TIME PPH WAS DIAGNOSED. PLEASE CHECK YOUR ENTRY AND EDIT ACCORDINGLY.

Was oxytocin IV infusion already attached for other reasons (augmentation or prevention of PPH)?

- ◯ Yes
- ◯ No

If yes, was this oxytocin infusion stopped?

- ◯ Yes
- ◯ No

Oxytocin: First line treatment for PPH - MOTIVE bundle

PLEASE NOTE - only record OXYTOCIN given for TREATMENT of PPH in this section

DO NOT include OXYTOCIN given prophylactically after birth

Was oxytocin given for the 1st line treatment of PPH?	◯ Yes<br>◯ No
If yes, did this happen while waiting for a midwife/nurse or doctor to help deliver MOTIVE to arrive?	◯ Yes<br>◯ No<br>◯ Not applicable (no help was called)
If yes, who started the 1st line oxytocin treatment?	◯ Midwife<br>◯ Student midwife<br>◯ Research midwife<br>◯ Doctor<br>◯ Intern<br>◯ Other
If other, please state:	__________________________
Was the same brand of oxytocin given for AMTSL as was given for treatment?	◯ Yes<br>◯ No
If no, what brand of oxytocin was administered for treatment?	◯ Climax Oxytocin<br>◯ Curtocin Injection<br>◯ DERM<br>◯ Evatocin<br>◯ Extocin<br>◯ Galaxy<br>◯ Labtocin<br>◯ Mark<br>◯ Oxymed<br>◯ Oxynir<br>◯ Oxyt<br>◯ Oxytocin 10<br>◯ Oxyzed 10IU<br>◯ Pitocin<br>◯ Pitons Oxytocin Injection<br>◯ Spec Oxytocin<br>◯ Syntocinon<br>◯ Utocin<br>◯ Vitocin-10<br>◯ Zytocin<br>◯ Not known<br>◯ Other
If other, please state:	__________________________
Was 1st line oxytocin treatment for PPH given as an oxytocin infusion?	◯ Yes<br>◯ No
Was a new oxytocin IV infusion started (1st infusion for treatment of PPH)?	◯ Yes<br>◯ No
What time did the 1st oxytocin infusion treatment for PPH start?<br>(HH:MM, 24 hr clock e.g. 22:31)	__________________________<br>((HH:MM, 24 hr clock e.g. 22:31))

THE TIME YOU HAVE ENTERED IS BEFORE THE TIME PPH WAS DIAGNOSED. PLEASE CHECK YOUR ENTRY AND EDIT ACCORDINGLY.

What was the 1st oxytocin infusion composition for PPH treatment?	◯ 20IU in 1000mL of fluid<br>◯ 40IU in 1000mL of fluid<br>◯ 10IU in 500mL of fluid<br>◯ 20IU in 500mL of fluid<br>◯ 40IU in 500mL of fluid<br>◯ Other
If other, please state:	__________________________
What fluid was used for the 1st oxytocin infusion?	◯ NaCl<br>◯ Ringer's lactate/Hartmann's<br>◯ Glucose<br>◯ Other
If other, please state:	__________________________
What time did the 1st oxytocin infusion treatment for PPH finish?<br>(HH:MM, 24 hr clock e.g. 22:31)	__________________________<br>((HH:MM, 24 hr clock e.g. 22:31))
Was any other medication(s) added to the 1st oxytocin infusion for PPH treatment?	◯ Yes<br>◯ No
If yes, please state the other medication(s):	__________________________
Was 1st line oxytocin treatment for PPH given as a syringe push bolus?	◯ Yes<br>◯ No
If syringe push bolus, what was the dose?	◯ 5 IU<br>◯ 10 IU<br>◯ Other
If other, please state:	__________________________
What time was the bolus oxytocin treatment given?<br>(HH:MM, 24 hr clock e.g. 22:31)	__________________________<br>((HH:MM, 24 hr clock e.g. 22:31))

THE TIME YOU HAVE ENTERED IS BEFORE THE TIME PPH WAS DIAGNOSED. PLEASE CHECK YOUR ENTRY AND EDIT ACCORDINGLY.

Was 1st line oxytocin treatment for PPH given IM?	◯ Yes<br>◯ No
If IM, what was the dose?	◯ 5 IU<br>◯ 10 IU<br>◯ Other
If other, please state:	__________________________

What time was the IM oxytocin treatment given?
(HH:MM, 24 hr clock e.g. 22:31)

____
((HH:MM, 24 hr clock e.g. 22:31))

Oxytocin: Maintenance infusion for PPH treatment

Was a maintenance oxytocin IV infusion started (2nd oxytocin infusion for PPH treatment)?

◯ Yes
◯ No

If yes, who started the maintenance oxytocin IV infusion for PPH treatment?

◯ Midwife
◯ Student midwife
◯ Research midwife
◯ Doctor
◯ Intern
◯ Other

If other, please state:

____

If yes, did this happen while waiting for a midwife/nurse or doctor to help deliver PPH first response to arrive?

◯ Yes
◯ No
◯ Not applicable (no help was called)

What time did the maintenance oxytocin infusion start (2nd oxytocin infusion for PPH treatment)?
(HH:MM, 24 hr clock e.g. 22:31)

____
((HH:MM, 24 hr clock e.g. 22:31))

THE TIME YOU HAVE ENTERED IS BEFORE THE TIME PPH WAS DIAGNOSED. PLEASE CHECK YOUR ENTRY AND EDIT ACCORDINGLY.

What was the maintenance oxytocin infusion (2nd oxytocin infusion for PPH treatment) composition?

◯ 20IU in 1000mL of fluid
◯ 40IU in 1000mL of fluid
◯ 10IU in 500mL of fluid
◯ 20IU in 500mL of fluid
◯ 40IU in 500mL of fluid
◯ Other

If other, please state:

____

What fluid was used for the maintenance oxytocin infusion (2nd oxytocin infusion for PPH treatment)?

◯ NaCl
◯ Ringer's lactate/Hartmann's
◯ Glucose
◯ Other

If other, please state:

____

What time did the maintenance oxytocin infusion finish (2nd oxytocin infusion for PPH treatment)?
(HH:MM, 24 hr clock e.g. 22:31)

____
((HH:MM, 24 hr clock e.g. 22:31))

THE TIME YOU HAVE ENTERED IS BEFORE THE FIRST INFUSION STARTED. PLEASE CHECK YOUR ENTRY AND EDIT ACCORDINGLY.

Tranexamic Acid - TXA

PLEASE NOTE - only record TXA given for TREATMENT of PPH in this section

DO NOT include TXA given prophylactically after birth

Was TXA administered?	◯ Yes<br>◯ No
If yes, did this happen while waiting for a midwife/nurse or doctor to help deliver MOTIVE to arrive?	◯ Yes<br>◯ No<br>◯ Not applicable (no help was called)
If yes, who administered TXA?	◯ Midwife<br>◯ Student midwife<br>◯ Research midwife<br>◯ Doctor<br>◯ Intern<br>◯ Other
If other, please state:	__________________________________
What brand of TXA was administered?	◯ Asinex<br>◯ Cyklokapron® IV 500<br>◯ Hemsamic<br>◯ Medsamic<br>◯ Pause Injection<br>◯ Prexam Injection<br>◯ Taxim<br>◯ Texakind Injection<br>◯ T-Nex Injection<br>◯ Tramax<br>◯ Tramic<br>◯ Tranexanaman<br>◯ Transic<br>◯ Tranxinex Injection<br>◯ Unotation<br>◯ Utramax<br>◯ Not known<br>◯ Other
If other, please state:	__________________________________
Was TXA given as a slow push or IV infusion?	◯ Slow push<br>◯ IV infusion
What was the TXA infusion composition?	◯ 500mg in 100mL of fluid<br>◯ 500mg in 200mL of fluid<br>◯ 1000mg in 100mL of fluid<br>◯ 1000mg in 200mL of fluid<br>◯ Other
If other, please state:	__________________________________
What fluid was used for the TXA infusion?	◯ NaCl<br>◯ Ringer's lactate/Hartmann's<br>◯ Glucose<br>◯ Volume expander<br>◯ Other
If other, please state:	__________________________________

What time did the slow push start?
(HH:MM, 24 hr clock e.g. 22:31)

____
((HH:MM, 24 hr clock e.g. 22:31))

THE TIME YOU HAVE ENTERED IS BEFORE THE TIME PPH WAS DIAGNOSED. PLEASE CHECK YOUR ENTRY AND EDIT ACCORDINGLY.

What time did the slow push finish?
(HH:MM, 24 hr clock e.g. 22:31)

____
((HH:MM, 24 hr clock e.g. 22:31))

THE TIME YOU HAVE ENTERED IS BEFORE THE SLOW PUSH STARTED. PLEASE CHECK YOUR ENTRY AND EDIT ACCORDINGLY.

What dose of TXA was administered via slow push?

- ◯ 500mg
- ◯ 1000mg (1g)

What time did the TXA IV infusion start?
(HH:MM, 24 hr clock e.g. 22:31)

____
((HH:MM, 24 hr clock e.g. 22:31))

THE TIME YOU HAVE ENTERED IS BEFORE THE TIME PPH WAS DIAGNOSED. PLEASE CHECK YOUR ENTRY AND EDIT ACCORDINGLY.

What time did the TXA IV infusion finish?
(HH:MM, 24 hr clock e.g. 22:31)

____
((HH:MM, 24 hr clock e.g. 22:31))

THE TIME YOU HAVE ENTERED IS BEFORE THE TIME PPH WAS DIAGNOSED. PLEASE CHECK YOUR ENTRY AND EDIT ACCORDINGLY.

Was any other medication added to the TXA infusion?

- ◯ Yes
- ◯ No

If yes, what other medication added?
Please give the name and dose

____

Uterotonics

PLEASE NOTE - only record uterotonics given for TREATMENT of PPH in this section

DO NOT include uterotonics given prophylatically after birth

Were any other uterotonics given?

- ◯ Yes
- ◯ No

If yes, did this happen while waiting for midwife/nurse or doctor to help deliver MOTIVE to arrive?

- ◯ Yes
- ◯ No
- ◯ Not applicable (no help was called)

If yes, who administered the other uterotonic?

- ◯ Midwife
- ◯ Student midwife
- ◯ Research midwife
- ◯ Doctor
- ◯ Intern
- ◯ Other

If other, please state:	__________________
Was misoprostol administered to treat the PPH?	◯ Yes<br>◯ No
Dose of misoprostol	◯ 600mcg<br>◯ 800mcg<br>◯ 1000mcg<br>◯ Other
If other, please specify in mcg:	__________________
Route of misoprostol<br><br>Select all that apply	☐ Per rectal<br>☐ Oral or sublingual
Time misoprostol given<br>(HH:MM, 24 hr clock e.g. 22:31)	__________________<br>((HH:MM, 24 hr clock e.g. 22:31))
THE TIME YOU HAVE ENTERED IS BEFORE THE TIME PPH WAS DIAGNOSED. PLEASE CHECK YOUR ENTRY AND EDIT ACCORDINGLY.	
Was syntometrine administered to treat the PPH?	◯ Yes<br>◯ No
Dose of syntometrine	◯ 500 mcg/5 IU in 1 mL<br>◯ Other
If other, please specify in mcg:	__________________
Route of syntometrine<br><br>Select all that apply	☐ IV<br>☐ IM
Time syntometrine given<br><br>(HH:MM, 24 hr clock e.g. 22:31)	__________________
THE TIME YOU HAVE ENTERED IS BEFORE THE TIME PPH WAS DIAGNOSED. PLEASE CHECK YOUR ENTRY AND EDIT ACCORDINGLY.	
Was any other uterotonic administered to treat the PPH or additional doses of the uterotonics mentioned before?	◯ Yes<br>◯ No
Please specify name of uterotonic, including dose and route:	__________________
Genital tract examination	
Was there an examination of the genital tract done?	◯ Yes<br>◯ No

If yes, please select type of examination performed:	◯ Abdominal examination only<br>◯ External inspection only<br>◯ Internal examination with gloves for tears<br>◯ Internal examination with gloves only for tears and the uterus for clots<br>◯ Internal and speculum<br>◯ Speculum only<br>◯ Other
If other, please state:	__________________________
If yes, who performed the examination?	◯ Midwife<br>◯ Student Midwife<br>◯ Research Midwife<br>◯ Doctor<br>◯ Intern<br>◯ Other
If other, please state:	__________________________
What time did the examination start?<br>(HH:MM, 24 hr clock e.g. 22:31)	__________________________<br>((HH:MM, 24 hr clock e.g. 22:31))
THE TIME YOU HAVE ENTERED IS BEFORE THE TIME PPH WAS DIAGNOSED. PLEASE CHECK YOUR ENTRY AND EDIT ACCORDINGLY.	
What time did the examination finish?<br>(HH:MM, 24 hr clock e.g. 22:31)	__________________________<br>((HH:MM, 24 hr clock e.g. 22:31))
THE TIME YOU HAVE ENTERED IS BEFORE THE EXAMINATION STARTED. PLEASE CHECK YOUR ENTRY AND EDIT ACCORDINGLY.	
What were the documented findings?	◯ Atonic uterus<br>◯ Tear (vaginal, cervical, perineal)<br>◯ Not documented<br>◯ Other
If other, please state:	__________________________
Suturing	
Did the perineum require suturing?	◯ Yes<br>◯ No
If yes, did this happen while waiting for a midwife/nurse or doctor to help deliver MOTIVE to arrive?	◯ Yes<br>◯ No<br>◯ Not applicable (no help was called)

If yes, who performed the suturing?	◯ Midwife<br>◯ Student midwife<br>◯ Research midwife<br>◯ Doctor<br>◯ Intern<br>◯ Other
If other, please state:	__________________________________
Was suturing escalated due to excess bleeding from a laceration?	◯ Yes<br>◯ No
What time did suturing start?<br>(HH:MM, 24 hr clock e.g. 22:31)	__________________________________<br>((HH:MM, 24 hr clock e.g. 22:31))
THE TIME YOU HAVE ENTERED IS BEFORE THE TIME PPH WAS DIAGNOSED. PLEASE CHECK YOUR ENTRY AND EDIT ACCORDINGLY.	
What time did suturing finish?<br>(HH:MM, 24 hr clock e.g. 22:31)	__________________________________<br>((HH:MM, 24 hr clock e.g. 22:31))
THE TIME YOU HAVE ENTERED IS BEFORE THE SUTURING STARTED. PLEASE CHECK YOUR ENTRY AND EDIT ACCORDINGLY.	
Bladder examination	
Was a catheter inserted?	◯ Yes<br>◯ No
If yes, did this happen while waiting for a midwife/nurse or doctor to help deliver MOTIVE to arrive?	◯ Yes<br>◯ No<br>◯ Not applicable (no help was called)
If yes, who inserted the catheter?	◯ Midwife<br>◯ Student midwife<br>◯ Research midwife<br>◯ Doctor<br>◯ Intern<br>◯ Other
If other, please state:	__________________________________
What type of catheter was inserted?	◯ In-out-catheter to drain bladder only<br>◯ Insert Foleys catheter with urine bag
What time was the catheter inserted?<br>(HH:MM, 24 hr clock e.g. 22:31)	__________________________________<br>((HH:MM, 24 hr clock e.g. 22:31))
THE TIME YOU HAVE ENTERED IS BEFORE THE TIME PPH WAS DIAGNOSED. PLEASE CHECK YOUR ENTRY AND EDIT ACCORDINGLY.	

If no, why wasn't the bladder emptied?	◯ Woman voided, prior to birth<br>◯ In-out-catheter to drain bladder only, prior to birth<br>◯ Insert Foley's catheter and urine bag to drain bladder, prior to birth<br>◯ No actions taken to drain bladder prior to birth<br>◯ Other
If other, please state:	__________________________________
Cradle device	
Was a cradle device used?	◯ Yes<br>◯ No
Escalated management	
If the bleeding continued, was the PPH treatment escalated after MOTIVE was delivered?	◯ Yes<br>◯ No
If yes, who escalated management of PPH?	◯ Midwife<br>◯ student midwife<br>◯ Research midwife<br>◯ Doctor<br>◯ Intern<br>◯ Other
If other, please state:	__________________________________
Please explain what happened, please include the reason for any delays in escalation, including if there was a delay due to waiting for a more senior obstetric doctor to arrive.	____
What was the medical management of the escalation?<br><br>Select all that apply	☐ Uterine balloon tamponade (UBT)<br>☐ Condom & Foley's catheter<br>☐ Laparotomy<br>☐ Hysterectomy<br>☐ NASG<br>☐ Transferred out of the facility<br>☐ Other
If other, please state:	__________________________________
What time was the decision made to escalate? (HH:MM, 24 hr clock e.g. 22:31)	__________________________________<br>((HH:MM, 24 hr clock e.g. 22:31))
THE TIME YOU HAVE ENTERED IS BEFORE THE TIME PPH WAS DIAGNOSED. PLEASE CHECK YOUR ENTRY AND EDIT ACCORDINGLY.	
What time did the next intervention start? (HH:MM, 24 hr clock e.g. 22:31)	__________________________________<br>((HH:MM, 24 hr clock e.g. 22:31))

Were all the observed treatments documented on the blood loss monitoring and WHO first response PPH bundle triggering chart?	◯ Yes<br>◯ No
Which treatments were documented?<br><br>Select all that apply	☐ Uterine Massage<br>☐ Oxytocin (PPH treatment only)<br>☐ 1st dose of TXA<br>☐ IV fluids<br>☐ Examination(genital tract)<br>☐ Escalation<br>☐ Misoprostol (PPH treatment only)<br>☐ Syntometrine<br>☐ Ergometrine<br>☐ 2nd dose of TXA
THE TIME YOU HAVE ENTERED IS BEFORE THE TIME PPH WAS DIAGNOSED. PLEASE CHECK YOUR ENTRY AND EDIT ACCORDINGLY.	
Number of staff attending the PPH first response treatment using MOTIVE	
Midwives or nurses	__________________________
Junior doctors	__________________________
Senior doctors	__________________________
Other	__________________________
If other, specify who:	__________________________
Reflecting on early detection of PPH and MOTIVE delivery	
Overall, were any delays or opportunities for improvement in the management of the PPH using MOTIVE bundle 'first response' treatment?	◯ No delays or opportunities for improvement observed<br>◯ Opportunities for improvement in the management of PPH using MOTIVE observed
Please describe where management of PPH could be improved:<br><br>Please do not write 'stated above', please describe in detail	________________________________
Overall, were there any issues with obtaining or administering any of the MOTIVE bundle treatments?	◯ No issues observed with obtaining or administering any of the MOTIVE bundle interventions<br>◯ Issues observed with obtaining or administering any of the MOTIVE bundle interventions
Please describe where there were problems with obtaining or administering PPH treatments:<br>Please do not write ‘stated above’, please describe in detail	________________________________
