# Supplementary material for: Early detection and a treatment bundle strategy for postpartum haemorrhage: a mixed-methods process evaluation
Source: Lancet Glob Health. 2025 Jan 29;13(2):e329–44. doi: 10.1016/S2214-109X(24)00454-6 (PMC11782988; doi:10.1016/S2214-109X(24)00454-6)
Supplement: Equitable Partnership Declaration [file mmc3.pdf]

THE LANCET
Global Health

## Supplementary appendix 3

This Equitable Partnership Declaration (EPD) was submitted by the authors, and we reproduce it as supplied. It has not been peer reviewed. *The Lancet*'s editorial processes have not been applied to the EPD.

Supplement to: Bohren MA, Miller S, Mammoliti K-M, et al. Early detection and a treatment bundle strategy for postpartum haemorrhage: a mixed-methods process evaluation. *Lancet Glob Health* 2025; **13:** e329–44.

**Equitable Partnership Declaration questions**

This Equitable Partnership Declaration is a statement being published online alongside papers at *The Lancet Global Health*, as a separate appendix, to allow researchers to describe how their work engages with researchers, communities, and environments in the countries of study. This is part of our broader goal to decolonise global health, handing control and leadership of research to academics and clinicians who are based in the regions of study, and to affected communities.

Please answer all questions with as much detail as possible, noting that all included information will be published open-access and it will be freely available online to all who wish to read it. If a question does not apply to your study, please state "Not applicable".

The format of and questions in this statement are currently in a pilot phase. Please email Dr Kate McIntosh (Kate.McIntosh@lancet.com; deputy editor) with any feedback, particularly if you find any questions unclear.

*Researcher considerations*

1. Please detail the involvement that researchers who are based in the region(s) of study had during a) study design; b) clinical study processes, such as processing blood samples, prescribing medication, or patient recruitment; c) data interpretation; and d) manuscript preparation, commenting on all aspects. If they were not involved in any of these aspects, please explain why.

*This question is intended for international partnerships; if all your authors are based in the area of study, this question is not applicable.*

*This should include a thorough description of their leadership role(s) in the study. Are local researchers named in the author list or the acknowledgements, or are they not mentioned at all (and, if not, why)? Please also describe the involvement of early career researchers based in the location of the study. Some of this information might be repeated from the Contributors section in the manuscript. Note: we adhere to ICMJE authorship criteria when deciding who should be named on a paper.*

**a) Study design:**

The study authors include researchers from the following teams:

- Study sites:
  - Nigeria: HG, AAW, MB, IUT, MU
  - South Africa: SF, NM, GJH, MSM, EM, SM
  - Kenya: ZQ, AO, GG, JO
  - Tanzania: FAB, AB, MS
- Trial sponsor (University of Birmingham, UK): KMM, KNS, LB, CLE, AD, AC
- Mixed-methods team (University of Melbourne, University of California San Francisco, University College London, Jhpiego): MAB, SM, GF, SA, TMS, CE, FL
- Other collaborators (Concept Foundation, WHO): AMG, FA, OTO, IG

MAB, SM, HG, SF, NM, GJH, ZQ, FAB, CE, IG, AC, and FL conceptualised and designed this study. HG, AAW, SF, NM, GJH, ZQ, AO, GG, and FAB led the submission to local ethics reviews, approvals, registration, and administrative clearances in Nigeria, South Africa, Kenya and Tanzania. HG, AAW, ZQ, JO, and FAB led the data collection teams in each country, supported by MAB, SM, KMM, GF, SA, and FL. Those named in the Acknowledgements section contributed to data collection and/or

study implementation, but did not meet the ICMJE criteria for authorship. MAB, SM, KMM, HG, SF, NM, GJH, ZQ, FAB, GF, SA, AO, GG, AAW, MB, IUT, MU, MSM, EM, SM, JO, AB, MS and FL were involved in data curation and project administration. MAB, SM and FL supervised the process evaluation implementation.

**b) Clinical study processes:**
The E-MOTIVE simulation-based training and drills were developed by Jhpiego and CE, with technical input from KMM, AC, IG, FA, AMG and those listed in the acknowledgements. Training was delivered to master trainers in each country by CE and then cascaded to the intervention sites by local trainers (listed as co-authors or in the Acknowledgements: Isabella Ochieng, Jennyfer Oluyemisi Don-Aki, Comfort Okpe, Hannatu Abdullahi, Tiba Gaudiosa, Chrisostom Lipingu, Jimkelly Mugambi, Polycarp Oyoo, Adeosun Love Funmi, Rahmatu Yusuf, Abubakar Fatima, Arigbede Ololade, Masumbuko Sambusa, Akwinata Banda, Sara Willemse, Fawzia Samuels) and the country hub teams. Supportive supervision after training was provided by local trainer to support fidelity to the intervention. HG, AAW, MB, IUT, MU, SF, NM, GJH, MSM, EM, SM, ZQ, AO, GG, JO, FAB, and MS were responsible for overseeing study processes in each site, with support from those listed in the acknowledgements. MAB, SM, GF, SA, FL, KMM, HG, AAW, SF, NM, GJH, MSM, ZQ, JO, and FAB trained those collecting data for the process evaluation.

**c) Data interpretation:**
Data management was coordinated by MAB, FL, KMM, GF, and SA for the multi-country databases, and led at a country-level by HG, AAW, SF, NM, GJH, MSM, EM, ZQ, JO and FAB. MAB, SM, KMM, GF, SA, TMM and FL cleaned the datasets and conducted initial data analysis and visualisation with support from CLE. Initial data analysis was discussed and interpreted closely with HG, AAW, SF, NM, GJH, MSM, ZQ, AO, GG, and FAB first at a country-level, and then across countries with the input from other co-authors.

**d) Manuscript preparation:**
MAB and FL wrote the original draft. All authors contributed to interpretation of results, and revised the manuscript. MAB and FL accept responsibility for the decision to submit for publication.

2. Were the data used in your study collected by authors named on the paper, or have they been extracted from a source such as a national survey? ie, is this a secondary analysis of data that were not collected by the authors of this paper. If the authors of this paper were not involved in data collection, how were data interpreted with sufficient contextual knowledge?

   The Lancet Global Health *believe contextual understanding is crucial for informed data analysis and interpretation.*

   Data used in this study consisted of observations of health workers providing clinical care to women with vaginal birth and PPH (if it occurred), surveys with health workers, and qualitative interviews with health workers. All data presented in this paper were collected for the purposes of this study, and is therefore primary data. Those named as co-authors are from the study context and provided critical insights into interpretation of the study context and results.

3. How was funding used to remunerate and enhance the skills of researchers and institutions based in the area(s) of study? And how was funding used to improve research infrastructure in the area of study?

*Potentially effective investments into long-term skills and opportunities within institutions could include training or mentorship in analytical techniques and manuscript writing, opportunities to lead all or specific aspects of the study, financial remuneration rather than requiring volunteers, and other professional development and educational opportunities.*

*Improvements to research infrastructure could be funding of extended trial designs (such as platform trials) and use of master protocols to enable these designs, establishment of long-term contracts for research staff, building research facilities, and local control of funding allocation.*

| |
|---|
| **Skills:**<br>The Kenya, Nigeria, South Africa and Tanzania teams worked closely on all phases of the study design and intervention implementation with the mixed-methods team, trial sponsor, and other collaborators, and strengthened research capacity related to protocol design, research ethics considerations (particularly for cluster randomised trials and qualitative/mixed-methods evaluation), data management and analysis, academic writing, and preparation of study derivative products and implementation into policy (e.g. the WHO PPH guidelines). |
| **Research infrastructure:**<br>The E-MOTIVE project has contributed to strengthening research capacity in the context of clinical trials, mixed-methods evaluation of trials, and economic evaluation at the four study sites, including conducting and generating evidence on priority areas of maternal health conditions and implementation of effective clinical practices. Following the demonstration of effectiveness of the E-MOTIVE intervention, all control sites moved to an implementation phase, to ensure that all study sites have access to life-saving interventions and training. |

4. How did you safeguard the researchers who implemented the study?

*Please describe how you guaranteed safe working conditions for study staff, including provision of appropriate personal protective equipment, protection from violence, and prevention of overworking.*

| |
|---|
| All institutions involved in the implementation of the E-MOTIVE project (Bayero University, University of Cape Town, KwaZulu-Natal Department of Health, University of the Witwatersrand, University of Nairobi, Muhimbili University of Health and Allied Sciences, University College London, Jhpiego, University of Birmingham, and WHO) have policies on safe working conditions, equal employment, human resources, and protection from violence, overworking and exploitation. Data collectors within the health facilities and study staff during monitoring visits were provided with appropriate transportation and personal protection (e.g. face masks, hand sanitizer during COVID-19). |

*<u>Benefits to the communities and regions of study</u>*

5. How does the study address the research and policy priorities of its location?
*How were the local priorities determined and then used to inform the research question? Who decided which priorities to take forward? Which elements of the study address those priorities?*

| |
|---|
| Kenya, Nigeria, South Africa and Tanzania represent four countries with high burdens of maternal mortality due to postpartum haemorrhage. Addressing these maternal health issues are included in the national health strategies. Coverage of the E-MOTIVE intervention components (early |

detection of PPH using a blood loss collection drape, with treatment of uterine massage, oxytocics, intravenous fluids, and tranexamic acid) is variable in these settings, despite being recommended in the WHO PPH guidelines. As a result, many women in these settings are dying or experiencing worse health and well-being outcomes from preventable and treatable blood loss around the time of birth. Bundled approaches to care provision in other health areas (e.g. stroke, sepsis) have been proven to be effective. At the time of designing the study, there was limited evidence from settings with high burden of PPH as to whether early detection and bundled approaches to care would improve outcomes. The E-MOTIVE study was thus conceptualised in this context to test the intervention effectiveness, and generate evidence to inform global and national policy, health programmes and clinical practice.

6. How will research products be shared in the community of study?
*For instance, will you be providing written or oral layperson summaries for non-academic information sharing? Will study data be made available to institutions in the region(s) of study?* The Lancet Global Health *encourages authors to translate the summary (abstract) into relevant languages after paper editing; do you intend to translate your summary?*

Findings from the E-MOTIVE study have been disseminated at a national level to all key stakeholders in Kenya, Nigeria, South Africa and Tanzania, and with all 78 trial intervention and control sites. Lay person summaries in local languages have been prepared for the E-MOTIVE programme of work. The positive findings from the E-MOTIVE study have already been translated into updated WHO guideline recommendations (2023), which are now being adapted and implemented at the country level in the four study countries and beyond. For example, the control sites are now implementing the E-MOTIVE intervention to ensure that the calibrated drape and MOTIVE bundle are implemented in practice. Moreover, each study country is now in different phases of country-driven, donor-supported scale-up of E-MOTIVE. For example, in KwaZulu-Natal province, South Africa, the E-MOTIVE approach is currently being implemented in all designated health facilities providing labour and birth services, with a view to implementing E-MOTIVE as standard practice.

7. How were individuals, communities, and environments protected from harm?

a) *How did you ensure that sensitive patient data was handled safely and respectfully? Was there any potential for stigma or discrimination against participants arising from any of the procedures or outcomes of the study?*

The identities of all study participants are anonymous and any confidential information shared in the survey or qualitative interview responses has been removed from the text.

b) *Might any of the tests be experienced as invasive or culturally insensitive?*

Not applicable

c) *How did you determine that work was sensitive to traditions, restrictions, and considerations of all cultural and religious groups in the study population?*

Not applicable – the E-MOTIVE intervention components are all considered as standard practices of care.

d) *Were biowaste and radioactive waste disposed of in accordance with local laws?*

| |
|---|
| Not applicable |

e) *Were any structures built that would have impacted members of the community or the environment (such as handwashing facilities in a public space)? If so, how did you ensure that you had appropriate community buy-in?*

| |
|---|
| Not applicable |

f) *How might the study have impacted existing health-care resources (such as staff workloads, use of equipment that is typically employed elsewhere, or reallocation of public funds)?*

| |
|---|
| Delivering the E-MOTIVE intervention may have increased staff workloads – we evaluated this as part of the process evaluation and found that >69% of survey participants did not think that it increased their workload (Figure 2). Our process evaluation results show that health workers in general found the intervention highly acceptable, improved care for women, and reduced work-related stress (Figure 2). |

8. Finally, please provide the title (eg, Dr/Prof, Mr/Mrs/Ms/Mx), name, and email address of an author who can be contacted about this statement. This can be the corresponding author.


| |
|---|
| **Name:** Dr Meghan A. Bohren<br>**Email:** meghan.bohren@unimelb.edu.au |
